# A potassium-based single-atom catalyst enables acute lung injury immunotherapy in mice by inhibiting macrophage pyroptosis

**DOI:** 10.1038/s41467-026-76331-8

**Published:** 2026-08-04

**Authors:** Xiangyu Lu, Xuan Shi, Yanmin Jian, Cai Sun, Lijie Mao, Si Chen, Yuanru Zhao, Fang Huang, Ji Lu, Zhiyi Song, Guanning Liu, Xin Li, Yongjun Chen, Ke Li, Xin Lv, Jianlin Shi

**Affiliations:** 1 Department of Anesthesiology, Shanghai Pulmonary Hospital, School of Medicine, Tongji University, Shanghai, China; 2 Department of Ophthalmology, Shanghai Tenth People’s Hospital, Shanghai Frontiers Science Center of Nanocatalytic Medicine, Clinical Center For Brain And Spinal Cord Research, School of Medicine, Tongji University, Shanghai, China; 3 Shanghai Institute of Ceramics, Chinese Academy of Sciences, Shanghai, China; 4 School of Materials Science and Engineering, Hainan University, Haikou, China; 5 Shanghai Skin Disease Hospital, School of Medicine, Tongji University, Shanghai, China

**Keywords:** Biomedical materials, Nanomedicine, Mucosal immunology, Inflammatory diseases, Cell death and immune response

## Abstract

Acute lung injury (ALI), which can have a mortality rate approaching 40%, lacks effective therapies. Macrophage pyroptosis represents a pro-inflammatory event and a potential therapeutic target. Here, we report a single-atom immunomodulator, a potassium-based single-atom catalyst (K-SAC) with K–N_4_ sites, that suppresses macrophage pyroptosis for ALI therapy through antioxidant catalysis. Constructed from biocompatible potassium, the most abundant intracellular metal in humans, K-SAC displays potent superoxide dismutase- and catalase-like activities, with the underlying mechanisms revealed by density functional theory simulations. Following preferential macrophage uptake, K-SAC scavenges reactive oxygen species, downregulates gasdermin D and its N-terminal fragment, activates endosomal sorting complexes required for transport-mediated membrane repair, and promotes membrane phospholipid remodeling, thereby effectively inhibiting macrophage pyroptosis. Notably, this catalytic membrane repair mechanism offers a versatile strategy for restoring membrane integrity. Such catalytic inhibition of pyroptosis preserves pulmonary immune homeostasis and ameliorates lipopolysaccharide- or cecal ligation and puncture-induced ALI, establishing a catalytic immunotherapeutic approach using a physiologically abundant element for therapeutic applications.

## Abstract (web summary)

Acute lung injury (ALI) involves promotion of inflammatory pathways and macrophage pyroptosis. Here the authors propose a macrophage targeting potassium containing catalytic particle which reduces ALI through scavenging reactive oxygen species, downregulation of gasdermin D and inhibition of macrophage pyroptosis.

## Introduction

Acute lung injury (ALI) and its more severe form, acute respiratory distress syndrome (ARDS), are critical conditions with mortality rates approaching 40%^1,2^. The underlying pathology involves profoundly dysregulated inflammatory cascades that damage lung tissue and impair respiratory function. Increasing incidence rates, driven by factors such as environmental pollution, aging populations, and respiratory infections, contribute to significant healthcare burdens. Notably, 15–30% of hospitalized COVID-19 patients progress to ARDS^3^, emphasizing the need for improved treatments. Current approaches, including mechanical ventilation and pharmacological therapies, have major limitations—ventilation can exacerbate lung injury, while drugs often have toxicity concerns and narrow therapeutic windows. Therefore, safer and more effective interventions are urgently needed.

Immunotherapy holds promising prospects in the treatment of ALI^4^, given that immune dysregulation is a hallmark of ALI pathogenesis^5^. As a key component of the innate immune system, macrophages play important roles in pathogen defense, tissue repair, and homeostasis maintenance^6–9^. During the progression of ALI, activated alveolar macrophages act as the first line of defense against pathogenic microorganisms. They exert anti-inflammatory effects by recognizing and eliminating pathogenic microorganisms^10^, while simultaneously undergoing forms of cell death, such as apoptosis and pyroptosis^11^. Despite several macrophage-targeted approaches being developed, none have achieved clinical success for ALI^12,13^.

The progression of ALI involves damage at multiple levels. Systemically, activated macrophages release cytotoxic mediators, such as reactive oxygen species (ROS) and proinflammatory cytokines, which disrupt the alveolar-capillary barrier. At the cellular level, ROS induces lipid peroxidation, damaging membrane integrity and fueling inflammation^14–17^. This process triggers pyroptosis, a form of programmed cell death characterized by membrane rupture and the release of pro-inflammatory signals, creating a self-perpetuating cycle of injury^18–22^. Accordingly, integrated therapeutic strategies targeting ROS scavenging, pyroptosis inhibition, and membrane stabilization offer a promising paradigm to disrupt this cascade and restore pulmonary homeostasis. However, pyroptosis-targeting nanotherapeutics remains in its infancy despite their therapeutic promise.

While endogenous enzymes like superoxide dismutase (SOD), catalase (CAT), peroxidase (POD), and glutathione peroxidase (GSH-Px) exhibit high catalytic activity, their clinical translational potential as therapeutic agents is hindered by inherent limitations: structural instability, high production costs, and challenges in large-scale manufacturing^23,24^. Antioxidant enzyme-mimicking catalysts have recently demonstrated therapeutic potential against inflammatory disorders, such as neuroinflammation and osteoarthritis^25–28^. However, a significant barrier to their clinical use is that most catalysts rely on transition metals (e.g., Fe, Cu, and Mn), which pose risks of metal ion toxicity that can adversely impact multiple physiological systems^29,30^. Therefore, developing catalytic materials that combine potent catalytic activity with minimal metal toxicity is essential for successful clinical translation. To address this challenge, we turned to physiologically abundant s-block elements (Ca, K, Na, and Mg). Although s-block metals have traditionally been considered catalytically inert—owing to the lack of suitable partially occupied d orbitals—advances in single-atom catalyst (SAC) engineering indicate that catalytic behavior is dictated primarily by the electronic structure of the isolated metal active site and its local coordination environment^31,32^. On this basis, we hypothesized that precisely tailoring the coordination sphere to tune the *s*- and *p*-orbital characteristics of s-block metals could optimize adsorption of reaction intermediates and facilitate electron-transfer processes, thereby conferring potent antioxidant catalytic activity. Potassium (K) is the most abundant intracellular cation (~150 mM), substantially exceeding concentrations of other metals (typically <10 mM). Unlike calcium, which is largely sequestered in bone and teeth, potassium mainly distributes in cells, making it ideally suited for intracellular therapeutic applications. Moreover, according to National Academy of Medicine guidelines, its recommended daily intake (2.3–3.0 g) is two orders of magnitude higher than that of essential transition metals (e.g., Fe, Mn, Cu, and Zn; each <0.02 g)^33^. Leveraging this inherent biosafety profile, we designed and synthesized a potassium-based catalytic system that offers a substantially broader therapeutic margin while minimizing the risk of metal-induced toxicity.

In this study, we report a potassium-based single-atom catalyst (K-SAC) as a therapeutic platform for ALI immunotherapy through inhibition of macrophage pyroptosis. The administered potassium dose (1.9 μg/kg/day) remains well within the National Academy of Medicine’s recommended daily intake, ensuring excellent safety margins. Biodistribution studies confirm that K-SAC preferentially accumulates in alveolar macrophages (the primary source of ROS in early lung injury), enabling targeted therapy. Unlike conventional antioxidants, K-SAC combines a non-toxic metal composition with multimodal action: efficient ROS scavenging, suppression of gasdermin D (GSDMD) and its N-terminal fragment (GSDMD-NT), and activation of the endosomal sorting complexes required for transport (ESCRT)-mediated membrane repair. Moreover, this study demonstrates antioxidant activity in s-block metal SACs, opening a frontier for developing inherently biocompatible catalysts that harness physiologically abundant elements (e.g., K, Na, Ca, and Mg) for therapeutic applications.

## Results

### Synthesis and characteristics of K-SAC

**Fig. 1:**
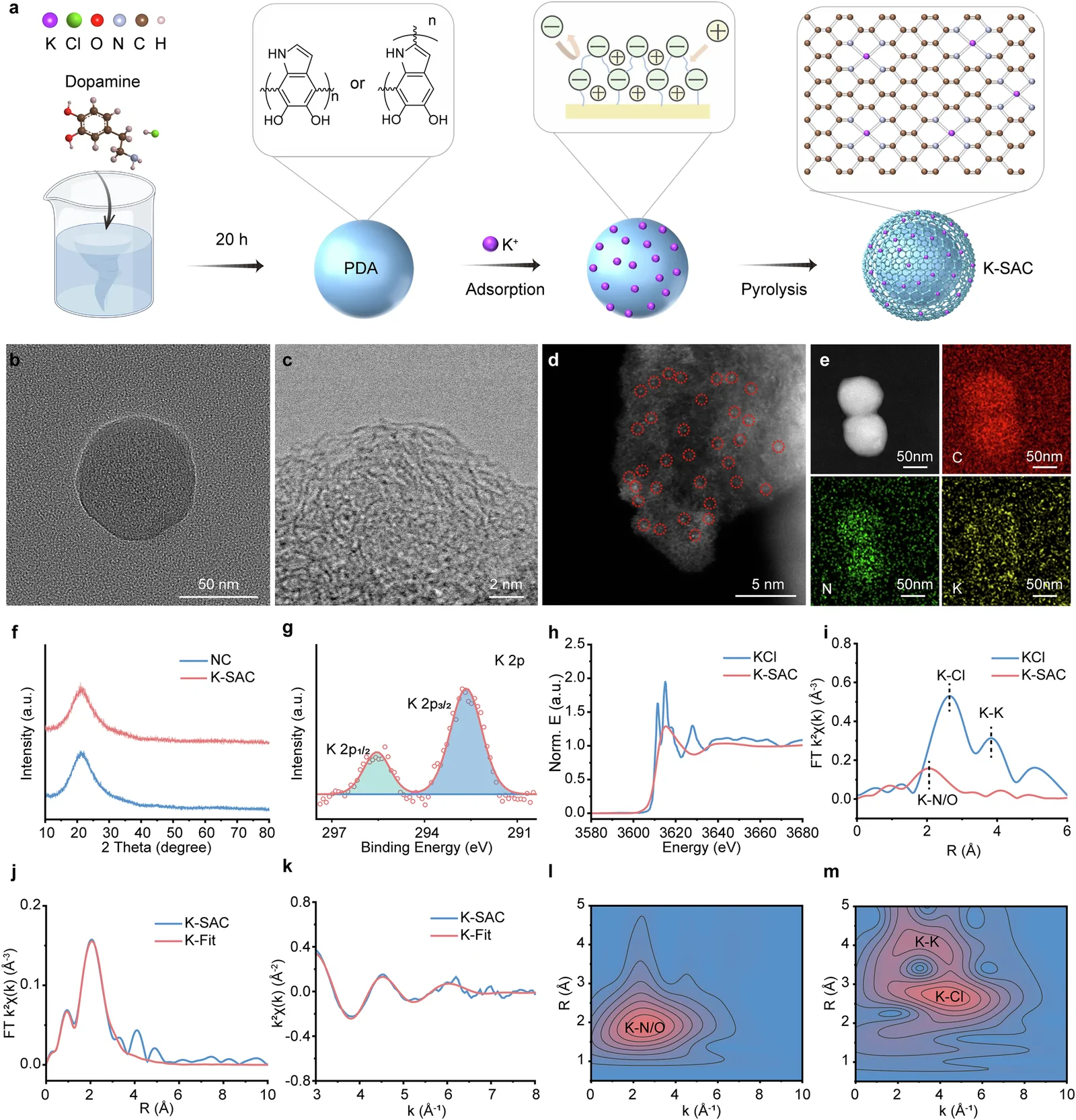
Synthesis and characterization of K-SAC. **a** Schematic illustration of K-SAC synthesis. Created by figdraw.com. **b** TEM image of K-SAC (*n* = 3 samples with similar results). **c** BF-STEM image of K-SAC (*n* = 3 samples with similar results). **d** AC HAADF-STEM image of K-SAC (*n* = 3 samples with similar results). **e** HAADF-STEM image and corresponding EDS mappings of K-SAC: C (red), N (green), K (yellow) (*n* = 3 samples with similar results). **f** XRD patterns of K-SAC and NC. **g** High-resolution K 2*p* XPS of K-SAC. **h** Normalized XANES spectra of K-SAC and KCl. **i** FT-EXAFS results of K-SAC and KCl. EXAFS fitting for K-SAC in R space (**j**) and in k-space (**k**). Wavelet transformation of K-SAC (**l**) and KCl (**m**). Source data are provided as a Source Data file.

The synthetic protocol of K-SAC is illustrated in Fig. 1a. Initially, polydopamine (PDA) nanospheres were fabricated via dopamine self-assembly, followed by electrostatic incorporation of K ions into the nanospheres. The final K-SAC was obtained through high-temperature pyrolysis under a nitrogen atmosphere. For comparison, a K-free N-doped carbon (NC) was prepared by the same procedure as K-SAC without the addition of KCl.

Transmission electron microscopy (TEM) images show that both PDA and K-SAC present a well-defined spherical morphology (Fig. 1b and Supplementary Fig. 1). Dynamic light scattering (DLS) analysis further reveals that K-SAC is dispersed with a hydrodynamic diameter of ~200 nm (Supplementary Fig. 2). Additionally, nitrogen sorption isotherm experiments illustrate that K-SAC has an abundant mesoporous structure, thereby favoring the exposure of catalytically active sites (Supplementary Fig. 3). Bright field STEM (BF-STEM) image and selected area electron diffraction pattern confirm the absence of crystalline phases in K-SAC, matching well with the X-ray diffractometer results (Fig. 1c, f, and Supplementary Fig. 4). Furthermore, aberration-corrected (AC) HAADF-STEM image demonstrates the atomic-level dispersion of K in K-SAC, as evidenced by the presence of isolated bright spots (Fig. 1d). Besides, detailed analysis of corresponding energy-dispersive X-ray spectroscopy (EDS) mappings reveals a uniform distribution of the K element, further confirming the presence of K single atoms (Fig. 1e). The peak of K is also detected in X-ray photoelectron spectroscopy, which indicates the successful doping of K into the C-N matrix (Fig. 1g). The potassium content is quantified at 2.33 wt% by inductively coupled plasma optical emission spectrometry.

### Atomic structure analysis of K-SAC

To characterize the structural architecture of K-SAC, a series of examinations were subsequently employed. Fourier transform infrared spectroscopy (FTIR) spectra reveal that K-SAC possesses similar vibrational features to those of NC in the 1200–1600 cm^−1^ region, assignable to stretching modes of C–N (1235 cm^−1^) and C═N (1605 cm^−1^) bonds (Supplementary Fig. 5)^34,35^. These results confirm the successful formation of the N-doped carbon framework in both samples. Raman spectroscopic analysis further demonstrates that K-SAC and NC display two distinct peaks at 1358 and 1588 cm^−1^, corresponding to the D (disorder) and G (graphite) bands of carbon, respectively (Supplementary Fig. 6). Notably, the *I*_D_/*I*_G_ intensity ratios of 0.94 for K-SAC and 0.90 for NC indicate a higher defect density and surface energy in K-SAC, which may facilitate chemical reaction kinetics.

Furthermore, X-ray absorption fine structure (XAFS) measurements were conducted to investigate the local coordination environment of K atoms^36,37^. The K K-edge X-ray absorption near-edge structure spectra of K-SAC and the KCl reference sample are displayed in Fig. 1h. Comparison with bulk KCl, the absorption edge of K-SAC is slightly shifted to lower energy, which indicates that the K single atoms in K-SAC have a distinct electronic configuration and local chemical environment relative to the K(I) ions in KCl. In addition, the Fourier-transformed extended X-ray absorption fine structure (FT-EXAFS) spectrum of K-SAC features a dominant peak at ~2.06 Å in Fig. 1i, which is assigned to K–N/O scattering. By contrast, KCl and relevant literature data exhibit prominent peaks at ~2.7 Å (K–Cl) and ~3.9 Å (K–K), and the absence of these characteristic peaks in K-SAC confirms the atomic dispersion of K species in the carbon matrix, thus excluding the presence of crystalline KCl or metallic K aggregates^34,38^. To quantitatively determine the exact coordination configuration of K atoms, least-squares fitting was performed on the EXAFS data. The optimal fitting model reveals that each K atom in K-SAC is coordinated by both N and O atoms, and specifically, the K coordination environment in K-SAC can be well simulated by a K–N path (coordination number ≈3.8, bond length 2.65 Å) and a K–O path (coordination number ≈3.6, bond length 2.72 Å) (Fig. 1j, k, Supplementary Fig. 7, and Supplementary Table 1). The formation of K–O coordination is attributed to the spontaneous adsorption of ambient O-containing species (e.g., H_2_O, CO_2_, and O_2_) onto the electropositive isolated K sites, rather than representing a well-defined intrinsic coordination shell. Accordingly, the intrinsic structural motif of the K center is identified as K–N_4_. Moreover, wavelet transform (WT) analysis was used to further elucidate the backscattering environment around K sites in Fig. 1l, m. The WT contour plot of K-SAC presents a distinct intensity maximum at ~2.6 Å^−1^ in k space, a feature characteristic of K–N/O coordination, which is clearly different from the signals of KCl and those reported in the literature with intensity maxima at 3.9 Å^−1^ for K–K and 2.7 Å^−1^ for K–Cl in k space^39^. Collectively, these XAFS analyses confirm that K species are anchored as isolated single atoms in the N-doped carbon framework.

High-resolution N 1*s* spectra of the samples were also recorded and deconvoluted into four components: pyridinic N (398.3 eV), pyrrolic N (399.1 eV), graphitic N (401.0 eV), and oxidized N (402.7 eV) (Supplementary Fig. 8). Detailed statistical analysis reveals that the pyridinic N content increases from 15.9% in NC to 19.2% in K-SAC (Supplementary Table 2). This evolution suggests that the introduction of K atoms promotes the stabilization of pyridinic N sites. This phenomenon aligns with the formation mechanism of single-atom catalysts, where metal atoms preferentially coordinate with pyridinic N at defect sites to form thermodynamically stable metal-N motifs^40–43^. Collectively, these results suggest that the K species are effectively anchored via coordination with pyridinic N, thereby defining the intrinsic atomic structure of the K-SAC.

### Enzyme-like characterizations of K-SAC

**Fig. 2:**
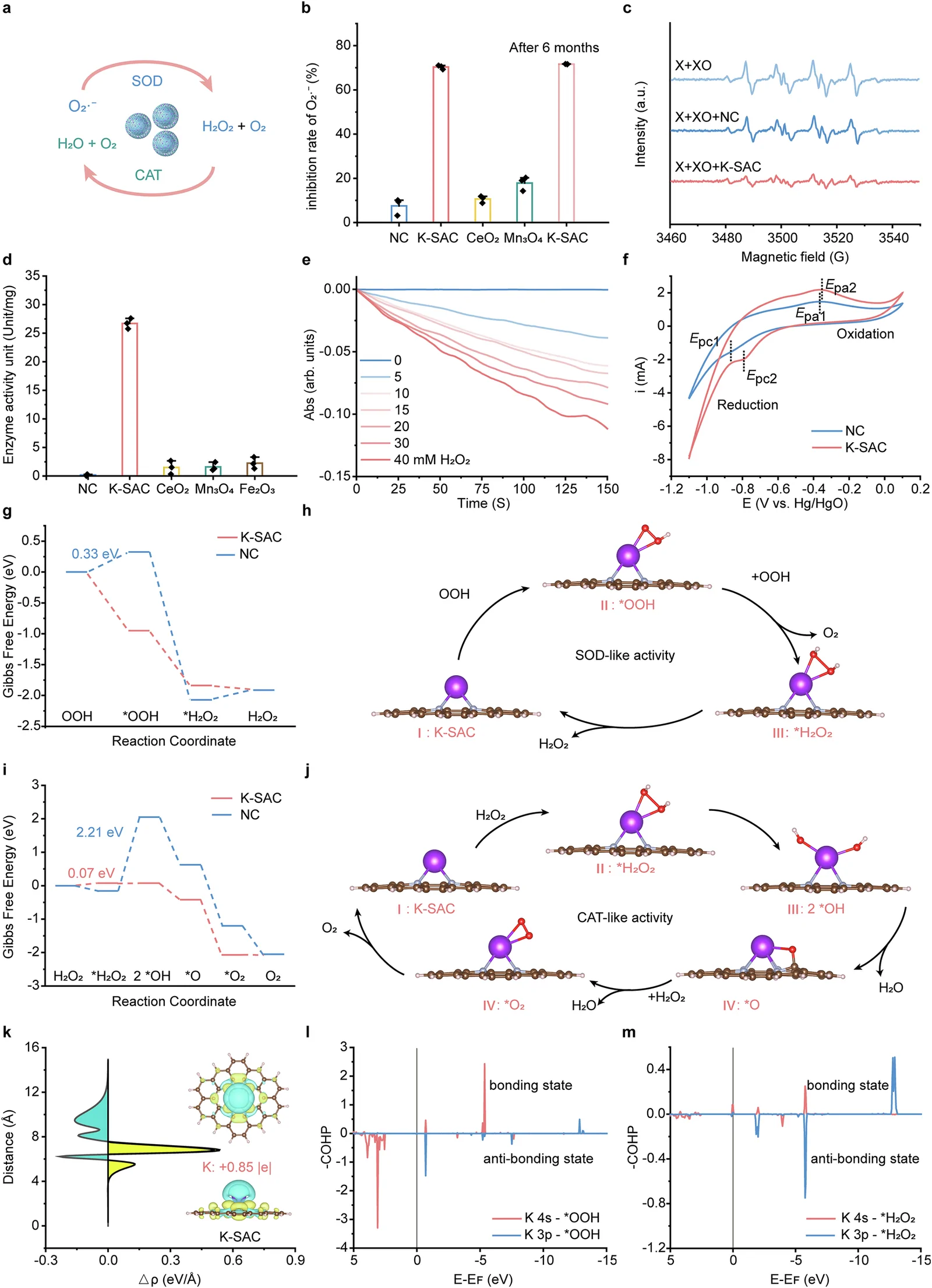
Characterization and mechanism schematics of ROS scavenging performance of K-SAC. **a** Schematic illustration of the ROS cascade clearance by K-SAC. Created by figdraw.com. **b** O_2_•^−^ inhibition rates by different catalysts (100 μg/mL, *n* = 3 tests). **c** ESR spectra analysis of the O_2_•^−^-scavenging by K-SAC and NC (X: xanthine; XO: xanthine oxidase, K-SAC: 150 μg/mL). **d** CAT-like activities of different catalysts (100 μg/mL, *n* = 3 tests). **e** CAT-like assay for the elimination of diverse concentrations of H_2_O_2_ utilizing 100 μg/mL of K-SAC using UV absorption tests. **f** CV curves of K-SAC ([K] = 10 μM) and NC (at an equivalent mass concentration as that of K-SAC). **g**–**j** Free energy profiles for the SOD- (**g**) and CAT-like (**i**) catalytic reactions by K-SAC and NC. DFT calculations for SOD- (**h**) and CAT-like (**j**) reaction pathways of K-SAC. **k** Planar-averaged electron density difference, charge density difference plots, and Bader charge analysis for K-SAC. The yellow and cyan colors indicate charge accumulation and depletion, respectively. The isosurface is taken as 0.001 e/(Bohr^3^). The structure model in (**h**–**k**) were generated from VESTA^62^. Analysis of the projected COHP for the interactions between the K 4*s*/3*p*-O in *OOH (**l**) and *H_2_O_2_ (**m**) on the K-SAC. *E*_F_ indicates the Fermi level. The results are presented as mean ± SD in (**b**, **d**). Source data are provided as a Source Data file.

ROS, including superoxide anion (O_2_•^−^) and hydrogen peroxide (H_2_O_2_), represent ubiquitous byproducts of aerobic cellular metabolism. However, excessive ROS accumulation is implicated in oxidative stress, a pathological state associated with various diseases, particularly inflammatory disorders. In endogenous enzymatic antioxidant systems, superoxide dismutase (SOD) catalyzes the conversion of O_2_•^−^ to H_2_O_2_ and O_2_. The generated H_2_O_2_ is subsequently metabolized by catalase (CAT) into H_2_O and O_2_. This sequential catalytic cascade synergistically maintains redox homeostasis, constituting a fundamental defense mechanism against oxidative stress-induced cellular damage (Fig. 2a). As such, catalytic materials emulating the activities of SOD and CAT are hypothesized to effectively alleviate oxidative stress.

Herein, the SOD-like activity of K-SAC was quantified using a WST-8 colorimetric assay. As shown in Fig. 2b and Supplementary Fig. 9, the O_2_•^−^ scavenging capacity of K-SAC exhibits a concentration-dependent response, significantly outperforming both NC and commercial anti-inflammatory catalysts (CeO_2_ and Mn_3_O_4_). Quantitative analysis reveals that K-SAC achieves a 70.31% O_2_•^−^ elimination rate at a K concentration of 2.33 μg/mL, comparable to previously reported transition metal single-atom catalysts^44–46^. Notably, K-SAC demonstrates exceptional stability, maintaining activity without significant decline after 6 months of storage under ambient conditions. Electron spin resonance (ESR) spectroscopy using 5-tert-butoxycarbonyl 5-methyl-1-pyrroline N-oxide (BMPO) as an O_2_•^−^ trapping reagent further validates the SOD-like activity. As depicted in Fig. 2c, K-SAC induces a pronounced attenuation of the characteristic O_2_•^−^ ESR signal compared to NC, which is consistent with WST-8 assay results.

Subsequently, the CAT-like activity of K-SAC was evaluated using a CAT assay kit and UV spectrophotometry. As depicted in Fig. 2d and Supplementary Fig. 10, K-SAC exhibits an enzyme activity of 26.69 U/mg, surpassing both NC and transition metal oxide nanoparticles. Kinetic studies monitoring H_2_O_2_ depletion at 240 nm reveal time- and concentration-dependent H_2_O_2_ consumption (Fig. 2e), with degradation efficiency increasing with K-SAC concentration at fixed H_2_O_2_ levels or with H_2_O_2_ concentration at fixed K-SAC doses. Lineweaver–Burk plot analysis yields a Michaelis constant (*K*_m_) of 20.379 mM and a maximum reaction velocity (*V*_m_) of 1.843 mM/min (Supplementary Fig. 11), indicating that catalytic activity and substrate affinity are comparable to those of reported transition metal-based single-atom catalysts and nanoparticles^47^.

In addition, cyclic voltammetry measurements were performed to probe the redox properties of K-SAC. As illustrated in Fig. 2f, K-SAC displays distinct electrochemical responses relative to NC, with positive shifts in both anodic (*E*_pa_) and cathodic (*E*_pc_) peak potentials. These shifts indicate that K-site incorporation elevates the redox potential of the catalyst, facilitating electron transfer reactions.

Collectively, these findings demonstrate that K-SAC possesses dual SOD- and CAT-like activities, enabling sequential scavenging of ROS through a catalytic cascade: O_2_•^−^ → H_2_O_2_ → non-toxic H_2_O and O_2_.

### Density functional theory calculations

Density functional theory (DFT) calculations were performed to unravel the catalytic mechanism underlying the antioxidant activity of K-SAC. The structural model of K-SAC, featuring a K–N_4_ coordination center embedded within a 36-carbon atom framework, was established based on EXAFS analysis (Supplementary Fig. 12).

For SOD-like activity, the possible catalytic pathways on K-SAC are illustrated in Fig. 2h. For illustrative purposes, the catalytic cycle commences with the adsorption of *OOH (the adsorbed protonated form of O_2_•^−^) onto the K active site. Subsequently, the adsorbed *OOH reacts with another OOH species, yielding *H_2_O_2_ and molecular O_2_. The cycle concludes with the desorption of dissolved H_2_O_2_. Comparative analysis for NC is provided in Supplementary Fig. 13a. Free energy profile calculations reveal that the rate-determining step (RDS) in NC exhibits an energy barrier of 0.33 eV. In contrast, K-SAC displays barrierless exothermic reactions throughout the cycle, underscoring its superior catalytic efficiency (Fig. 2g).

For CAT-like activity, the proposed reaction pathways on K-SAC are depicted in Fig. 2j. The sequence initiates with the adsorption of H_2_O_2_ onto the K site. Then, the adsorbed H_2_O_2_ decomposes into two *OH, which subsequently combine to form H_2_O and a *O species. After reacting with another H_2_O_2_, the *O species is converted to H_2_O and gaseous O_2_. Finally, O_2_ desorbs from the catalyst surface. Reaction pathways on NC, shown in Supplementary Fig. 13b, mirror those on K-SAC but with distinct energetics. Free energy analysis indicates that K-SAC exhibits a low RDS energy barrier of 0.07 eV for H_2_O_2_ adsorption, whereas NC displays an energy barrier of 2.21 eV for the RDS (the decomposition of *H_2_O_2_ into two *OH) (Fig. 2i).

Electronic structure analyses provide mechanistic insights. Planar charge density difference and Bader charge analyses reveal pronounced electron redistribution along the Z-axis between K and its four coordinated N atoms, with charge transfer from K to N (Fig. 2k). This charge polarization modelates the electronic structure of the K–N_4_ center and its interactions with adsorbates, thereby facilitating interfacial electron transfer. Crystal orbital Hamiltonian population (COHP) analysis of K-*OOH (the initial step in the SOD-like reaction) shows bonding interactions between K 4*s* orbitals and *OOH near the Fermi level. In contrast, anti-bonding interactions are observed in K 3*p*-*OOH states (Fig. 2l). Similar trends are observed in K-*H_2_O_2_ interactions (RDS in CAT-like reaction, Fig. 2m), demonstrating that K 4*s* orbitals dominate adsorbate binding, which is distinct from the d-orbital-mediated interactions in transition metal catalysts.

Collectively, these DFT calculations provide rigorous theoretical evidence for the enhanced enzyme-like activity of K-SAC, elucidating how the K–N_4_ active center facilitates efficient catalytic cycles through optimized adsorption energetics and electronic configurations.

### Intracellular uptake, anti-ALI effects, and biocompatibility of K-SAC

**Fig. 3:**
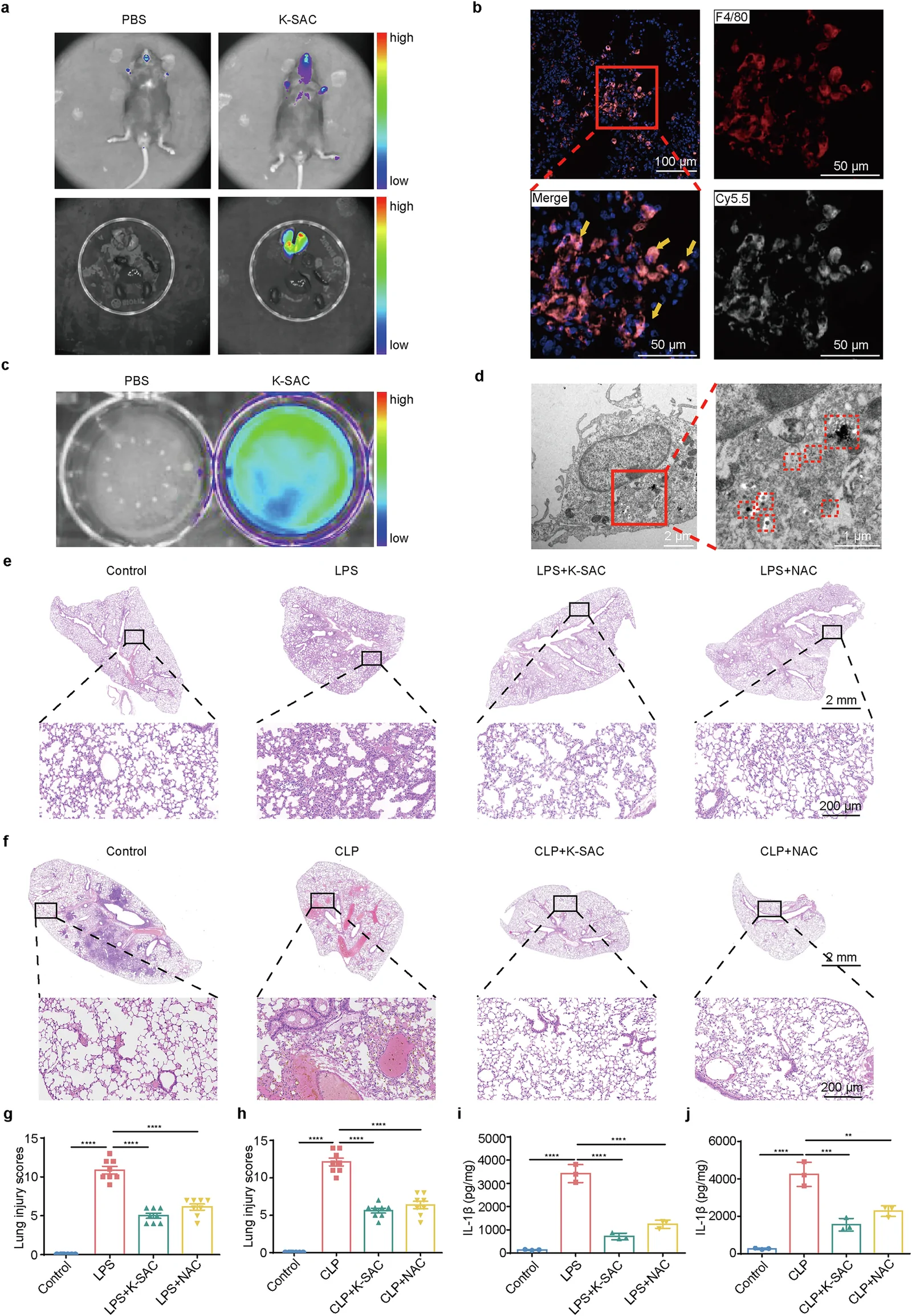
Biocompatibility, intracellular uptake, and anti-ALI effects of K-SAC. **a** Aerosol inhalation of Cy5.5-labeled K-SAC (1 mg/kg) in mice: the whole-body fluorescence image at 24 h (upper) and ex vivo organ fluorescence analysis (lower) via IVIS. **b** Immunofluorescence co-staining images for macrophages (F4/80) and Cy5.5 in mouse lung tissues. **c** Transwell co-incubation of BMDMs with Cy5.5-labeled catalysts for 24 h: representative fluorescence images of catalyst migration to lower wells. **d** TEM images of BMDMs showing phagocytosis of K-SAC in 24 h post-co-incubation (red dotted boxes show the K-SAC). The data shown are representative of three independent experiments with similar results. Representative H&E staining images of mouse lung tissues in LPS- (**e**) or CLP-induced (**f**) ALI mice treated with K-SAC (1 mg/kg, aerosol inhalation) or NAC (360 mg/kg, aerosol inhalation). Quantitative lung injury scores in LPS (**g**) and CLP (**h**) models. IL-1β expression in lung tissues of LPS- (**i**) and CLP-induced (**j**) ALI with K-SAC or NAC treatment. The results are presented as mean ± SD with *n* = 8 (**g**, **h**) and *n* = 3 (**i**, **j**) independent samples for each group. Statistical significance was calculated via one-way ANOVA and Tukey’s multiple comparisons (**g**–**j**). Source data are provided as a Source Data file.

We systematically evaluated the biomedical potential of K-SAC by examining its cellular uptake, therapeutic efficacy, and safety profile using both in vitro and in vivo models. Following nasal administration of Cy5.5-labeled K-SAC, strong fluorescence signals are detected in lung tissues (Fig. 3a). Immunofluorescence staining demonstrates co-localization of the macrophage marker F4/80 with Cy5.5 signals (Fig. 3b and Supplementary Fig. 14), confirming efficient macrophage uptake. This finding is further supported by in vitro studies with bone marrow-derived macrophages (BMDMs) and peritoneal macrophages (PMs). After 24-h incubation with Cy5.5-labeled K-SAC in transwell systems, fluorescent particles (K-SAC) are detected in the lower compartment  (Fig. 3c), and TEM images of BMDMs (Fig. 3d) and PMs (Supplementary Figs. 15 and 16) visualize intracellular accumulation of K-SAC, confirming preferential macrophage uptake. This preferential uptake is primarily driven by a passive targeting mechanism. The specific physical properties of K-SAC, including its hydrodynamic diameter of ~200 nm and abundant mesoporous structure, synergize with the inherently high phagocytic capacity of activated alveolar macrophages in the inflammatory microenvironment to facilitate this efficient internalization.

To evaluate the biosafety and therapeutic potential of K-SAC, mice were administered K-SAC via aerosol inhalation at doses of 1, 2.5, 5, and 10 mg/kg three times within 48 h. H&E staining revealed no obvious toxicological manifestations following nasal administration (Supplementary Fig. 17a, b). We next examined the therapeutic efficacy of K-SAC at lower doses (0.1, 1, and 2.5 mg/kg), and 1 mg/kg K-SAC exhibited comparable protective effects relative to 2.5 mg/kg (Supplementary Fig. 17c, d). Histopathological examination of major organs and analysis of serum biomarkers (ALT, AST, creatinine, and urea) detected no signs of toxicity following nasal administration (Supplementary Figs. 18 and 19). Comprehensive safety evaluations revealed excellent biocompatibility. Cell viability assays showed no significant cytotoxicity at concentrations up to 10 μg/mL (Supplementary Fig. 20). These results collectively demonstrate the favorable safety profile of K-SAC for potential clinical application. Furthermore, we compared the therapeutic outcomes of prophylactic and post-challenge administration of K-SAC, and found that pre-administration conferred superior protection against acute lung injury (Supplementary Fig. 17e, f).

The therapeutic potential of K-SAC was assessed in two established ALI models induced by lipopolysaccharide (LPS) and cecal ligation and puncture (CLP), respectively. Histological examination shows that K-SAC treatment substantially attenuates pathological changes, including alveolar wall thickening and inflammatory cell infiltration (Fig. 3e, f). Quantitative analysis confirms significant improvement in lung injury scores (Fig. 3g, h), accompanied by reduced IL-1β levels (Fig. 3i, j), indicating potent anti-inflammatory activity, which is comparable to N-acetylcysteine (NAC) treatment. To investigate the role of macrophages in the therapeutic effect of K-SAC against ALI, we depleted macrophages in lung tissue using clodronate liposomes and examined whether the therapeutic efficacy of K-SAC was altered. We found that the therapeutic effect of K-SAC on lung injury was markedly attenuated following macrophage depletion (Supplementary Fig. 21a, b), demonstrating that macrophages are essential for K-SAC to ameliorate acute lung injury. Although neutrophils were also significantly decreased after K-SAC treatment (Supplementary Fig. 21c, d), given that our experimental data confirmed K-SAC was predominantly enriched in macrophages, we speculate that this change may be attributed to reduced chemokine secretion following the resolution of inflammation by macrophages.

### K-SAC alleviates gasdermin D (GSDMD)-mediated pyroptosis in macrophages

**Fig. 4:**
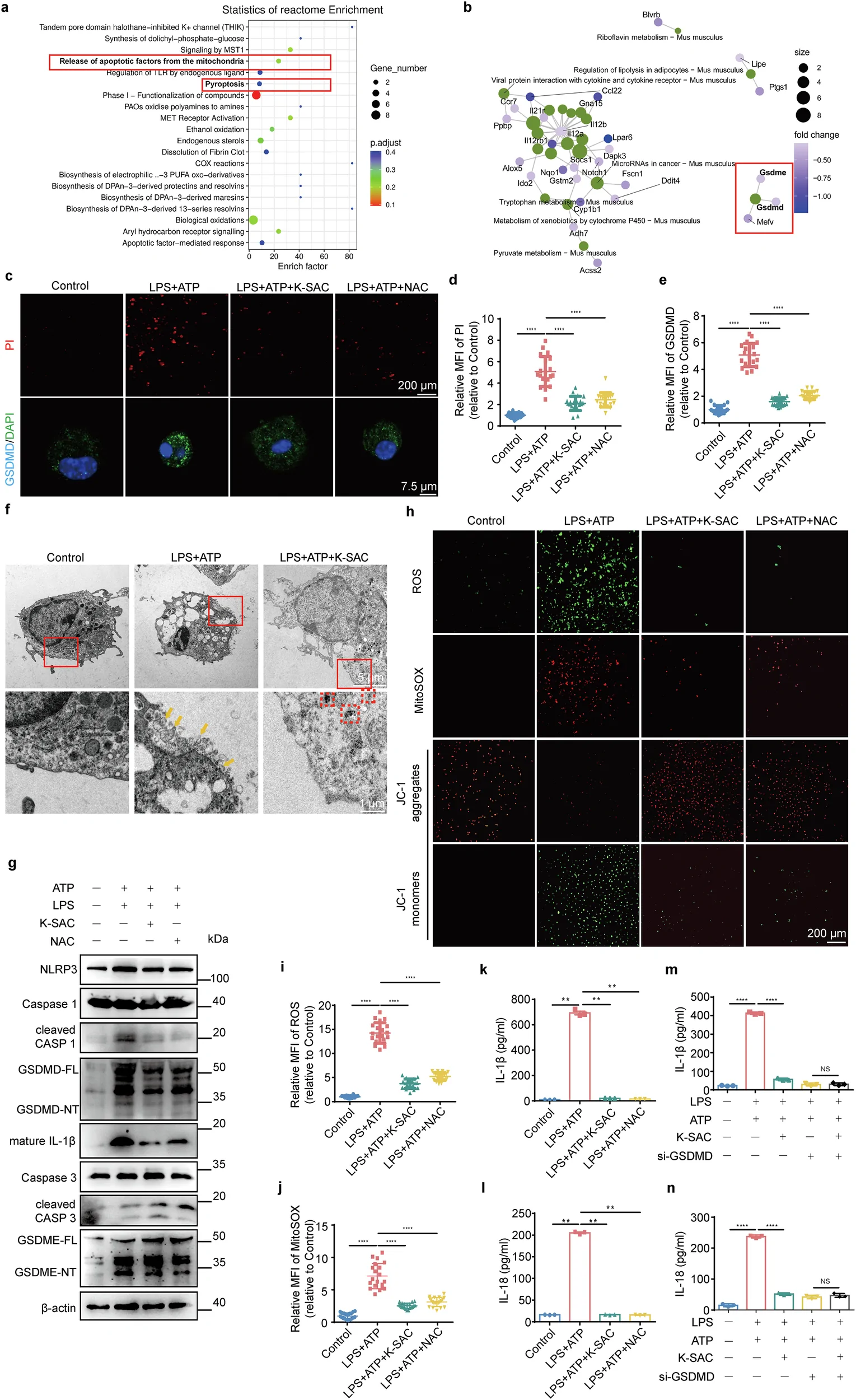
K-SAC alleviates GSDMD-mediated pyroptosis and mitochondrial damage in macrophages. Reactome (**a**) and KEGG (**b**) enrichment analyses revealing the significantly inhibition of pyroptosis-related pathways by K-SAC in LPS+ATP-stimulated BMDMs. **c**–**e** Representative PI immunofluorescent staining (**c**, upper panel) and GSDMD immunofluorescent staining (**c**, lower panel) images in LPS and ATP-stimulated BMDMs with K-SAC or NAC treatment (scale bar: 200 μm, upper panel; 7.5 μm, lower panel). The MFI quantifications of PI (**d**) and GSDMD (**e**) (*n* = 20 cells per group). **f** TEM images of BMDMs: yellow arrows indicate damaged membranes and pyroptotic bodies after LPS and ATP stimulation (scale bar: upper, 5 μm; lower, 1 μm). The data shown are representative of three independent experiments with similar results. **g** Western blot analyses of NLRP3, caspase-1, cleaved caspase-1, GSDMD, mature IL-1β, Caspase-3, cleaved Caspase-3, GSDME, and β-actin in BMDMs. The data shown are representative of three independent experiments with similar results. **h** Fluorescence images of ROS, mitoSOX, and JC-1 staining in BMDMs. MFI values of ROS (**i**) and mitoSOX (**j**) (*n* = 20 cells per group). IL-1β (**k**) and IL-18 (**l**) levels in BMDMs supernatants. IL-1β (**m**) and IL-18 (**n**) levels in BMDMs supernatants treated with K-SAC, LPS, and ATP, with or without GSDMD siRNA intervention. The results are presented as mean ± SD with *n* = 20 (**d**, **e, i**, **j**), *n* = 3 (**k**–**n**) independent samples for each group. Statistical significance was calculated via one-way ANOVA, Tukey’s multiple comparisons (**d**, **e**, **i**–**n**). Source data are provided as a Source Data file.

To investigate the anti-inflammatory mechanism of K-SAC, we performed RNA sequencing on LPS-stimulated BMDMs with or without K-SAC treatment. The analysis revealed significant downregulation of pyroptosis-related genes (Fig. 4a, b), suggesting K-SAC may alleviate pulmonary inflammation by inhibiting macrophage pyroptosis.

We established an in vitro pyroptosis model using LPS-primed BMDMs and PMs followed by ATP stimulation^48^. Compared to the positive control NAC (a known pyroptosis inhibitor)^44^, K-SAC showed significantly better protection against LPS- and ATP-induced cytotoxicity (Supplementary Fig. 22). PI staining confirmed both K-SAC and NAC treatments effectively reduced macrophage cell death (Fig. 4c, d, and Supplementary Fig. 23).

Further analysis identified GSDMD and GSDME, key mediators of pyroptosis, as being significantly downregulated after K-SAC treatment (Fig. 4b). Consistently, NAC (as a positive control) also reduced GSDMD and GSDME expression (Supplementary Fig. 24). Confocal microscopy demonstrated K-SAC attenuates the increase in GSDMD fluorescence intensity after LPS and ATP stimulation (Fig. 4c, e, and Supplementary Fig. 25). Given that pyroptotic pore formation is critical for cytokine release^49^, TEM was used to reveal that K-SAC prevents mitochondrial disruption and pyroptotic body extrusion after LPS and ATP stimulation (Fig. 4f and Supplementary Fig. 26).

Mechanistically, K-SAC specifically decreased full-length GSDMD (GSDMD-FL) and its cleaved N-terminal fragment (GSDMD-NT), without affecting GSDME-FL or GSDME-NT expression (Fig. 4g and Supplementary Fig. 27). Furthermore, K-SAC inhibits caspase-1 activation (required for GSDMD cleavage) but not caspase-3 activation (which activates GSDME). These results demonstrate that K-SAC exerts its anti-inflammatory effects primarily through selective inhibition of the GSDMD-mediated pyroptotic pathway in macrophages.

### ROS-scavenging and anti-inflammatory effects in vitro

Our previous work revealed significant differences in mitochondria-related pathways between ARDS patient subgroups, highlighting the importance of mitochondrial function in disease outcomes^50^. TEM examination of BMDMs and PMs shows distinct mitochondrial changes following LPS and ATP stimulation (Supplementary Fig. 28a, b). While control cells display normal mitochondrial morphology with intact cristae, treated cells exhibit shrinkage, rounding, and structural damage (Supplementary Fig. 28c–f).

Since mitochondria represent the primary intracellular source of ROS and mitochondrial ROS (mtROS) is central to inflammasome activation and pyroptosis^51–53^, we evaluated K-SAC’ antioxidant capacity. Consistent with known connections between oxidative stress and lung injury, K-SAC effectively reduces LPS- and ATP-induced ROS accumulation, outperforming NAC treatment (Fig. 4h, i, and Supplementary Fig. 29a, b). MitoSOX staining confirms K-SAC’ ability to suppress mtROS production (Fig. 4h, j, and Supplementary Fig. 29a, c). JC-1 assays demonstrate that K-SAC better preserved mitochondrial membrane potential compared to NAC (Fig. 4h and Supplementary Fig. 29a), indicating superior mitochondrial protection. Collectively, these results demonstrate the potent ROS-scavenging activity of K-SAC, providing a mechanistic basis for its inhibition of macrophage pyroptosis.

The anti-inflammatory effects paralleled these antioxidant properties. K-SAC significantly decreases IL-1β and IL-18 release in supernatants of LPS- and ATP-stimulated BMDMs and PMs (Fig. 4k, l, and Supplementary Fig. 30). We detected K-SAC’s effects on pyroptosis in macrophages with or without GSDMD siRNA intervention (Fig. 4m, n, and Supplementary Fig. 31) and in macrophages treated with disulfiram (Supplementary Fig. 32). We found that K-SAC’s protective effect is similar to that of disulfiram, and is GSDMD-dependent. These findings position K-SAC as a dual-action therapeutic agent that simultaneously neutralizes ROS and attenuates inflammation, offering promising potential for treating inflammatory lung diseases.

### K-SAC promotes ESCRT-dependent membrane repair and membrane phospholipid remodeling to inhibit pyroptosis

Plasma membrane rupture is a hallmark of pyroptosis^54^. LPS-induced pyroptosis causes membrane permeabilization, allowing excessive calcium influx^55^. GSDMD pores form in both plasma and mitochondrial membranes, contributing to organelle dysfunction^56,57^. Importantly, GSDMD-deficient cells resist membrane damage, confirming the critical role of GSDMD in membrane integrity^18,58^.

**Fig. 5:**
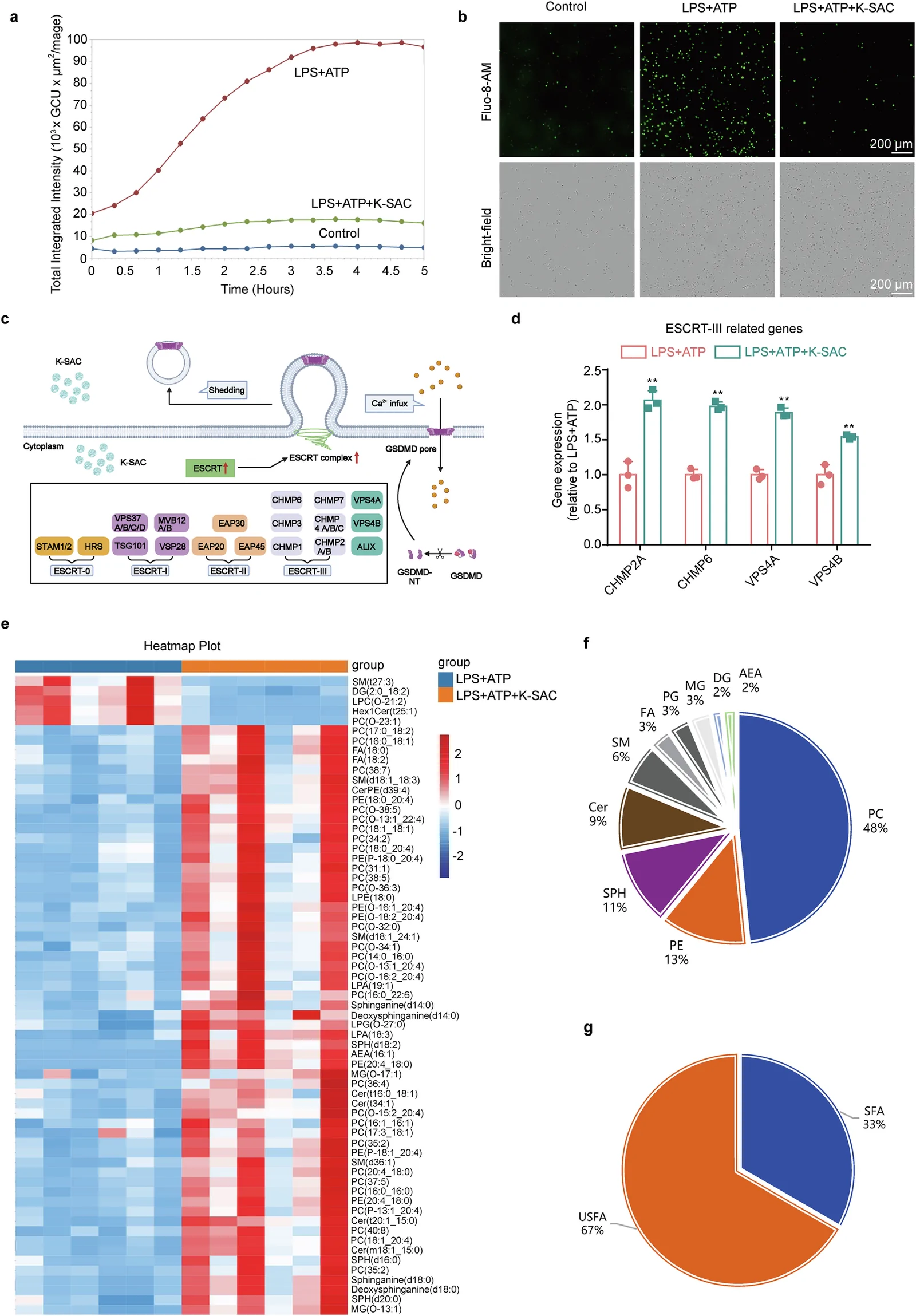
K-SAC promotes ESCRT-dependent membrane repair and membrane phospholipid remodeling to inhibit macrophage pyroptosis. **a** Total integrated intensity of Fluo-8-AM in cells after 5 h of LPS and ATP stimulation. **b** Representative immunofluorescent images of Fluo-8-AM in cells stimulated with LPS and ATP for 5 h (upper) and corresponding bright-field images (lower) (scale bar: 200  μm). The data shown are representative of three independent experiments with similar results. **c** Schematic diagram of the ESCRT system in cells. Created in BioRender. Yu, W. (2026) https://BioRender.com/9t2sky0. **d** Expression changes of key ESCRT system genes after K-SAC treatment. **e** Heatmap plot of differentially expressed lipids from lipidomics analysis after K-SAC treatment. **f**, **g** Classification of differential lipids between the LPS group and LPS+K-SAC group as shown in (**e**). Lipids were classified by lipid class, including PC, PE, SPH, and Cer, etc. (**f**) and also categorized into saturated fatty acids and unsaturated fatty acids based on the presence or absence of unsaturated double bonds (**g**). The results are presented as mean ± SD with *n* = 3 (**d**) independent samples for each group. Statistical significance was calculated via unpaired t-test, two-tailed (**d**). Source data are provided as a Source Data file.

Our experiments show K-SAC significantly reduces intracellular calcium levels (Fig. 5a, b, and Supplementary Fig. 33), indicating preserved membrane integrity. TEM images confirm K-SAC prevents the severe membrane disruption seen in pyroptotic cells (Fig. 4f and Supplementary Fig. 26) and protects mitochondrial structure (Supplementary Fig. 28). Reduced PI uptake in K-SAC-treated cells further demonstrates membrane stabilization (Fig. 4c, d, and Supplementary Fig. 23).

Cells employ the ESCRT system to repair limited GSDMD pore formation during early pyroptosis^59^. Previous reports indicate that ESCRT-mediated membrane repair represents a critical endogenous mechanism for regulating GSDMD pores^55^. We found K-SAC upregulates essential ESCRT-III components (CHMP2A, CHMP6) and their regulator VPS4A/B (Fig. 5c, d, and Supplementary Fig. 34), suggesting enhanced membrane repair capacity.

Membrane lipid composition significantly influences GSDMD pore dynamics. GSDMD-NT exhibits high affinity for mitochondrial cardiolipin but does not interact with phosphatidylcholine (PC) and phosphatidylethanolamine (PE), major plasma membrane phospholipids^60^. Lipidomic analysis revealed K-SAC alters phospholipid profiles in pyroptotic BMDMs (Supplementary Fig. 35). Among these altered phospholipids, the differential ones (Fig. 5e and Supplementary Data 1) primarily comprised membrane-structural phospholipids, such as PC, PE, and sphingomyelin (SM) (Fig. 5f). Particularly, K-SAC increases unsaturated fatty acid (USFAs)-containing phospholipids compared to untreated pyroptotic BMDMs (Fig. 5g). These changes in PC, PE, and SM likely contribute to membrane stabilization and repair.

### Anti-pyroptosis and anti-inflammatory efficacy of K-SAC in vivo

Having established the anti-inflammatory effects of K-SAC in vitro, we next examined the therapeutic potential in mouse models of ALI. In LPS- and CLP-induced ALI, pathological changes and the initial ROS burst develop rapidly. To efficiently scavenge early oxidative stress and assess early blockade of the pyroptotic cascade, we used prophylactic and co-treatment administration. This strategy ensures sufficient local bioavailability of K-SAC in the alveolar microenvironment before irreversible severe tissue damage occurs.

**Fig. 6:**
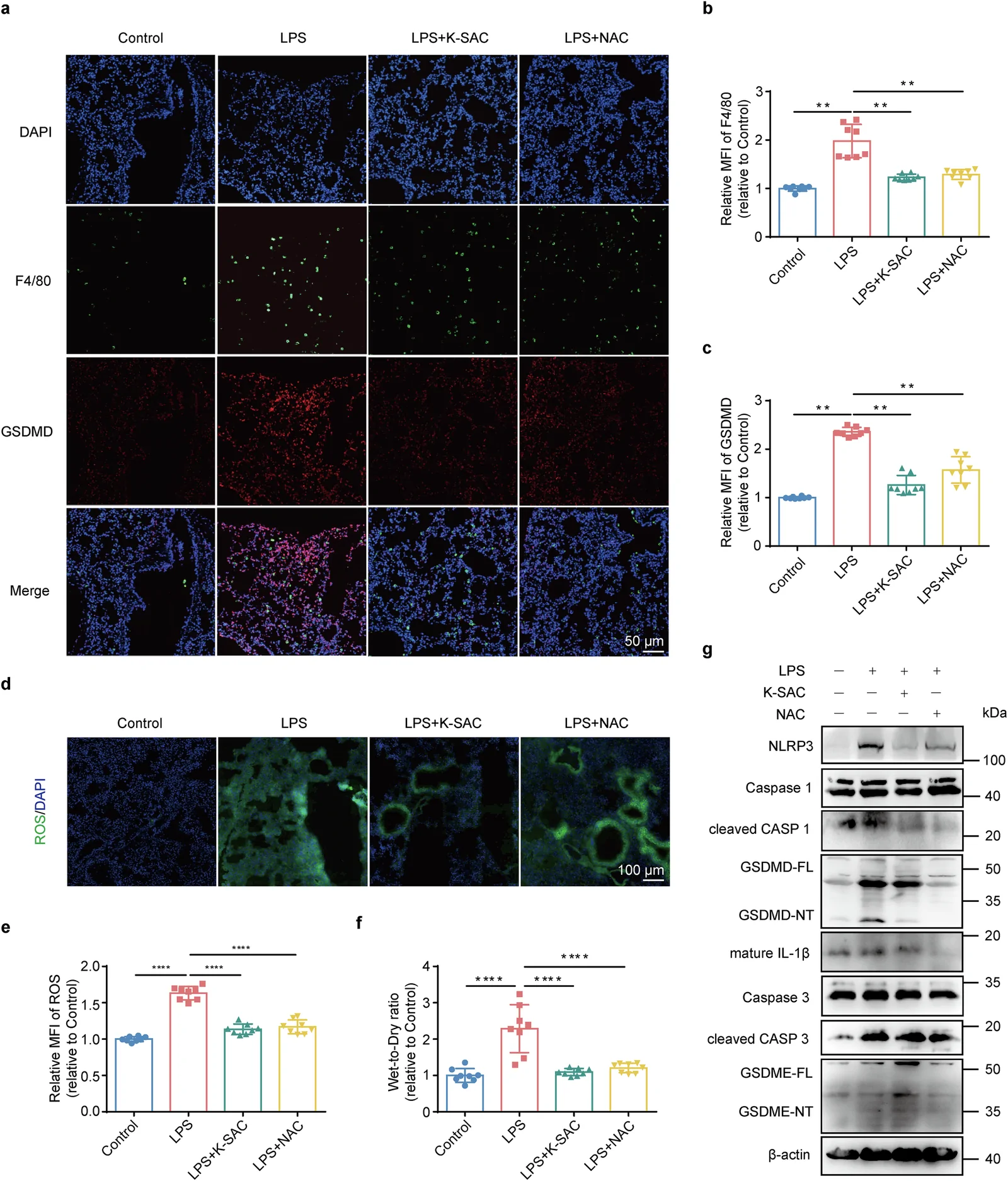
K-SAC alleviates LPS-induced ALI via inhibiting macrophage pyroptosis. Representative F4/80 and GSDMD immunofluorescent staining in lung sections (**a**) with quantitative analysis (**b**, **c**). Representative ROS staining images in lung sections (**d**) with quantitative analysis (**e**). **f** Lung wet-to-dry (W/D) weight ratio assessment. **g** Western blot analyses of NLRP3, caspase-1, cleaved caspase-1, GSDMD, mature IL-1β, caspase-3, cleaved caspase-3, GSDME, and β-actin in lung tissues. The data shown are representative of three independent experiments with similar results. The results are presented as mean ± SD with *n* = 8 (**b**,** c**, **e**, **f**) independent samples for each group. Statistical significance was calculated via one-way ANOVA and Tukey’s multiple comparisons (**b**, **c**,** e**, **f**). Source data are provided as a Source Data file.

Immunohistochemical analysis shows that inhaled K-SAC significantly reduces alveolar macrophage accumulation in LPS-induced ALI lungs (Fig. 6a, b, and Supplementary Fig. 36a, b). Immunofluorescence staining reveals that K-SAC treatment effectively suppresses LPS-induced GSDMD upregulation (Fig. 6a, c, and Supplementary Fig. 36a, c). The co-localization of F4/80 and GSDMD confirms that K-SAC alleviates ALI by inhibiting macrophage pyroptosis. Consistent with its antioxidant properties, K-SAC substantially decreased pulmonary ROS levels in LPS-challenged mice, showing better efficacy compared to NAC (Fig. 6d, e, and Supplementary Fig. 37). K-SAC also improves alveolar-capillary barrier function, as evidenced by reduced lung wet-to-dry weight ratios (Fig. 6f and Supplementary Fig. 38). Western blot analysis demonstrates that K-SAC more effectively downregulates pyroptosis-related proteins than NAC (Fig. 6g and Supplementary Fig. 39). These results collectively demonstrate that K-SAC effectively ameliorates LPS-induced and sepsis-associated ALI by attenuating inflammatory responses and oxidative stress.

## Discussion

**Fig. 7:**
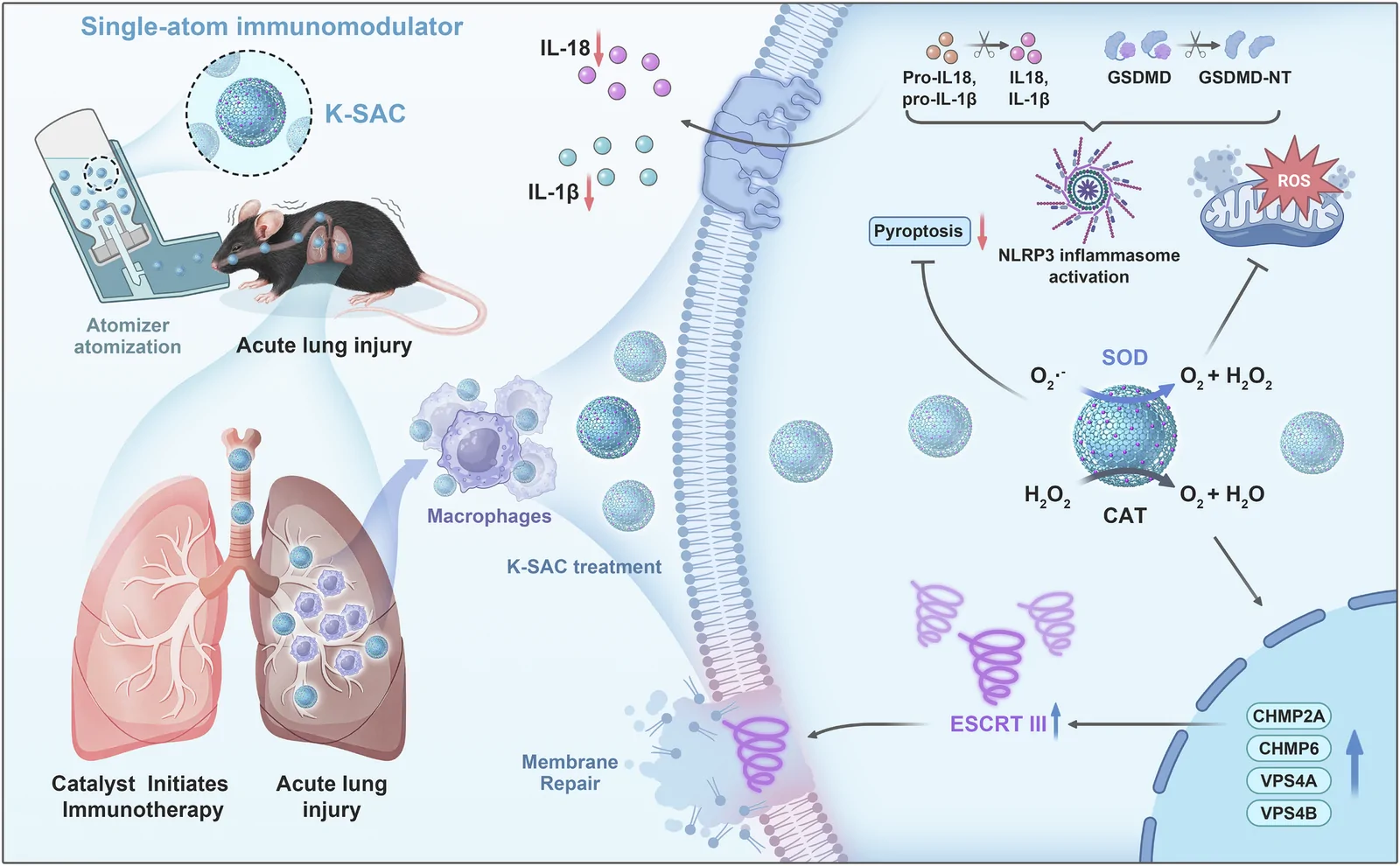
Schematic illustration of the mechanism of the K-SAC immunomodulator in the treatment of acute lung injury. The as-fabricated K-SAC single-atom immunomodulator possesses dual SOD- and CAT-mimicking catalytic activities to eliminate ROS. By removing excess ROS, K-SAC preserves mitochondrial integrity and blocks ROS-triggered NLRP3 inflammasome activation, thereby restraining GSDMD-dependent pyroptosis and the secretion of pro-inflammatory IL-1β and IL-18. Meanwhile, K-SAC activates ESCRT III-associated proteins to facilitate membrane phospholipid remodeling. Through the synergistic effects of pyroptosis inhibition and membrane restoration, inhaled K-SAC efficiently attenuates inflammatory cascades and oxidative stress in ALI.

In this study, we have developed a potassium-based single-atom catalyst by making use of potassium (K), the most abundant intracellular metal in human bodies, and demonstrated that K-SAC can (1) effectively scavenge ROS and suppress ROS-induced inflammatory responses by mimicking the catalytic activities of SOD and CAT; (2) inhibit GSDMD-mediated pyroptosis, and meanwhile activate the ESCRT system and membrane phospholipid remodeling to mitigate macrophage pyroptosis; (3) effectively protect mitochondrial integrity; (4) effectively attenuate inflammatory cascades and oxidative stress in ALI with excellent biocompatibility. Notably, K-SAC alleviates both LPS- and sepsis-induced ALIs, two typical conditions characterized by immune dysregulation (Fig. 7). More broadly, this work establishes s-block metal single-atom catalysts as a class of antioxidant catalytic materials and provides a basis for harnessing physiologically abundant elements, such as K, Na, Ca, and Mg, in biomedical catalyst design.

Furthermore, the inherent heterogeneity of macrophage populations presents a critical avenue for future precision medicine. Because monocyte-derived macrophages (MoMs) function as the primary drivers of early-stage, pyroptosis-prone inflammation, future material optimization should focus on functionalizing K-SAC to selectively target these specific infiltrating subsets. Tailoring nanomaterial targeting capabilities based on the dynamic spatiotemporal distribution of these subsets will pave the way for more precise immunotherapeutic interventions. Beyond macrophages, navigating the broader pulmonary immune network, particularly the involvement of neutrophils, will be a key focus of our future investigations. Neutrophils play a dual role in lung injury, functioning in pathogen surveillance while also driving pulmonary microcirculatory dysfunction via capillary occlusion. While our experimental data demonstrated a marked decrease in pulmonary neutrophil accumulation following K-SAC administration, the strict in vivo co-localization of inhaled K-SAC exclusively with macrophages led us to center our current mechanistic exploration on macrophage modulation.

Despite these promising acute outcomes, advancing K-SAC toward clinical translation necessitates addressing long-term safety and practical logistical hurdles. Future studies must establish extended safety profiles spanning 2–4 weeks to systematically evaluate chronic toxicity, tissue accumulation, clearance kinetics, and the safety of repeated dosing regimens. Bridging to human trials will further demand scalable manufacturing, optimized administration routes for intensive care (e.g., transitioning to aerosolized nebulization for ventilator compatibility), and navigating rigorous regulatory pathways. Ultimately, overcoming these translational challenges will help fully realize K-SAC as a versatile immunomodulatory platform, highlighting its tremendous potential in treating a broad range of inflammation-driven diseases, such as COVID-19, systemic sepsis, and inflammatory bowel disease (IBD).

## Methods

### Ethical statement

All the animal experiments involved in this work were approved by the Scientific Investigation Board of Shanghai Pulmonary Hospital (Shanghai, China) (approval no. K24-625).

### Synthesis of K-SAC

A homogeneous solution was prepared by combining 40 mL of ethanol with 60 mL of distilled water (DIW). Tris (0.6 g) was dissolved in the resulting mixture under continuous stirring for 20 min. Subsequently, potassium chloride (0.33 g) and dopamine hydrochloride (0.1 g) were sequentially added to the Tris-containing solution, followed by additional stirring for 20 h. The precipitates were isolated via centrifugation, washed extensively with ethanol and DIW, and uniformly dispersed in 10 mL of DIW using ultrasonication. The dispersed suspension was lyophilized for 48 h to obtain a solid product. Finally, pyrolysis was conducted at 950 °C for 2 h under N_2_ atmosphere, with a controlled heating rate of 5 °C/min. The resulting potassium single-atom catalyst (K-SAC) was collected after cooling to ambient temperature.

### Cy5.5 labelling of K-SAC

Five milligrams of K-SAC was dispersed in 5 mL dimethyl sulfoxide (DMSO) containing Sulfo-Cy5.5-COOH (1 mg/mL) and sonicated for 1 h. The mixture was then dialyzed against DIW using a 1 kDa molecular weight cutoff membrane to remove unbound dye molecules. The resulting product was lyophilized for 48 h to yield Cy5.5-conjugated K-SAC.

### Cell culture

Peritoneal macrophages (PMs) were harvested from mice 72 h after thioglycollate (BD, Sparks, MD) injection. BMDMs were generated from bone marrow cells stimulated with recombinant mouse M-CSF (20 ng/mL). All cells were cultured in DMEM (Gibco) containing 10% FBS (Gibco) and 1% penicillin/streptomycin in a 5% CO_2_ atmosphere at 37 °C.

### SOD-like activity

The ability of K-SAC to clear O_2_•^−^ was verified using ESR measurements. In a typical experimental setup, K-SAC or NC (150 μg/mL) was introduced into PBS solution (pH 7.4) containing 25 mM BMPO, 1 mM xanthine, and 0.0125 U/mL xanthine oxidase. Subsequently, the ESR spectra of the reaction mixtures were recorded.

The SOD-like activities of NC, K-SAC, Mn_3_O_4_, and CeO_2_ were verified by a total SOD assay kit (WST-8) according to the instructions. Specifically, O_2_•^−^ was produced by the reaction of xanthine with xanthine oxidase, and SOD was able to catalyze O_2_•^−^ elimination. Based on the characteristic absorption of formazan dye at 450 nm formed by the reaction of O_2_•^−^ with WST-8, the inhibition rate of samples could be identified by colorimetric analysis using the WST-8 probe. First, various samples at different concentrations (12.5, 25, 50, 100, and 150 μg/mL) were mixed with xanthine, xanthine oxidase, and WST-8. Then, the absorbance change at 450 nm was recorded continuously during the 30 min of incubation. Finally, the inhibition percentage was calculated based on the following equation:

1 $${{\mathrm{inhibition}}}(\%)=\frac{{A}_{0}-A}{{A}_{0}}\times 100\%$$

where *A* is the absorbance of various samples, and *A*_*0*_ is that of the control samples.

### CAT-like activity

The CAT-like activities of NC, K-SAC, Fe_2_O_3_, Mn_3_O_4_, and CeO_2_ were assessed by a Catalase Assay Kit according to the instructions. The absorbance change at 450 nm was recorded to calculate the CAT-like activity. Subsequently, kinetic studies were carried out by varying the H_2_O_2_ concentrations or the K-SAC concentrations. Specifically, different concentrations of K-SAC (0, 25, 50, 100, and 200 μg/mL) were mixed with 10 mM of H_2_O_2_ in PBS buffer (pH 7.4), and different concentrations of H_2_O_2_ (0, 5, 10, 15, 20, 30, and 40 mM) were mixed with 100 μg/mL of K-SAC in PBS buffer (pH 7.4). Then, the decline in absorbance of H_2_O_2_ at 240 nm was collected using a UV–Vis spectrophotometer, and the Michaelis constant (*K*_m_) and maximal reaction velocity (*V*_max_) were determined using the following equation:

2 $$V=\frac{{V}_{\max }\times [S]}{{K}_{m}+[S]}$$

where *v* is the initial velocity, *V*_max_ is the maximal reaction velocity, [*S*] is the concentration of substrate, and *K*_m_ is the Michaelis constant.

### Animal studies

Male C57BL/6 mice (8 weeks old) were obtained from Joint Ventures Sipper BK Experimental Animals (Shanghai, China) and maintained under specific-pathogen-free (SPF) conditions at 22 ± 1 °C with 50 ± 10% humidity and a 12-h light/dark cycle. Male mice were exclusively utilized in the current study to eliminate the confounding effects of estrous cycle-driven hormonal fluctuations on baseline immune responses and to reduce intra-group variability in the severity of lung inflammation. All procedures followed the NIH Guide for the Care and Use of Laboratory Animals (Publication No. 8023, revised 1978) with approval of the Scientific Investigation Board of Shanghai Pulmonary Hospital (Shanghai, China).

### ALI mouse models and treatment

For LPS-induced acute lung injury, 10 mg/kg LPS was injected intraperitoneally. Mice were administered K-SAC (1 mg/kg in PBS) or NAC (360 mg/kg in PBS) at 48 h before, 24 h before, and concurrently with LPS challenge. The sepsis-induced ALI model was induced by cecal ligation and puncture (CLP) as standardized previously^61^. Mice were anesthetized with 2,2,2-tribromoethanol (400 mg/kg, i.p.). After a midline abdominal incision, the cecum was exposed, and 50% of the distal segment was ligated with 4-0 silk suture without blocking the ileocecal valve. A single through-and-through puncture was made with a 21-gauge needle, and a small volume of feces was gently extruded. The cecum was repositioned, and the incision was closed layer-by-layer. Sham-operated mice underwent the same procedure without ligation and puncture. All animals received subcutaneous fluid resuscitation (prewarmed saline, 5 mL/100 g) postoperatively. After LPS and CLP modeling, mice were monitored every 6 h for 24 h by trained laboratory personnel for vital signs, locomotor activity, food and water intake, and wound healing. At 6 h after LPS-induced ALI modeling and 24 h after CLP-induced ALI modeling, mice were euthanized via intraperitoneal injection of an overdose of sodium pentobarbital (200 mg/kg). Successful euthanasia was verified by complete cessation of breathing, loss of corneal reflexes, and absence of cardiac activity prior to tissue collection.

### In vivo and in vitro imaging

The images of the in vivo study and in vitro study were obtained on an IVIS (Bio-Real QuikView 3000) and the supporting software. For in vivo imaging, Cy5.5-labeled K-SAC (1 mg/kg) was administered via aerosol inhalation to LPS-challenged mice. After 24 h, whole-body fluorescence was captured using an IVIS, followed by ex vivo organ imaging. For in vitro studies, PMs and BMDMs were cultured in transwells, treated with Cy5.5-K-SAC (1 μg/mL) for 18 h, then stimulated with LPS (1 μg/mL) for 6 h before imaging.

### Western blotting

Cells/tissues were lysed (Cell Signaling Technology, #9806) with protease inhibitors (Roche, #04693132001). Lysates were centrifuged (12,000 × *g*, 15 min, 4 °C), and protein concentrations were determined by BCA assay (Pierce, #23225). Equal protein amounts were separated by SDS-PAGE for immunoblotting. The primary antibodies used in this study were as follows: NLRP3 (1:1000, Immunoway, China), caspase 1 (1:1000, Immunoway, China), cleaved caspase 1 (1:1000, Affinity, China), GSDMD (1:1000, Abcam, USA), GSDME (1:1000, CST, USA), IL-1β (1:1000, Immunoway, China), caspase 3 (1:1000, CST, USA), cleaved caspase 3 (1:1000, CST, USA), β-actin (1:1000, Proteintech, USA).

### Quantitative real-time polymerase chain reaction (RT-qPCR)

Total RNA was extracted with TRIzol, reverse transcribed using M-MLV Reverse Transcriptase (Takara Biomedical Technology (Beijing) Co., Ltd., Beijing, China), and amplified with SYBR Premix ExTaq (Takara Biomedical Technology (Beijing) Co., Ltd., Beijing, China) on an ABI QuantStudio 6 Flex. β-actin served as the reference gene, with relative expression calculated via the 2^−ΔΔCt^ method. cDNA amplification was performed on the ABI-QuantStudio 6 Flex. Primer sequences are listed in Supplementary Table 3.

### Enzyme-linked immunosorbent assay (ELISA)

IL-1β and IL-18 levels in supernatants and lung homogenates were quantified using commercial kits (Liankebio, Hangzhou, China), respectively, according to the manufacturer’s instructions.

### Immunofluorescence

For immunofluorescent staining of GSDMD, F4/80, and DAPI, BMDMs and PMs were cultured in 6-well plates (2 mL per well). After rinsing twice with PBS, cells were fixed with 4% paraformaldehyde (ServiceBio, Wuhan, China) for 15 min at room temperature. Permeabilization was performed using 0.5% Triton X-100 in PBS for 10 min, followed by blocking with 5% BSA (Gibco, Australia Origin) in PBS for 1 h, and then were stained with GSDMD (1:50, Abcam, USA) or F4/80 (1:100, Immunoway, China) at 4 °C for overnight incubation. To combine with the primary antibody’s binding sites, Alexa Fluor 488 goat anti-rabbit secondary antibody (1:1000, Proteintech, USA) and Alexa Fluor 555 goat anti-mouse secondary antibody (1:1000, Proteintech, USA) were used. Cell nuclei were stained with 4′,6-diamidino-2-phenylindole (DAPI) (ServiceBio, Wuhan, China). Images were obtained with a laser scanning confocal microscope (Leica TCS SP8). For immunofluorescent staining of lung tissues, the primary antibodies used were as follows: F4/80 (1:100, Immunoway, China), EpCAM (1:200, CST, USA), MPO (1:100, Immunoway, China), CD31 (1:100, Immunoway, China).

### Transmission electron microscopy (TEM)

Cells were fixed with 2.5% glutaraldehyde at 4 °C for 4 h, and then rinsed with PBS three times for 15 min each time. After that, cells were fixed with 1% OsO_4_ at room temperature for 2 h, and then rinsed with PBS three times for 15 min each time. Samples were dehydrated through a graded series of ethanol (50, 70, 80, 90, 95, and 100%) for 10 min, alcohol/acetone mixture (1:1) for 10 min, and acetone twice for 10 min. Then, acetone/Spurr resin mixtures (1:1) were used for infiltration for 1 h, and samples were immersed in pure resin at 4 °C overnight. Then, samples were transferred to gelatin capsules for solidification at 37 °C overnight and 60 °C for 48 h, respectively. The samples were sectioned to a thickness of 70 nm and attached to copper grids with Formvar film (300 mesh). At last, the sections were performed staining with 2% uranyl acetate for 30 min and 3% lead citrate for 30 min, respectively. A transmission electron microscope (Thermo Scientific Talos L120C) was used to detect representative images of samples. Mitochondrial numbers were counted manually, and damaged mitochondria were identified by loss of cristae and/or broken mitochondrial outer and/or inner membranes.

### Statistical analysis

Statistical analyses were performed using GraphPad Prism 9 software. Data from independent experiments were expressed as the mean ± SD and unpaired two-tailed Student’s *t* test or one-way ANOVA with a Tukey post-hoc test. For all analysis, *p* < 0.05 was considered to be statistically significant (*p < 0.05, **p < 0.01, ***p < 0.001, and ****p < 0.0001, NS, not significant). Representative data were obtained from at least three independent experiments. The investigators were blinded to allocation during experiments and outcome assessment.

### Reporting summary

Further information on research design is available in the Nature Portfolio Reporting Summary linked to this article.

## Supplementary information


Supplementary Information
Description of Additional Supplementary Files
Supplementary Data 1
Reporting Summary
Transparent Peer Review File


## Source data


Source Data


## Data Availability

The RNA-sequencing and mass spectrometry (lipidomics) data generated in this study have been deposited in the China National Center for Bioinformation (CNCB) database under BioProject accession code PRJCA066752 and PRJCA066880 (https://ngdc.cncb.ac.cn). These data are publicly accessible on the CNCB for all researchers without application restrictions. The data that support the findings of this study are available within the Supplementary Information and the Source Data files. All other data are available in the article and its Supplementary files or from the corresponding author upon request. Source data are provided with this paper.
