# Supplementary material for: A potassium-based single-atom catalyst enables acute lung injury immunotherapy in mice by inhibiting macrophage pyroptosis
Source: Nat Commun. 2026 Aug 4;17:9400. doi: 10.1038/s41467-026-76331-8 (PMC13534642; doi:10.1038/s41467-026-76331-8)
Supplement: Supplementary file 1 [file 41467_2026_76331_MOESM1_ESM.pdf]

Supplementary Information

A Potassium-based Single-Atom Catalyst Enables Acute Lung Injury Immunotherapy in Mice by Inhibiting Macrophage Pyroptosis

1. Reagents and Materials

Dopamine hydrochloride ($C_8H_{11}NO_2$·HCl, 98%) was purchased from Shanghai Aladdin Chemical Reagent Company (Shanghai, China). Tris (hydroxymethyl) methyl aminomethane (Tris, $C_4H_{11}NO_3$, BR) was purchased from Sinopharm. Potassium chloride (KCl, AR), Hydrogen peroxide (H_2O_2, 30%, GR) and ethanol (AR) were purchased from Xilong Scientific. Phosphate Buffered Solution (PBS, pH=7.4) was obtained from Biosharp. Catalase Assay Kit and Total Superoxide Dismutase Kit were purchased from Shanghai Beyotime Biotechnology Co., Ltd. PI (HY-D0815) and CCK-8 (HY-K0301) cell proliferation and cytotoxicity assay kit were purchased from MedChemExpress. JC-1 (40705ES08), mitoSOX (40778ES50) and Reactive Oxygen Species Assay Kit (50101ES01) were purchased from Yeasen Biotechnology (Shanghai) Co., Ltd. Fluo-8-AM was purchased from Shanghai Maokang Biotechnology. Alanine aminotransferase (ALT), aspartate aminotransferase (AST), blood urea nitrogen (BUN) and creatinine (CREA) test kits were acquired from Nanjing Jiancheng Technology Co. Ltd. All chemicals and solvents used were analytical grade.

2. Characterization of K-SAC

Transmission electron microscopy (TEM) images were recorded on a JEOL JEM-F200 microscope. Aberration corrected high-angle annular dark-field scanning TEM (AC HAADF STEM) images, high-resolution TEM (HRTEM), corresponding energy dispersive X-ray spectroscopy (EDX) mapping was obtained on a Spectra Ultra S/TEM (Thermo Fisher Scientific) operated at 300 kV. X-ray diffraction (XRD) patterns were obtained on a Haoyuan DX-2700BH X-ray powder diffractometer (Cu Kα, λ=1.5405 Å, 40 mA, and 40 kV). X-ray photoelectron spectroscopy (XPS) data were carried out at a Thermo Escalab 250XI XPS spectrometer. Raman spectra were collected using a WITec alpha300R. Fourier Transform Infrared Spectrometer (FTIR) spectra were obtained on a Thermo Fisher Scientific Nicolet iN10 spectrometer with background correction by KBr powder. The dynamic light scattering (DLS) measurements were performed on a Malvern Zetasizer Nano ZSE. The K concentration of K-SAC was quantified by inductively coupled plasma-optical emission spectrometry (ICP-OES, Agilent 730).

The X-ray absorption spectroscopy (XAS) at the K K-edge was performed at the Singapore Synchrotron Light Source (SSLS) center, where a Si (111) double crystal was used as the monochromator. The storage ring was working at the energy of 2.5 GeV with an average electron current of below 200 mA. The K K-edge XANES data were collected in a fluorescent mode.

3. DFT calculation

All density functional theory (DFT) calculations were performed using the Vienna Ab Initio Software Package (VASP) within the framework of the generalized gradient approximation (GGA) using the Perdew, Burke, and Ernzerhof (PBE) formulation[1, 2, 3]. The dispersion interactions were described through the Grimme's DFT-D3 methodology. The projected augmented wave (PAW) potentials were used to describe the ionic cores, and the valence electrons were taken into account using a plane wave basis set with a kinetic energy cutoff of 500 eV[4, 5]. The K-SAC model was constructed according to EXAFS results. The Brillouin zone integration was performed using 3×3×1 Monkhorst-Pack k-point sampling for all kinds of structures. Partial occupancies of the Kohn-Sham orbitals were handled by the Gaussian smearing method with a width of 0.05 eV. The electronic energy was considered self-consistent when the energy change was smaller than 10^{-5} eV. The geometry optimization was considered convergent when the force change was smaller than 0.05 eV/Å. The free energy was calculated according to the equation[6]:

$$G = E_{ads} + ZPE - TS \qquad (1)$$

where G, E_{ads}, ZPE and TS are the free energy, total energy from DFT calculations, zero-point energy and entropic contributions, respectively, with T set to 298 K.

Chemical bonding characteristics were investigated through crystal orbital Hamilton population (COHP) analysis using the Local Orbital Basis Suite Towards Electronic-Structure Reconstruction (LOBSTER) software package.

4. Electrochemical response

A standard three-electrode cell was used for recording cyclic voltammogram (CV) curves. Specifically, a nickel foam electrode (1 × 1 cm) was used as the working electrode, a leak-free saturated calomel electrode and a carbon rod electrode were used

as the reference electrode and the auxiliary electrode, respectively. A mild acidic NaCl aqueous solution (0.1 M, pH= 6.6) containing K-SAC ([K]=10 μM), which was purged with nitrogen for 10 min before the test, was used as the supporting electrolyte solution. The CV measurements were conducted within a voltage range between -1.1 V and 0.1 V at a scan rate of 50 mV/s.

5. Cytotoxicity assay

CCK-8 analysis was used to evaluate the cytotoxicity of nanoparticles. BMDM and PM were cultured in a 96-well plate at 8000 cells/well density, cultured at 37 °C and 5% CO_2 overnight. After that, these cells were treated with K-SAC at different concentrations for another 24 h. Then, the medium was replaced with medium containing 10% Cell Counting Kit 8 (CCK-8) (MedChemExpress, Monmouth Junction, NJ, USA). The plates were incubated at 37 °C and 5% CO_2 for 2 h in the shade. Cell viability was determined with a microplate reader by measuring the optical density at 450 nm.

6. RNA Sequencing and Bioinformatics Analysis

BMDMs treated with LPS+ATP or LPS+ATP+K-SAC were collected and stored at -80 °C for further RNA sequencing by Nanjing Genepioneer Biotechnologies. RNA concentration was measured using Qubit® RNA Assay Kit in Qubit® 2.0 Flurometer (Life Technologies, CA, USA). A total amount of 3 μg RNA per sample was used as input material for the RNA sample preparations. Sequencing libraries were generated using NEB Next® Ultra™ RNA Library Prep Kit for Illumina® (NEB, USA) following the manufacturer's recommendations, and index codes were added to attribute sequences to each sample. The clustering of the index-coded samples was performed on a cBot Cluster Generation System using TruSeq PE Cluster Kit v3-cBot-HS (Illumina) according to the manufacturer's instructions. After cluster generation, the library preparations were sequenced on an Illumina Nova Seq6000 platform, and 150bp paired-end reads were generated.

For data analysis, Raw data (raw reads) of fastq format were first processed through in-house Perl scripts. Then Index of the reference genome was built, and paired-end clean reads were aligned to the reference genome using HISAT2. StringTie was used to

construct and identify both known and novel transcripts from HISAT2 alignment results. StringTie was used to count the reads mapped to each gene, and readcount for each gene was obtained from the mapping results. And then FPKM of each gene was calculated based on the length of the gene and reads count mapped to this gene.

Differential expression analysis of two conditions/groups was performed using the DESeq R package (1.10.1). The resulting *p* values were adjusted using the Benjamini and Hochberg's approach for controlling the false discovery rate. Genes with an adjusted P-value <0.05 found by DESeq were assigned as differentially expressed. Pathway analysis was performed using significantly upregulated and downregulated genes between the LPS+ATP dataset and the LPS+ATP+K-SAC dataset. Gene functional was annotated based on the following databases: Nr (NCBI non-redundant protein sequences); Nt (NCBI non-redundant nucleotide sequences); Pfam (Protein family); KOG/COG (Clusters of Orthologous Groups of proteins); Swiss-Prot (A manually annotated and reviewed protein sequence database); KO (KEGG Ortholog database) and GO (Gene Ontology). We used the GOseq R packages based on Wallenius non-central hyper-geometric distribution[7] for GO analysis and KOBAS[8] software to test the statistical enrichment of differential expression genes in KEGG pathways.

7. Lipid UPLC-QE MS Analysis

For lipidomics profiling, BMDMs treated with either LPS+ATP or LPS+ATP+K-SAC were collected and processed for analysis using ultrahigh-performance liquid chromatography coupled with Q-Exactive MS (UPLC-QE MS). Lipids were extracted from the BMDMs using a solution of methyl tert-butyl ether (MTBE)/methanol/water (v/v/v, 10:2:5) containing 2 μg/mL of the internal standard (LPC 17:0). The samples were first vortex-mixed for 60 seconds to ensure thorough homogenization, followed by sonication for 15 minutes to facilitate lipid extraction. After allowing the mixture to stand at room temperature for 5 minutes, it was centrifuged at 13,000 × g for 15 minutes at 4 °C. Subsequently, 400 μL of the MTBE layer was carefully transferred to an Eppendorf tube and the solvent was evaporated under a stream of nitrogen. The dried lipid extracts were then reconstituted in 100 μL of a dichloromethane/methanol (v/v, 1:1) solution and transferred to auto-sampler vials for analysis. UPLC-QE MS analysis

was carried out on a Vanquish HPLC system (Thermo Scientific, Germany) integrated with a Q-Exactive Plus Orbitrap mass spectrometer (Thermo Scientific). Chromatographic separation of the lipid samples was achieved using a Waters XBridgeTM BEH C18 column (2.1 mm×100 mm, 2.5 µm; Waters, Milford, MA). Data were imported into SIMCA-P 11.0 (Umetrics) software for multivariate statistical analysis after normalization. Orthogonal partial least squares discriminant analysis (OPLS-DA) scatter plots were obtained to show the potential biomarkers of lipomics from the obtained data.

8. Assessment of mitochondrial function

Mitochondrial function was evaluated using a JC-1 Assay Kit (Yeason Biotech, Shanghai, China), and JC-1 monomer/polymer ratios were analyzed. We measured mitochondrial reactive oxygen species (mtROS) using mitoSOX (Yeason Biotech, Shanghai, China) following the manufacturer's instructions. The fluorescence intensities of mitoSOX and JC-1 were obtained with a laser scanning confocal microscope (Leica TCS SP8).

9. H&E and lung injury score analysis

Left lung tissues were fixed in 4% paraformaldehyde for 72 h, embedded in paraffin, cut into 5-µm-thick sections, and H&E stained to evaluate the severity of lung injury and the inflammatory response. Each tissue section was observed under a light microscope. The degree of lung injury was estimated based on semiquantitative scoring standard established in our previous studies[9]. The lung injury score was assessed by two blinded pathologists. The histologic features considered for scoring included interstitial/intra-alveolar edema, hemorrhage, inflammation, and alveolar septal thickening/hyaline membrane formation. On a 5-point scale, each item was given a grade of 0 (little), 1 (mild), 2 (moderate), 3 (severe), or 4 (maximum), correspondingly. The total of all the variables was used to calculate the lung injury score (total score, 0-16).

10. Reactive Oxidative Species staining of lung sections and cells

Intracellular ROS was detected with the ROS assay kit (Yeasen Biotechnology, Shanghai, China) according to the manufacturer's protocol. The fluorescence

intensities of ROS were obtained with a laser scanning confocal microscope (Leica TCS SP8).

11. Cytosolic Ca^{2+} detection

Fluo-8-AM (#MX4505, Shanghai Maokang Biotechnology, 50 μg) was dissolved in 30 μL DMSO for storage. The staining solution is as follows: 1 μL Fluo-8-AM, 1 μL Pluronic F-127 (Shanghai Maokang Biotechnology, Shanghai, China), and 300 μL HBSS. After treatment with K-SAC, LPS and ATP treatment, Fluo-8-AM was added into cells and Incucyte was used to detect the changes of fluorescence intensity of each group for 5 hours. Fluorescence was normalized to the fluorescence intensity of 0 hours.

12. Blood biochemistry

Blood samples were harvested from mice with or without K-SAC treatment for 3 days. ALT, AST, BUN, and CREA analyses were conducted following the manufacturer's instructions (Nanjing Jiancheng Bioengineering Institute, Nanjing, China).

13. RNA interference

Small interfering RNAs (siRNAs) targeting GSDMD were obtained from GenePharma. Sequence was shown in Supplementary Table 4. Transfection was performed using the INTERFERin reagent (101000028; Polyplus) according to the manufacturer's protocol. Knockdown efficiency was confirmed by RT-qPCR (data not shown).

Supplementary Figures and Figure legends

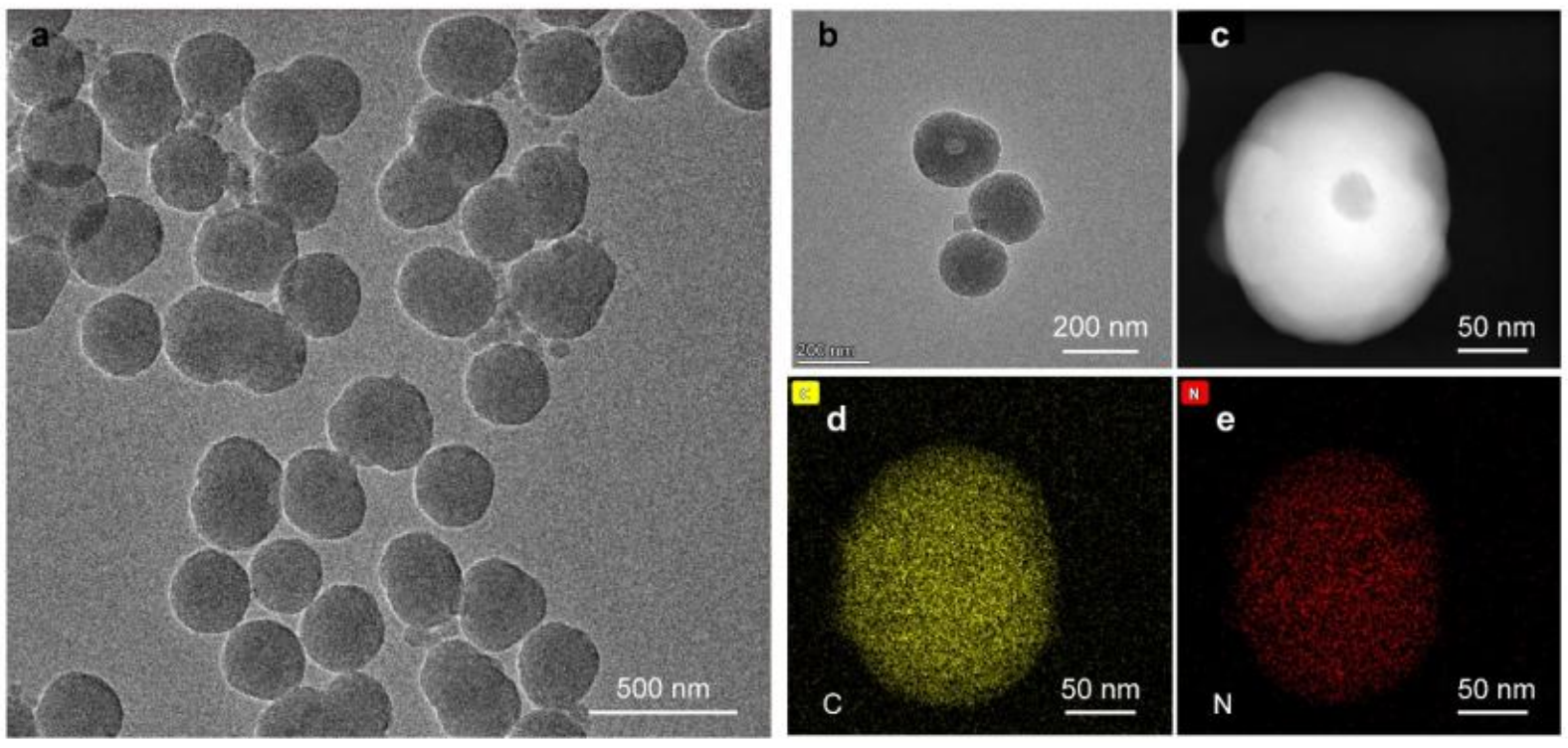

Supplementary Fig. 1. Characterization of PDA and NC.

a, TEM image of PDA nanospheres. n = 3 samples with similar results. **b**, TEM image and **c-e**, corresponding element mappings of NC. n = 3 samples with similar results.

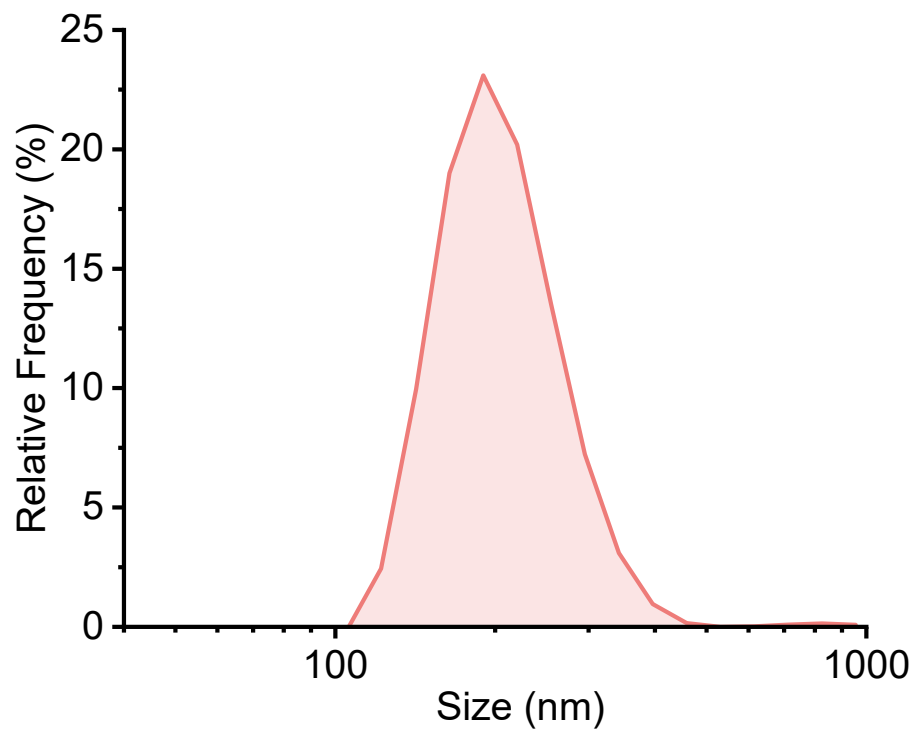

Supplementary Fig. 2. Size distribution of K-SAC in DIW by DLS.

Source data are provided as a Source Data file.

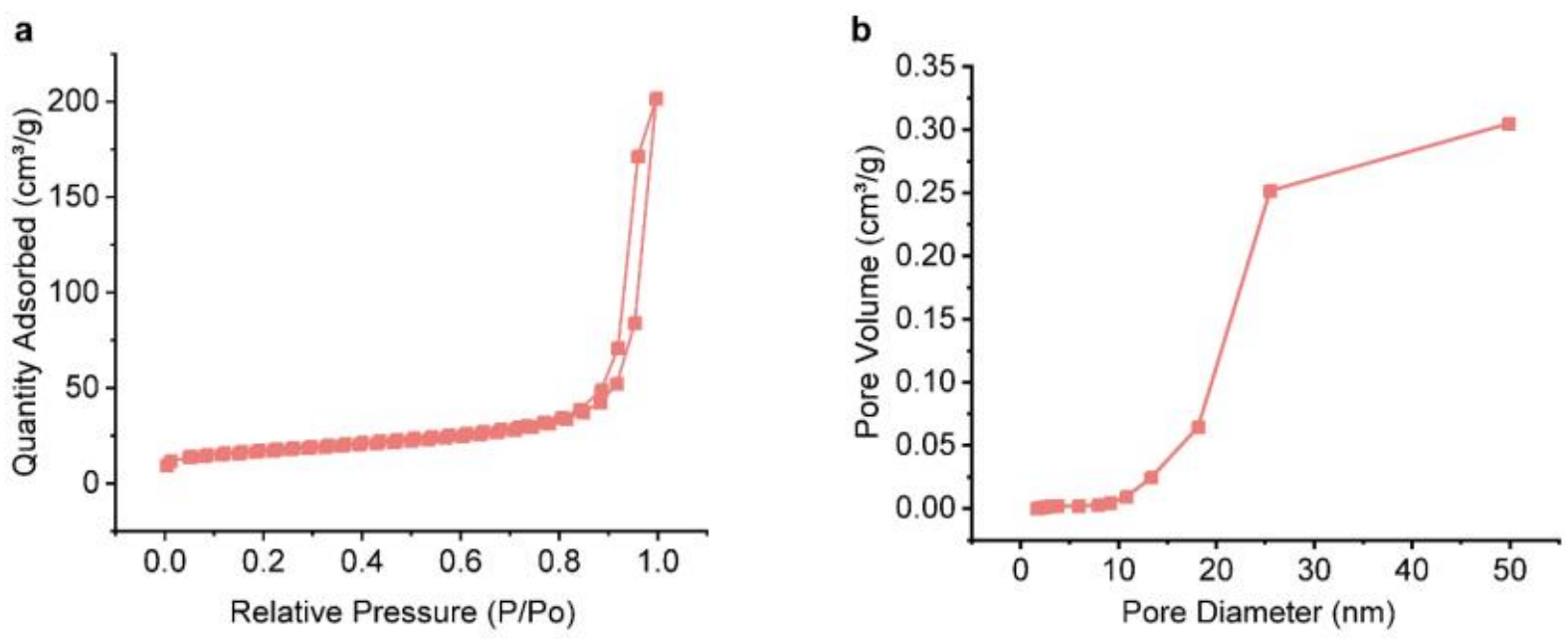

Supplementary Fig. 3. N_2 sorption isotherm experiments of K-SAC.

a, N_2 sorption isotherms of K-SAC**. b,** The corresponding pore size distribution curves of K-SAC. Source data are provided as a Source Data file.

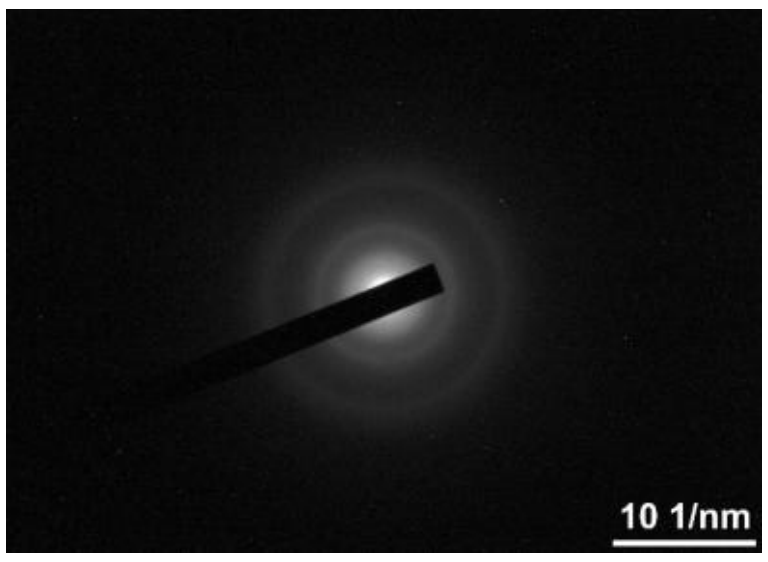

Supplementary Fig. 4. SAED pattern of K-SAC.

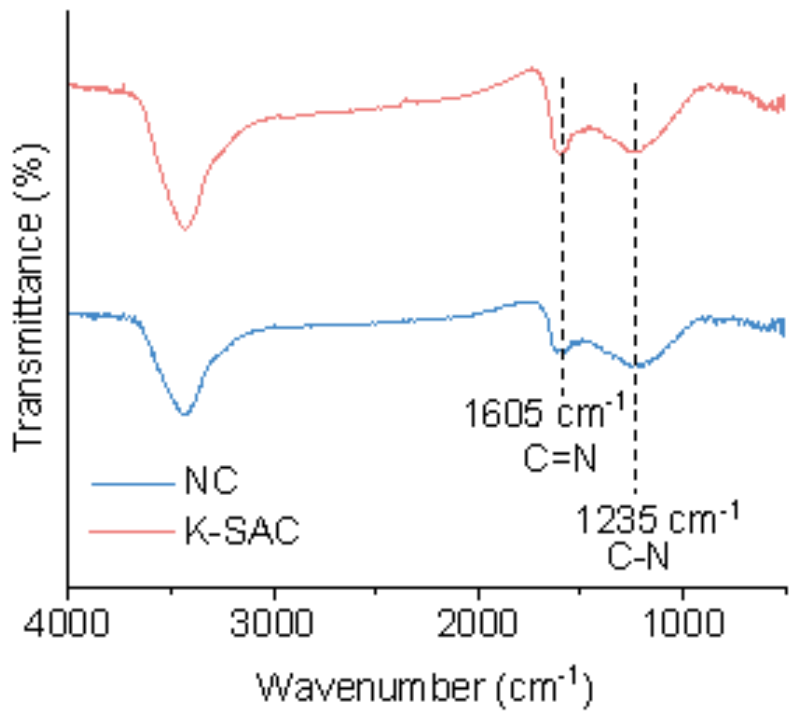

Supplementary Fig. 5. FTIR Spectra of K-SAC and NC.

Source data are provided as a Source Data file.

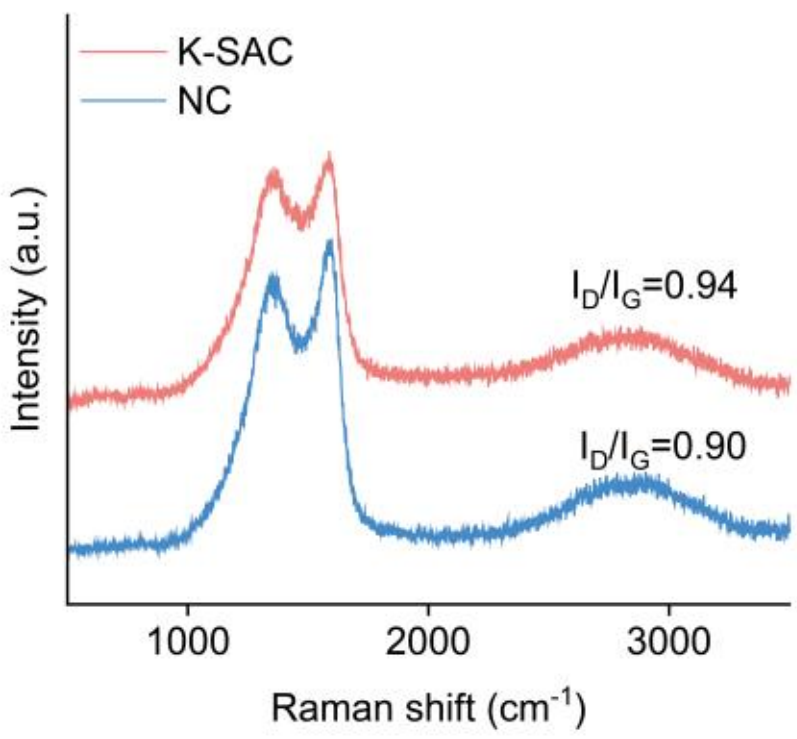

Supplementary Fig. 6. Raman spectra of K-SAC and NC.

Source data are provided as a Source Data file.

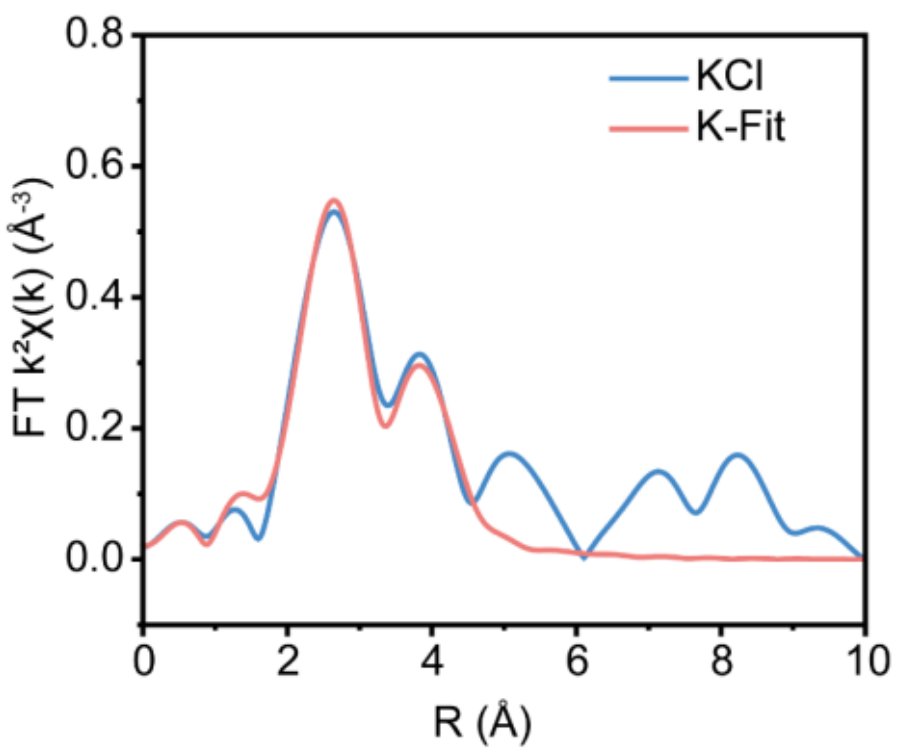

Supplementary Fig. 7. EXAFS fitting for KCl in R space.

Source data are provided as a Source Data file.

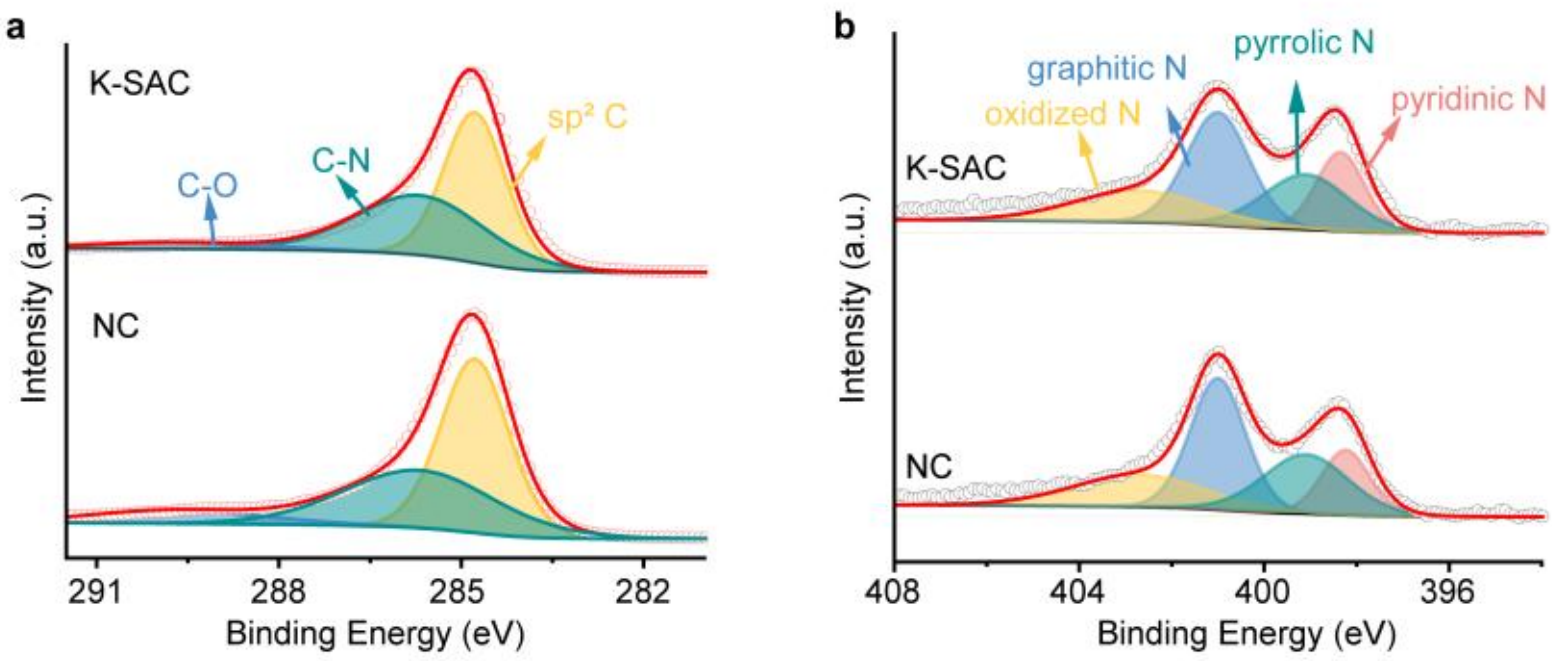

Supplementary Fig. 8. High-resolution XPS results.

a, High-resolution C 1s spectra of K-SAC and NC. **b,** High-resolution N 1s spectra of K-SAC and NC. Source data are provided as a Source Data file.

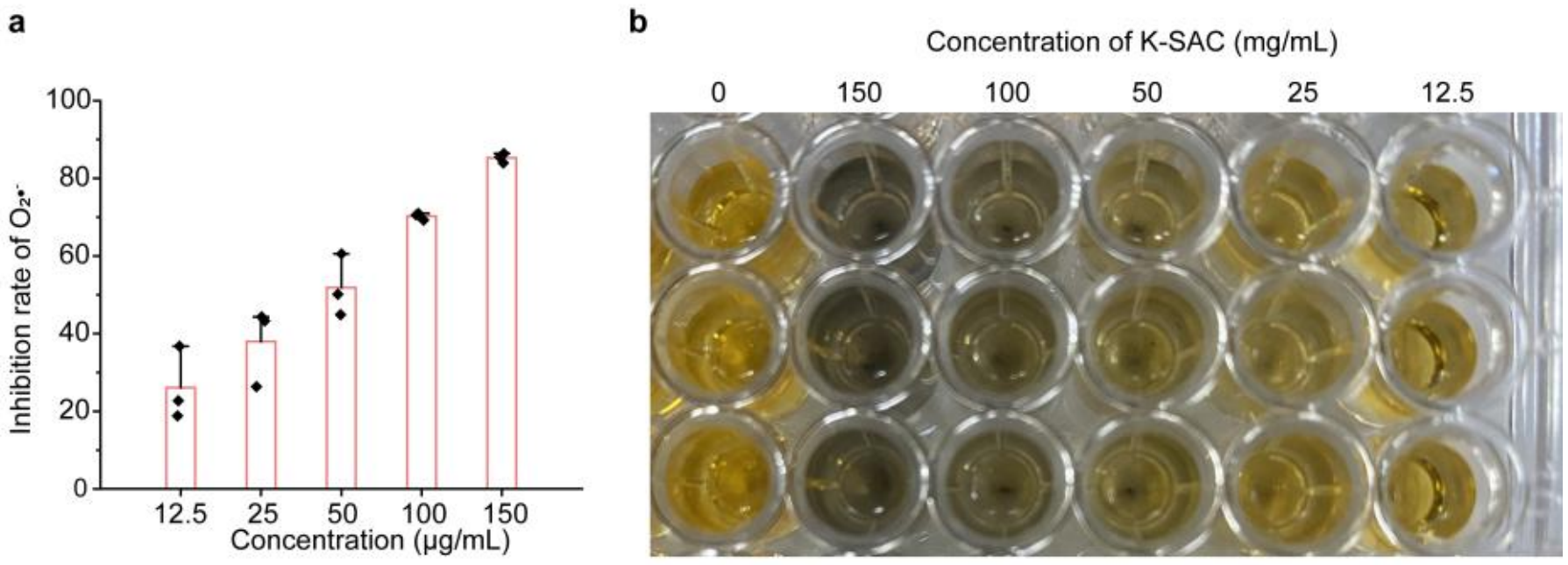

Supplementary Fig. 9. SOD-like activity tests of K-SAC.

a, SOD-like performance and **b,** photographic image of K-SAC at various concentrations (*n* = 3 tests). Data are presented as mean±SD. Source data are provided as a Source Data file.

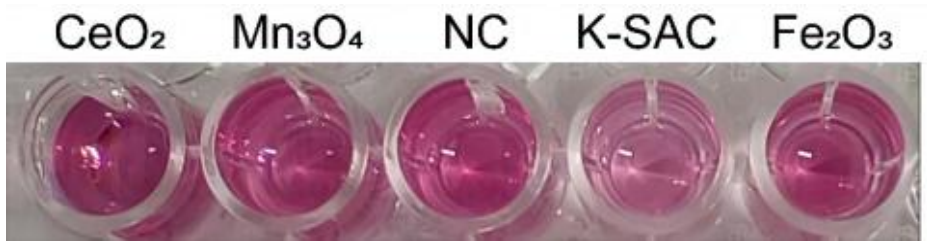

Supplementary Fig. 10. Photographic image of CAT-like performance of different catalysts.

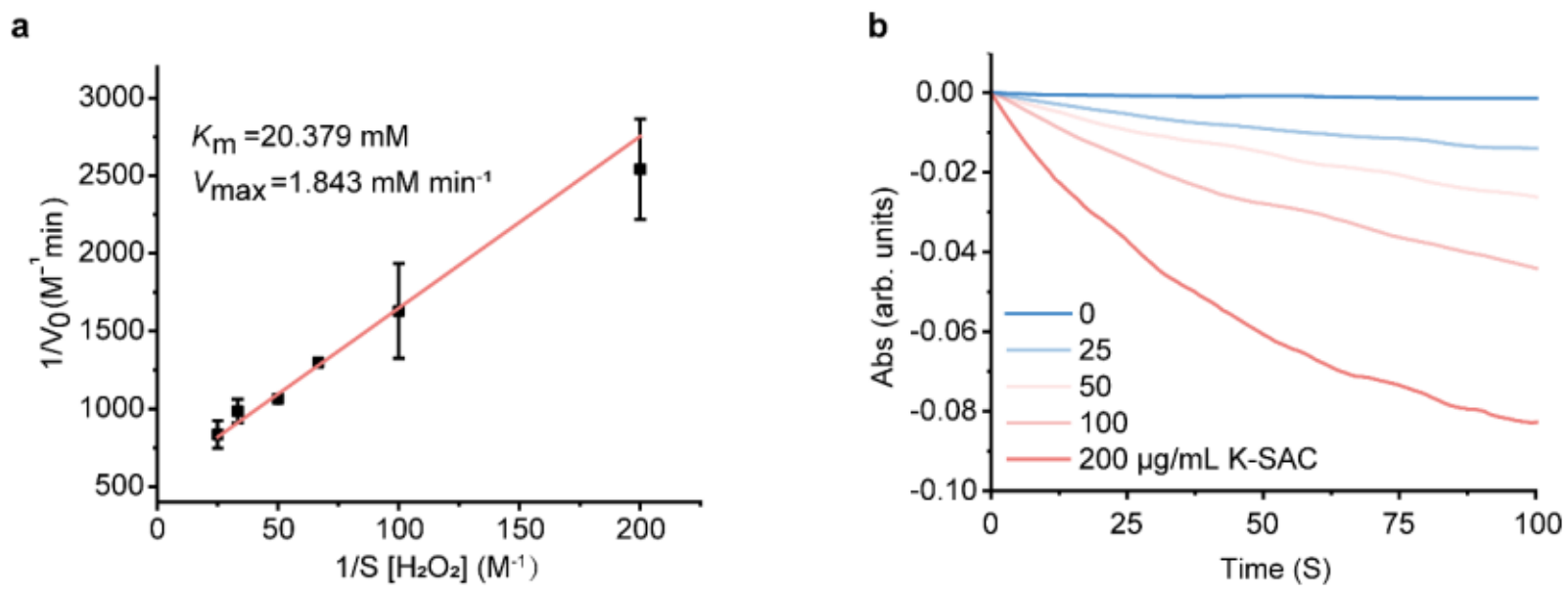

Supplementary Fig. 11. CAT-like activity tests of K-SAC.

a, Lineweaver-Burk plot for K-SAC at diverse concentrations of H_2O_2 (0–40 mM). *n* = 3 tests. Data are presented as mean±SD. **b,** CAT-like assay for the elimination of 10 mM H_2O_2 utilizing diverse concentrations of K-SAC. Source data are provided as a Source Data file.

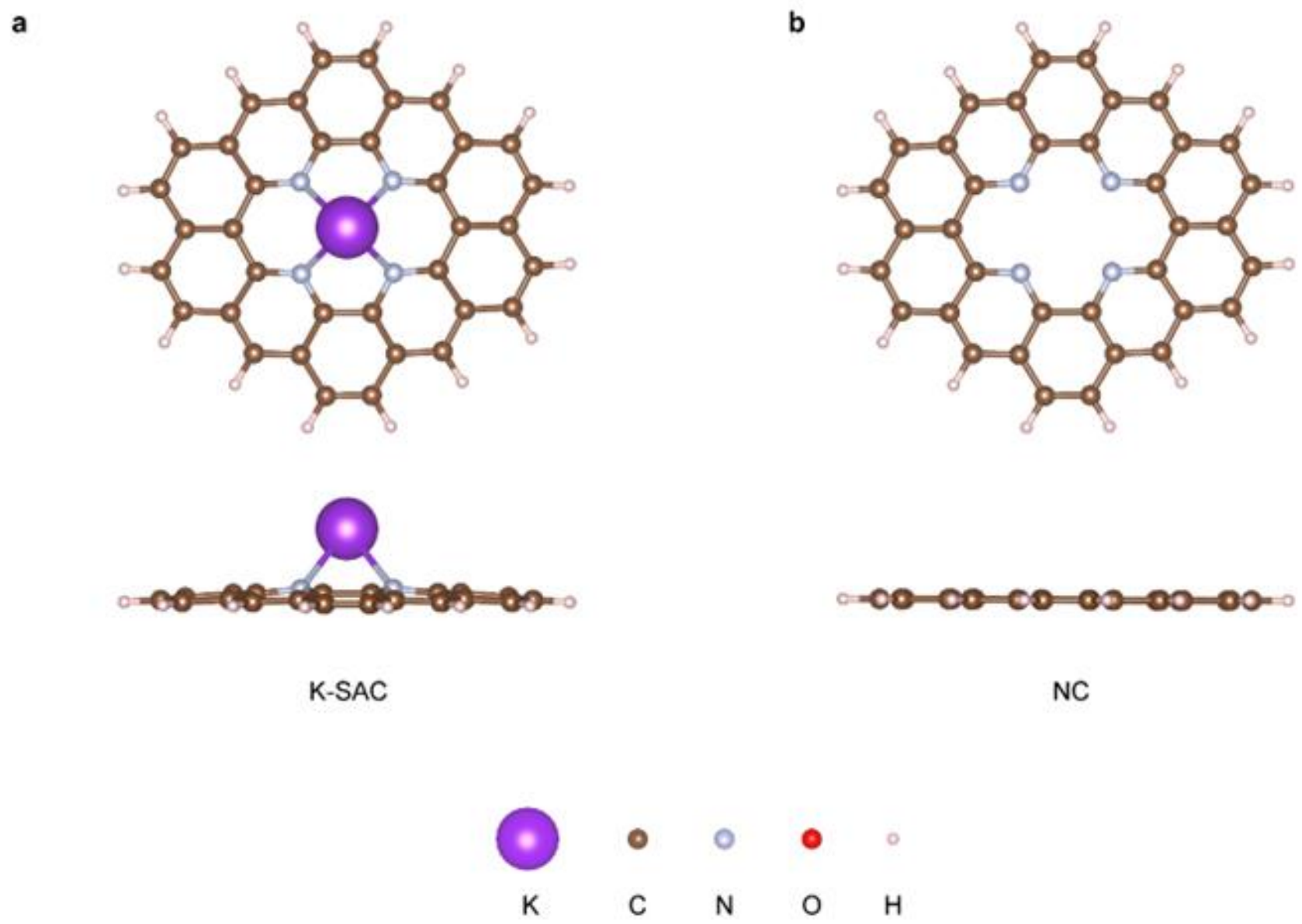

Supplementary Fig. 12. Optimal structural model of K-SAC and NC.

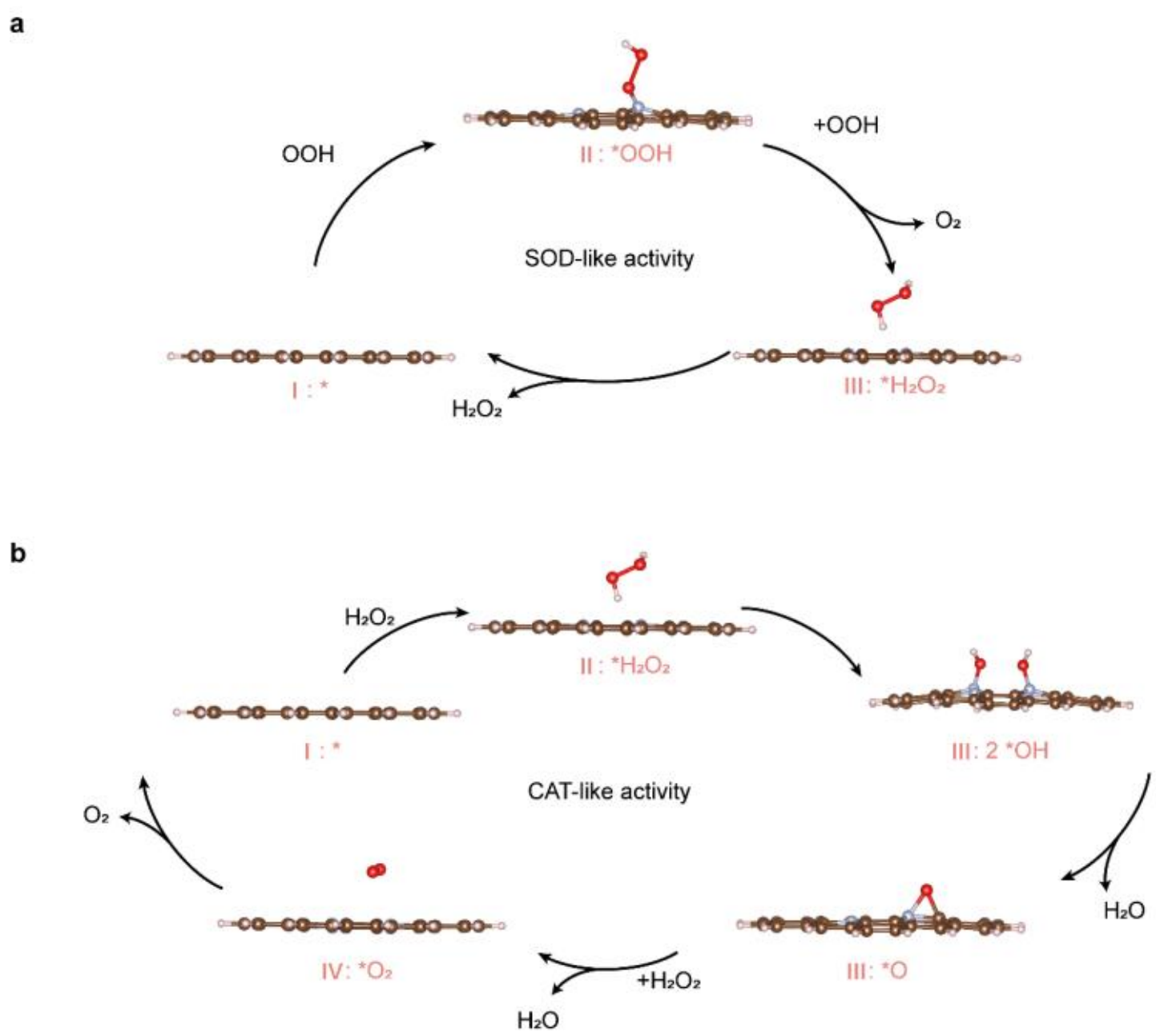

Supplementary Fig. 13. Reaction pathways of NC.

a, DFT calculations for SOD-like reaction pathways of NC. **b,** DFT calculations for CAT-like reaction pathways of NC.

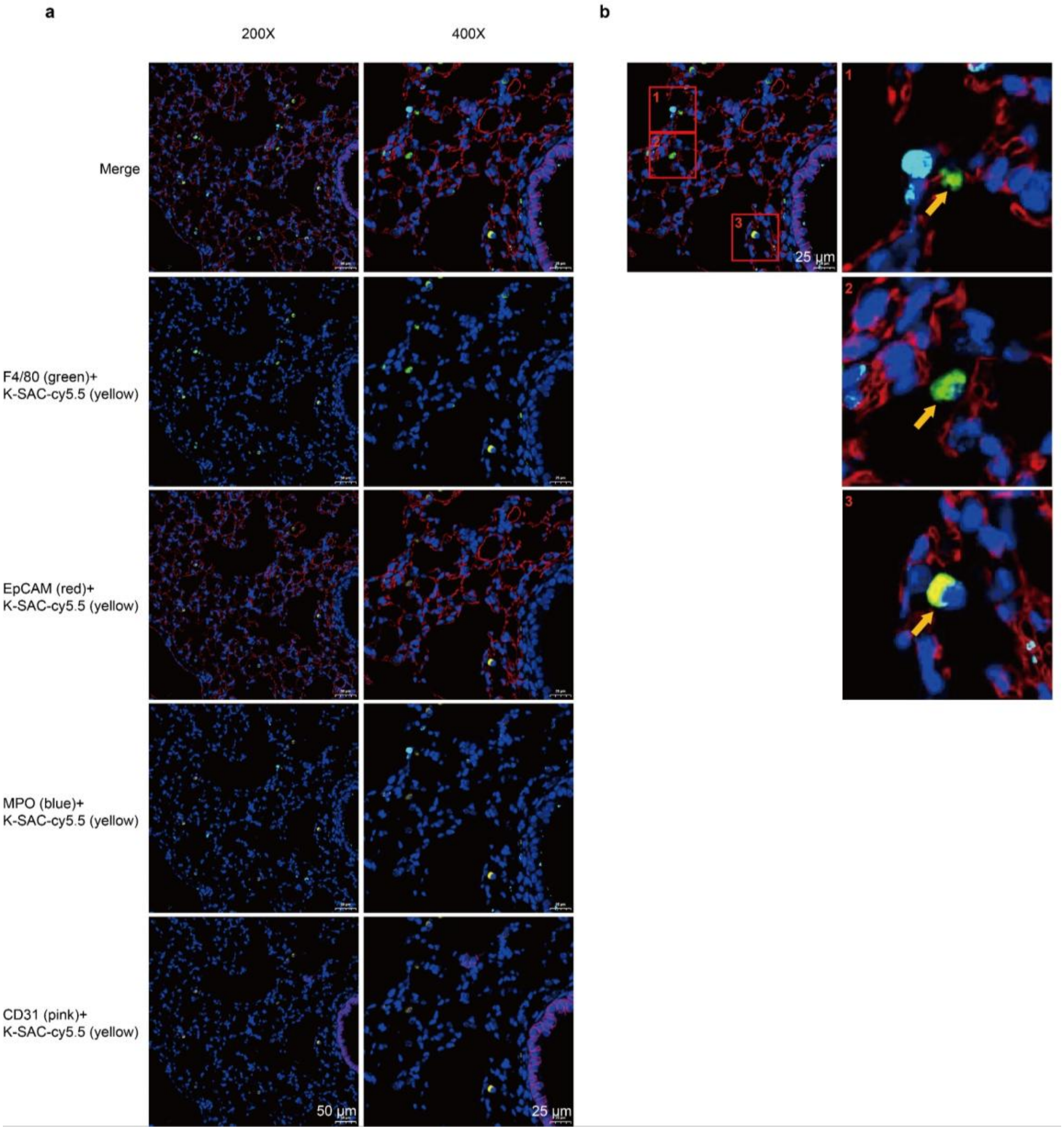

Supplementary Fig. 14. Immunofluorescence co-staining images for different cell types with Cy5.5-labeled K-SAC in mouse lung tissue.

a, Immunofluorescence co-staining images for F4/80 (green), EpCAM (red), MPO (light blue) and CD31 (pink) and K-SAC-cy5.5 (yellow) in mouse lung tissue. **b,** The enlarged immunofluorescen ce.

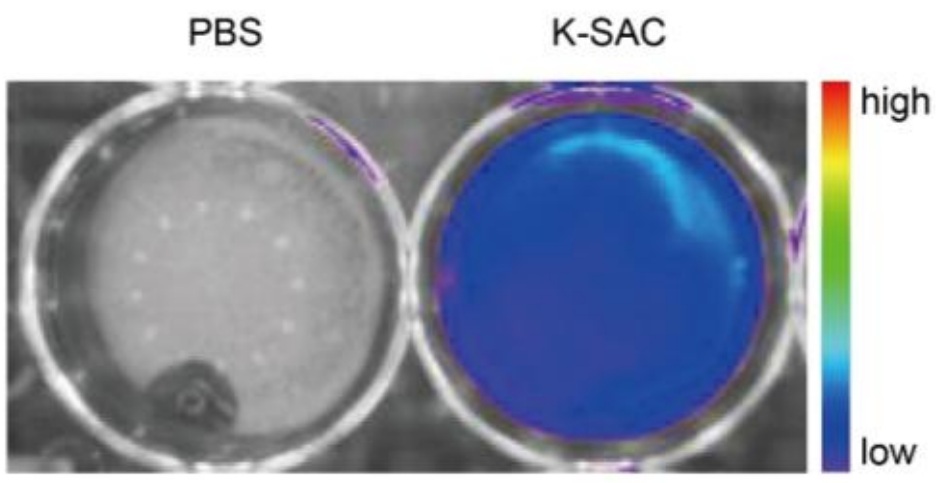

Supplementary Fig. 15. Fluorescence assay of Cy5.5-labeled K-SAC.

PMs seeded on transwell-polycarbonate membranes were incubated with Cy5.5-labeled K-SAC. Relative quantification of particles penetrating to the lower well plate compartment after 24 h.

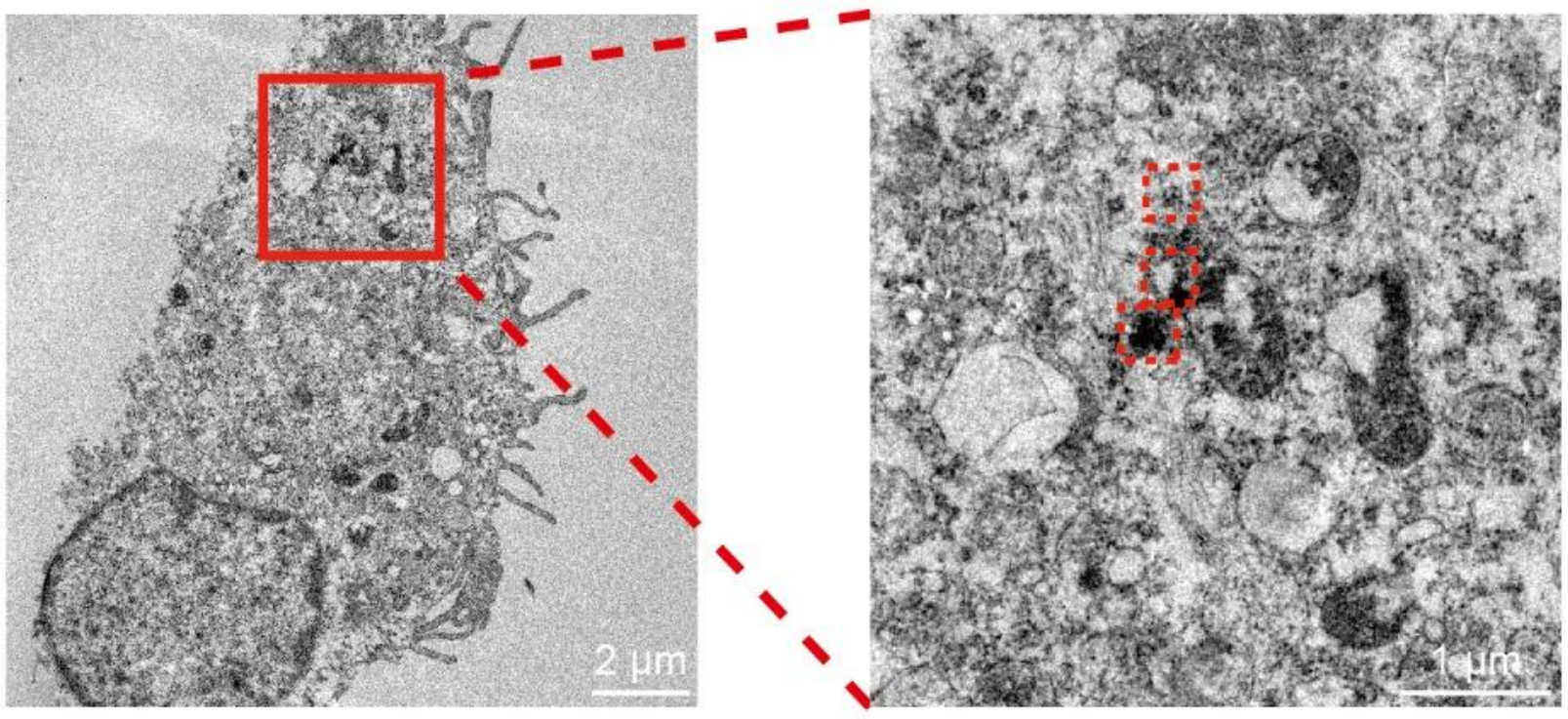

Supplementary Fig. 16. TEM images of K-SAC in PMs.

PMs were treated with K-SAC for 24 h. Representative TEM images revealed that K-SAC was phagocytosed by PMs (Red dotted boxes show the K-SAC) (Left scale bar, 2 μm. Right scale bar, 1 μm).

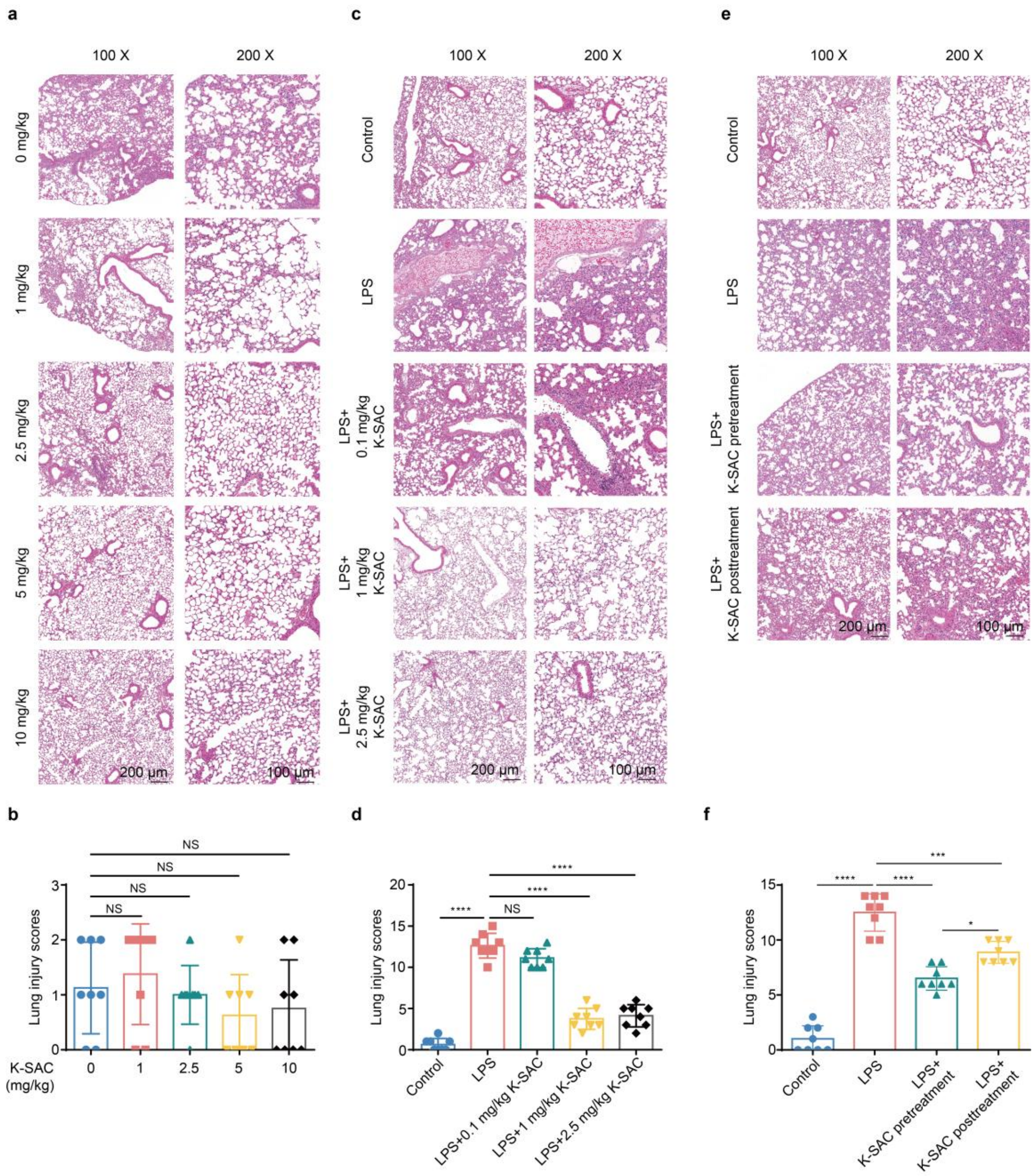

Supplementary Fig. 17. Pulmonary safety evaluation and efficacy detection of different doses of K-SAC.

a,b, Representative H&E staining of mouse lung tissues following nasal inhalation of different doses of K-SAC (1 mg/kg, 2.5 mg/kg, 5 mg/kg, 10 mg/kg) **(a)** and lung injury score **(b)** (scale bar: left, 200 μm; right, 100 μm). **c,d,** Representative H&E staining of mouse lung tissues following LPS injection and nasal inhalation of different doses of K-SAC (0.1 mg/kg, 1 mg/kg, 2.5 mg/kg) **(c)** and lung injury score **(d)** (scale bar: left, 200 μm; right, 100 μm). **e,f,** Representative H&E staining of mouse lung tissues following nasal inhalation of K-SAC (1 mg/kg) before or after LPS injection **(e)** and lung injury score **(f)** (scale bar: left, 200 μm; right, 100 μm). The results are presented as

mean ± SD with $n = 8$ **(b,d,f)** independent samples for each group. Statistical significance was calculated via one-way ANOVA, Tukey's multiple comparisons **(b,d,f)**. Source data are provided as a Source Data file.

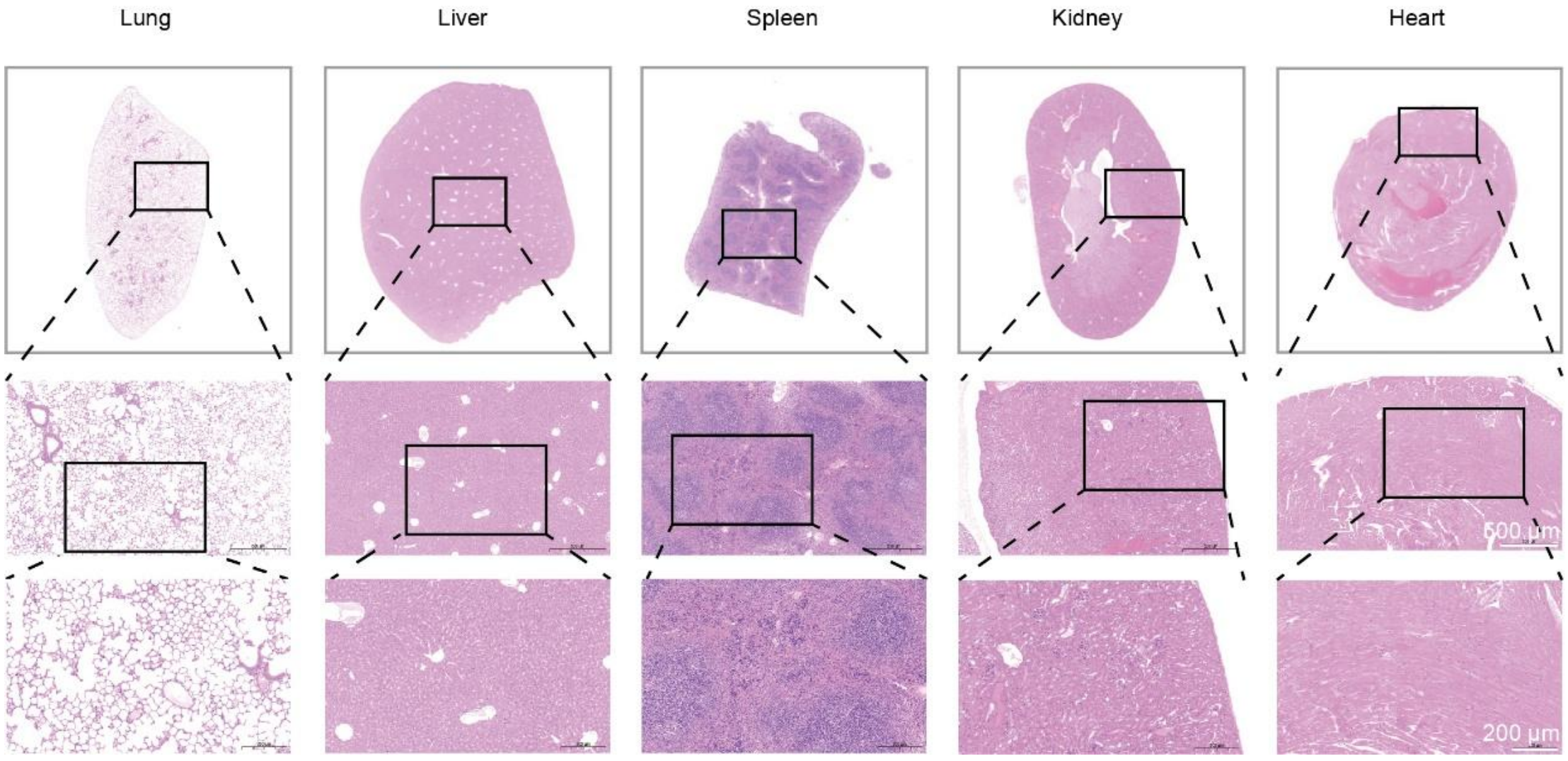

Supplementary Fig. 18. In vivo toxicity assessment of K-SAC.

Representative H&E staining of mouse lung, liver, spleen, kidney, and heart following nasal inhalation of 1 mg/kg K-SAC (scale bar: middle, 500 μm; lower, 200 μm).

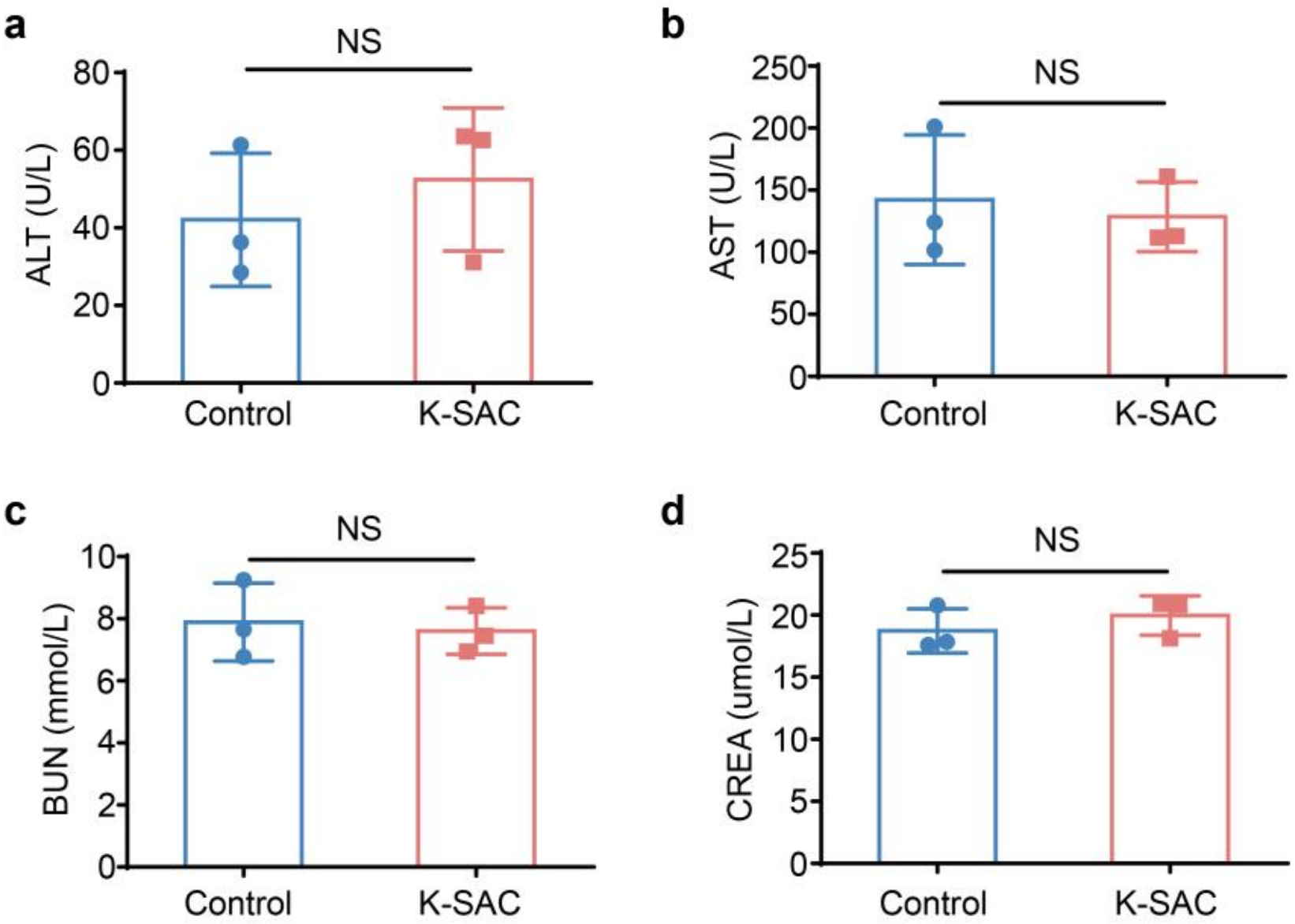

Supplementary Fig. 19. Liver and kidney toxicity assessment of K-SAC.

The expressions of ALT, AST, CREA and BUN in mice serum after K-SAC treatment. The results are presented as mean ± SD with $n = 3$ independent samples for each group. Statistical significance was calculated via unpaired t-test, two-tailed. Source data are provided as a Source Data file.

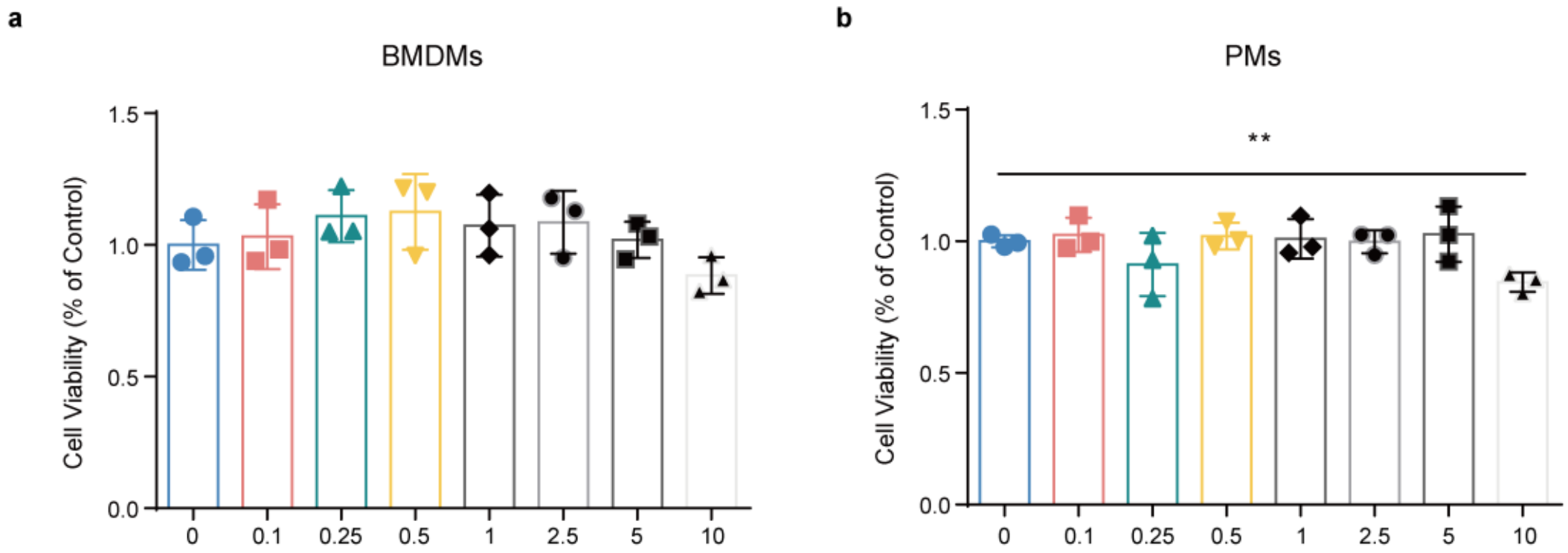

Supplementary Fig. 20. In vitro cytotoxicity assessment of K-SAC.

CCK-8 assay of BMDMs and PMs treated with PBS (control group) or various doses of K-SAC. The results are presented as mean ± SD with $n = 3$ independent samples for each group. Source data are provided as a Source Data file.

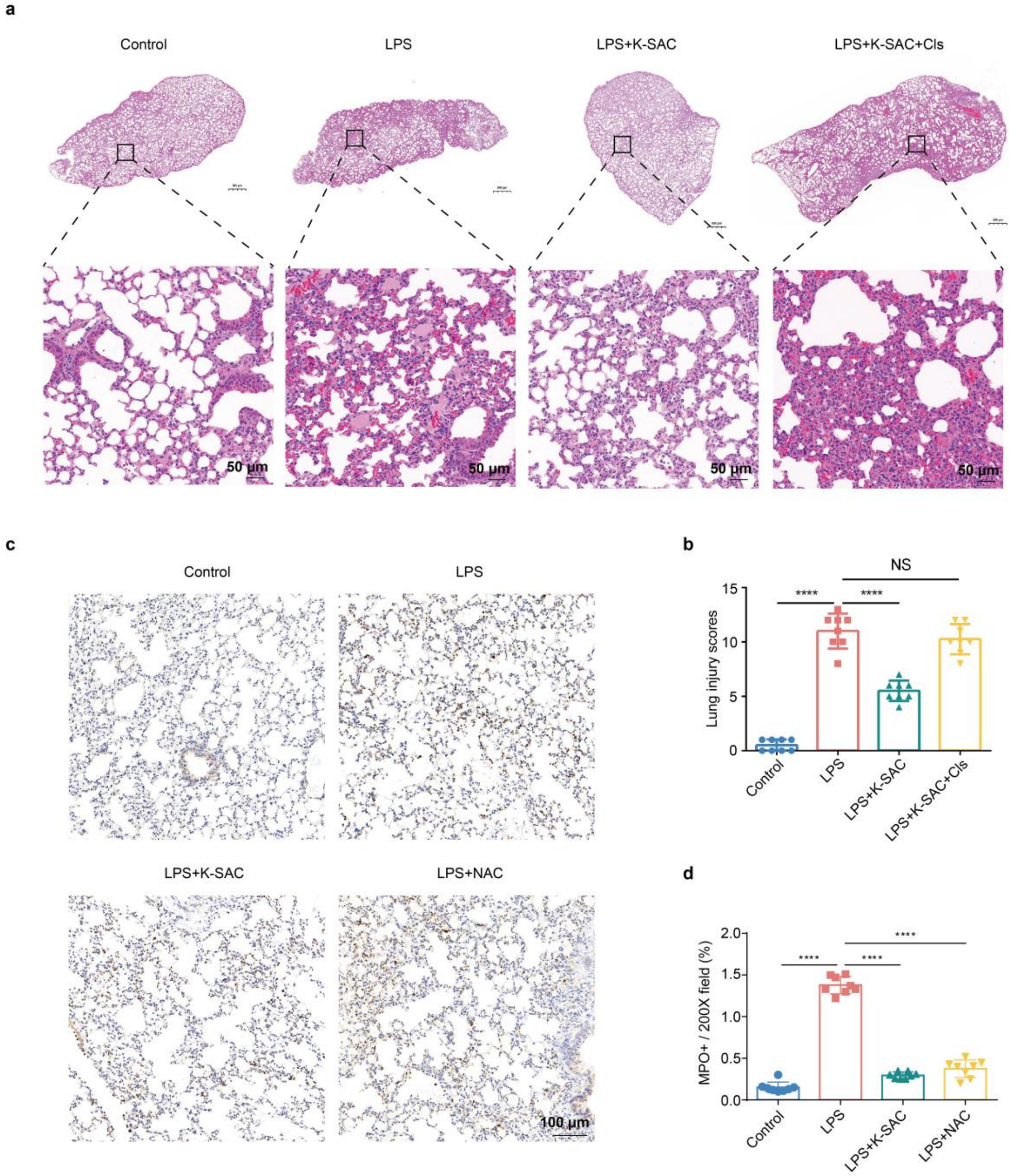

Supplementary Fig. 21. Macrophages play a critical role in the alleviation of lung injury by K-SAC.

a,b, The therapeutic effect of K-SAC was significantly attenuated after intraperitoneal macrophage depletion. **c,d,** Immunohistochemistry detection of neutrophil myeloperoxidase (MPO) in lung tissue. The results are presented as mean ± SD with $n = 8$ **(b,d)** independent samples for each group. Statistical significance was calculated via one-way ANOVA, Tukey's multiple comparisons **(b,d)**. Source data are provided as a Source Data file.

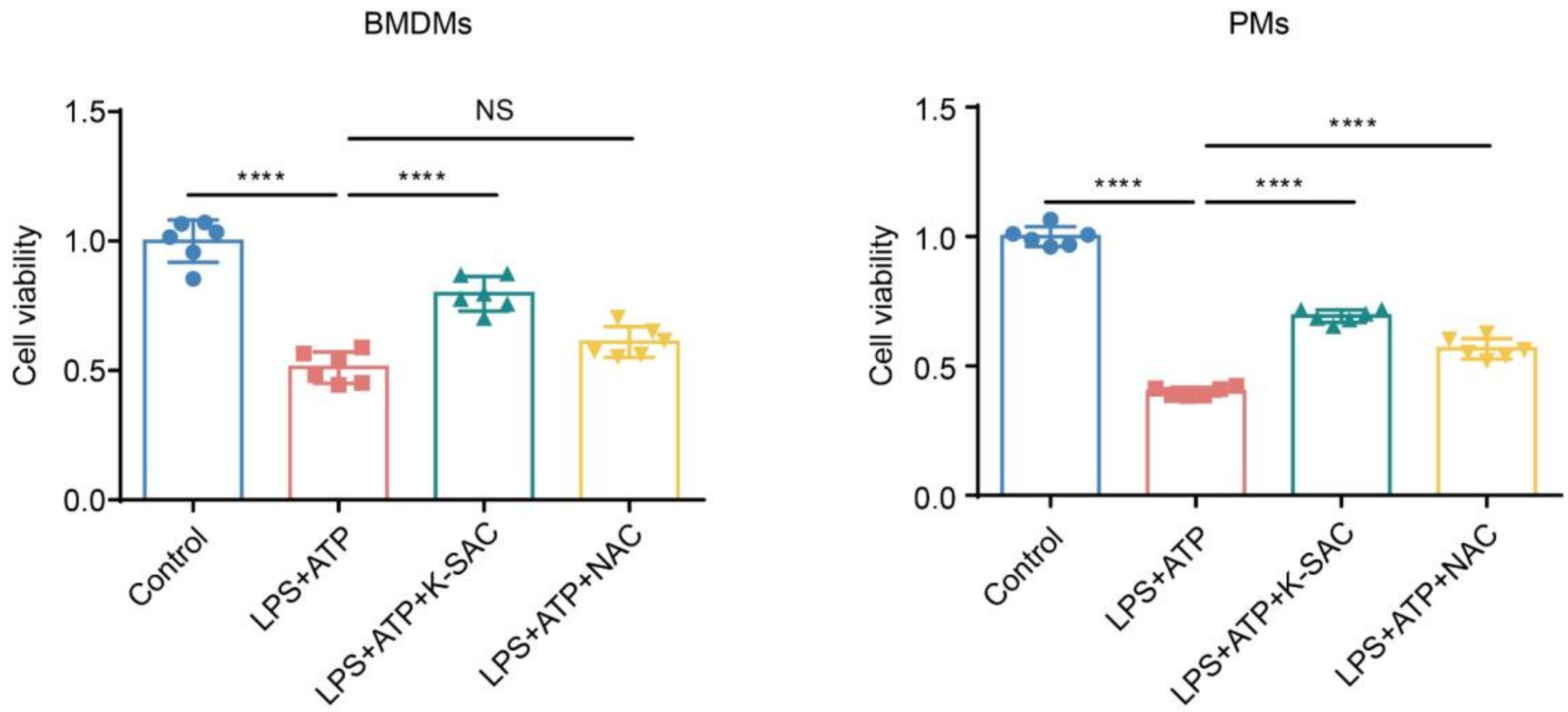

Supplementary Fig. 22. CCK8 analysis of cytoprotective effect of K-SAC.

CCK-8-assessed cell viability of LPS (1 μg/mL) and ATP (5mM)-treated BMDMs and PMs after 24 h treatment with K-SAC (0.1 μg/mL) or NAC (0.25 mM). The results are presented as mean ±SD with $n = 6$ independent samples for each group. Statistical significance was calculated via one-way ANOVA, Tukey's multiple comparisons. Source data are provided as a Source Data file.

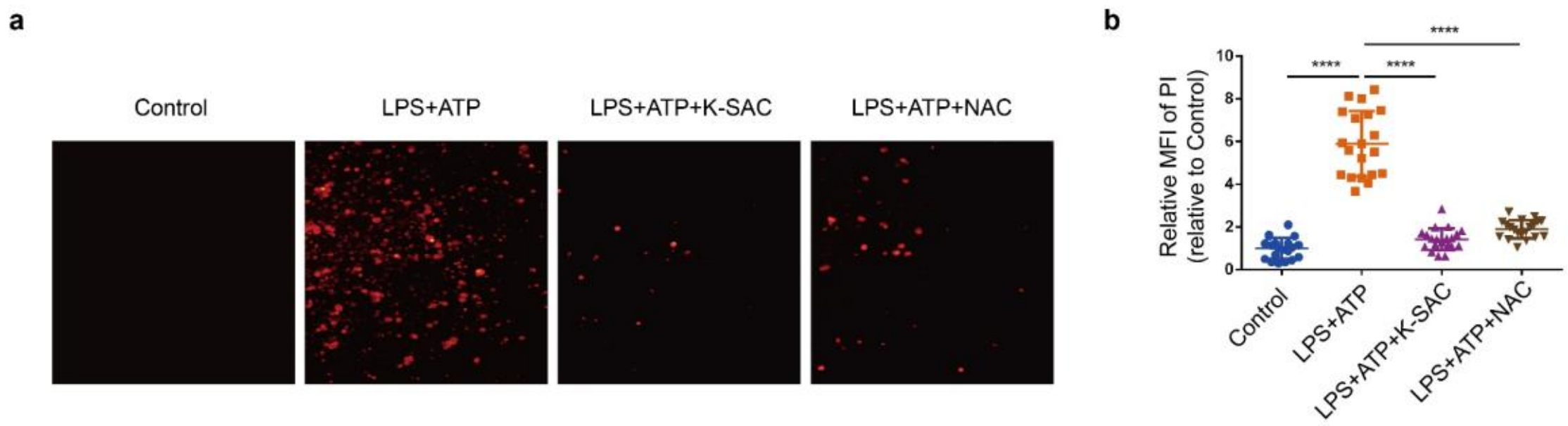

Supplementary Fig. 23. PI analysis of cytoprotective effect of K-SAC.

a,b, Representative PI immunofluorescent staining images **(a)** and MFI quantification **(b)** in LPS and ATP-stimulated PMs with K-SAC or NAC treatment (Scale bar: 200 μm). Data are presented as mean ± SD. Statistical significance was calculated via one-way ANOVA, Tukey's multiple comparisons. Source data are provided as a Source Data file.

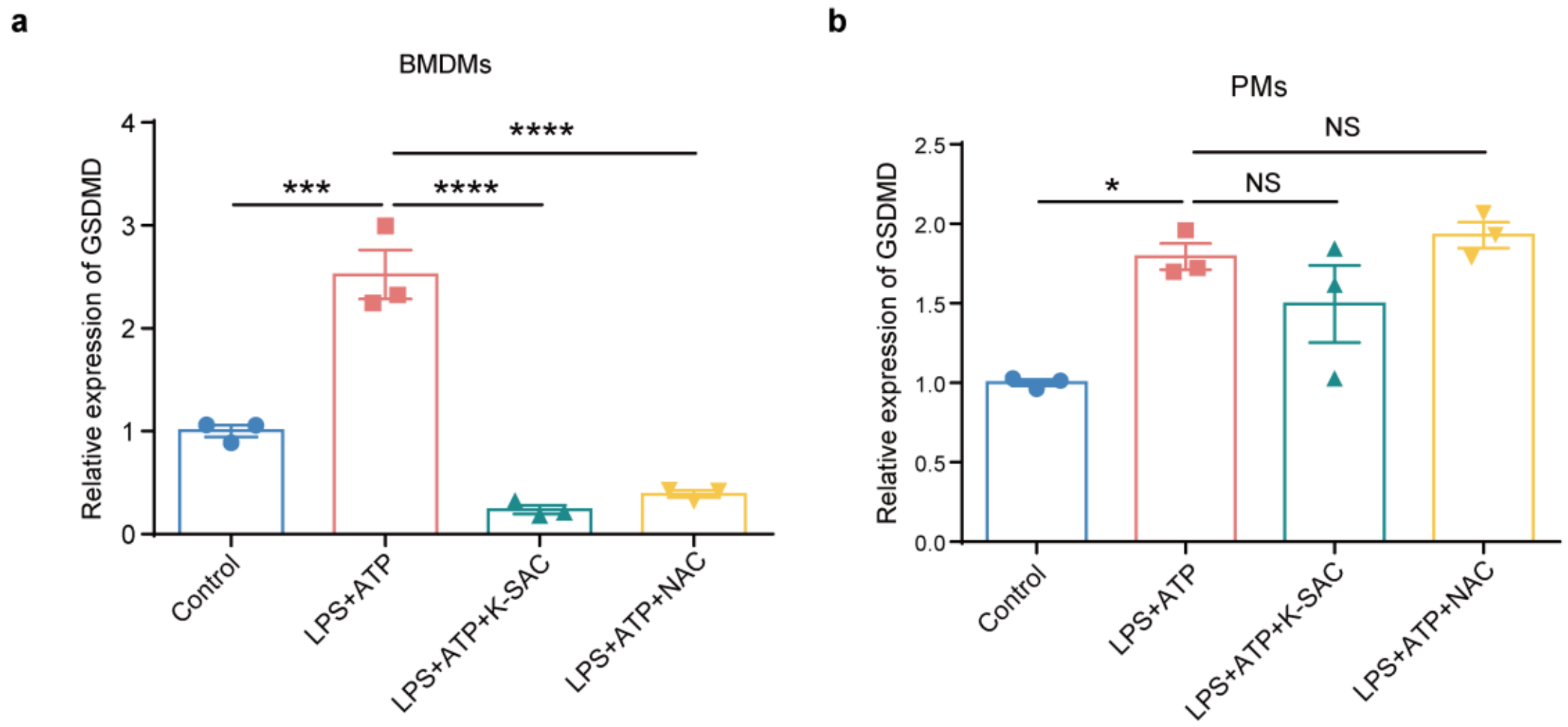

Supplementary Fig. 24. The expressions of GSDMD.

a,b, mRNA levels of GSDMD in BMDMs **(a)** and PMs **(b)** after K-SAC or NAC treatment and LPS and ATP stimulation. The results are presented as mean ± SD with $n = 3$ independent samples for each group. Statistical significance was calculated via one-way ANOVA, Tukey's multiple comparisons. Source data are provided as a Source Data file.

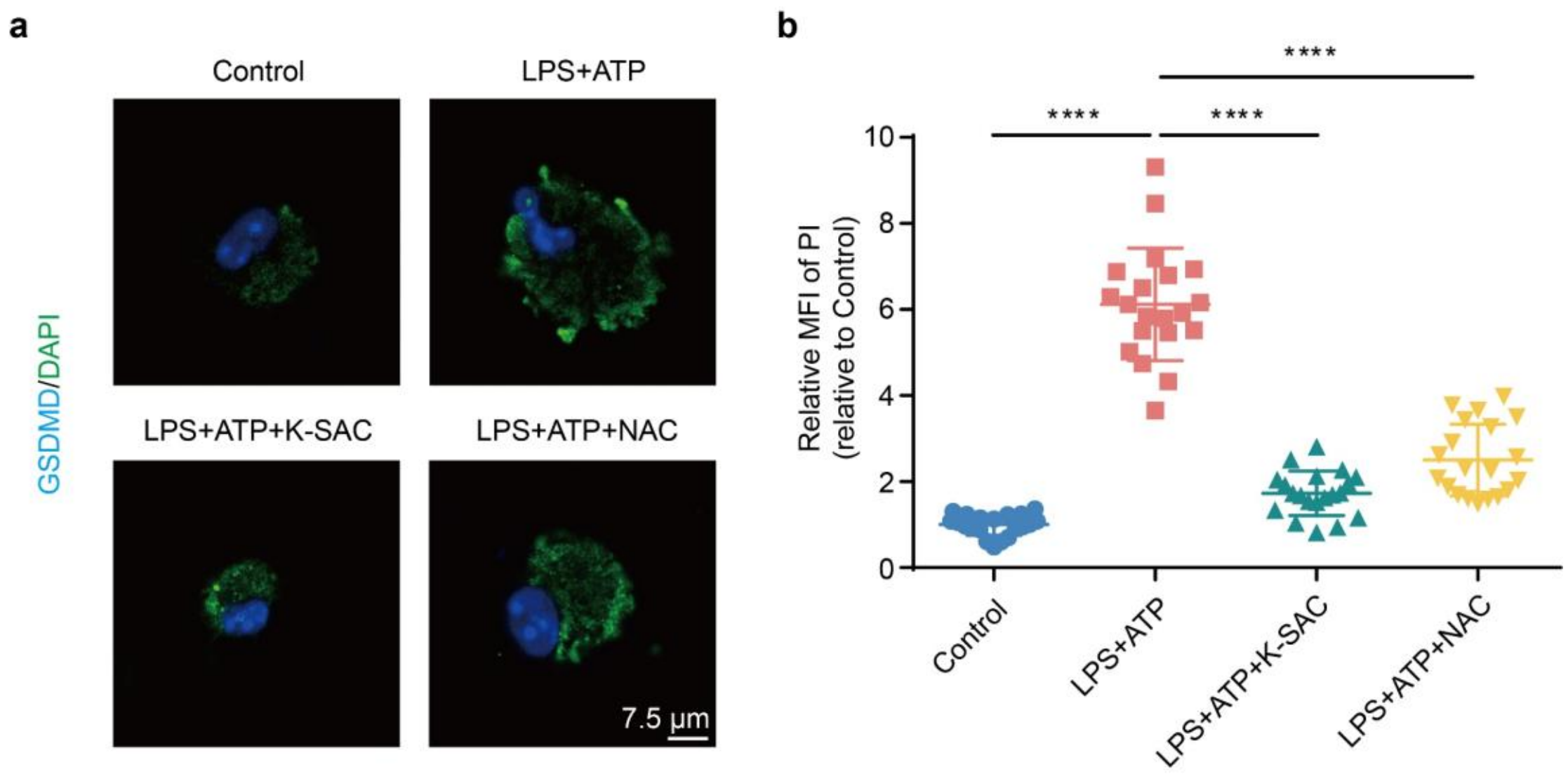

Supplementary Fig. 25. Representative images of GSDMD.

a,b, Representative images of immunofluorescent staining of GSDMD and DAPI in each group **(a)** and MFI of GSDMD **(b)** (Scale bar: 7.5 μm). Data are presented as mean ± SD. Statistical significance was calculated via one-way ANOVA, Tukey's multiple comparisons. Source data are provided as a Source Data file.

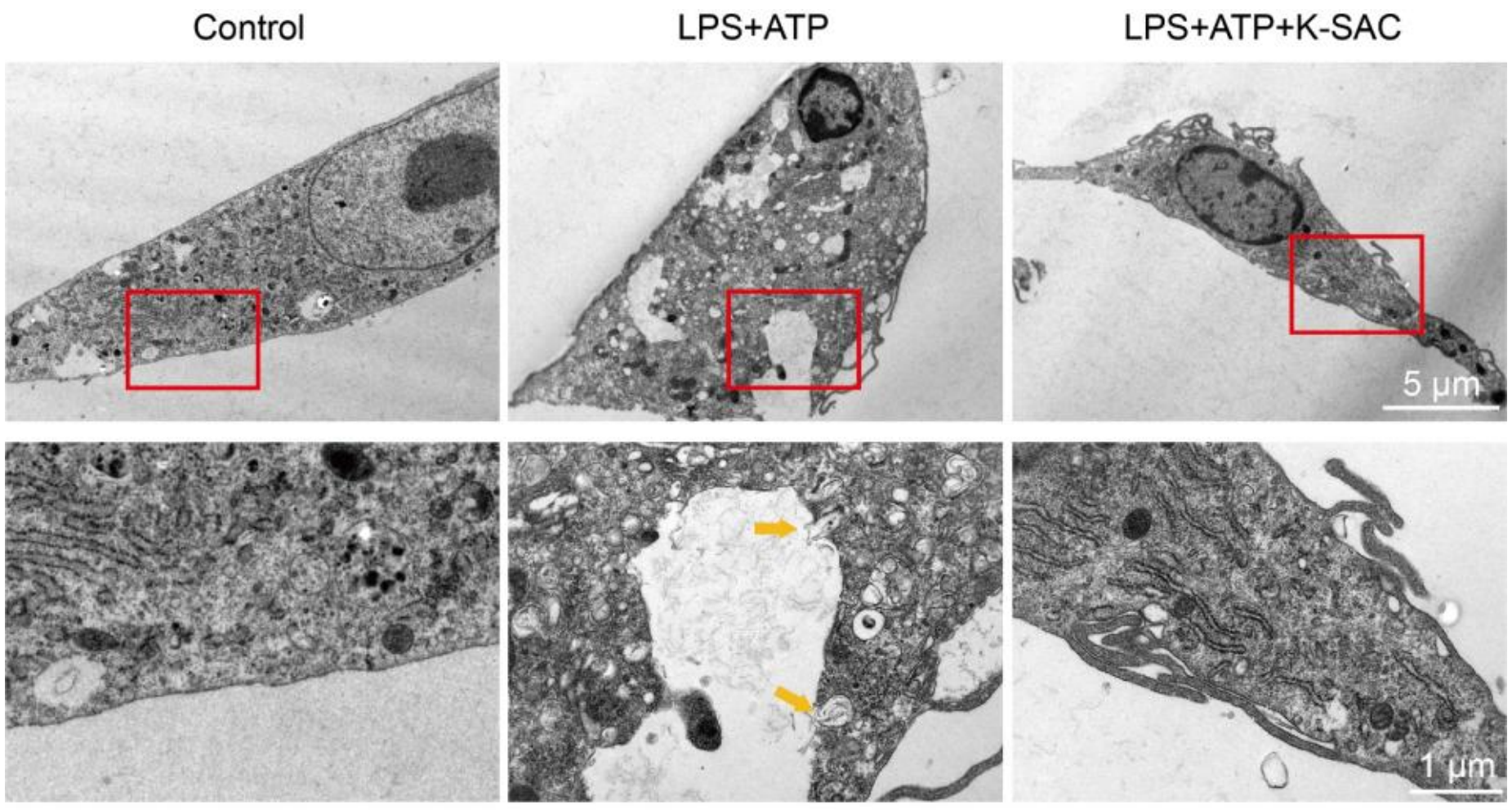

Supplementary Fig. 26. The TEM images of PMs.

Representative TEM images of PMs showing LPS/ATP-induced membrane damage (yellow arrows). (Scale bar, upper: 5 μm, lower: 1 μm).

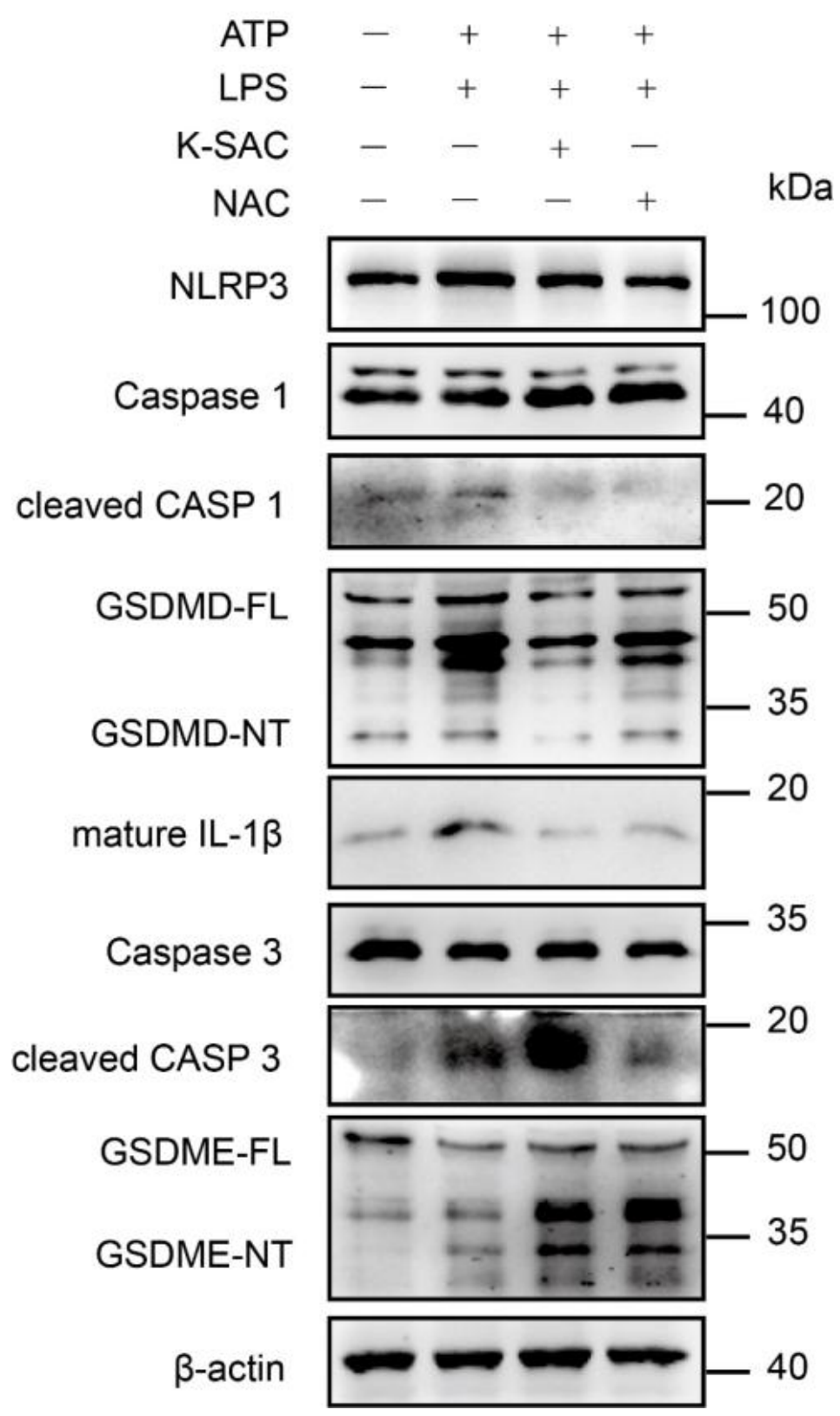

Supplementary Fig. 27. Representative western blot images of pyroptosis in PMs.

Western blot analysis of NLRP3, Caspase-1, cleaved Caspase-1, GSDMD, mature IL-1β, Caspase-3, cleaved Caspase-3, GSDME and β-actin protein levels in PMs. Source data are provided as a Source Data file.

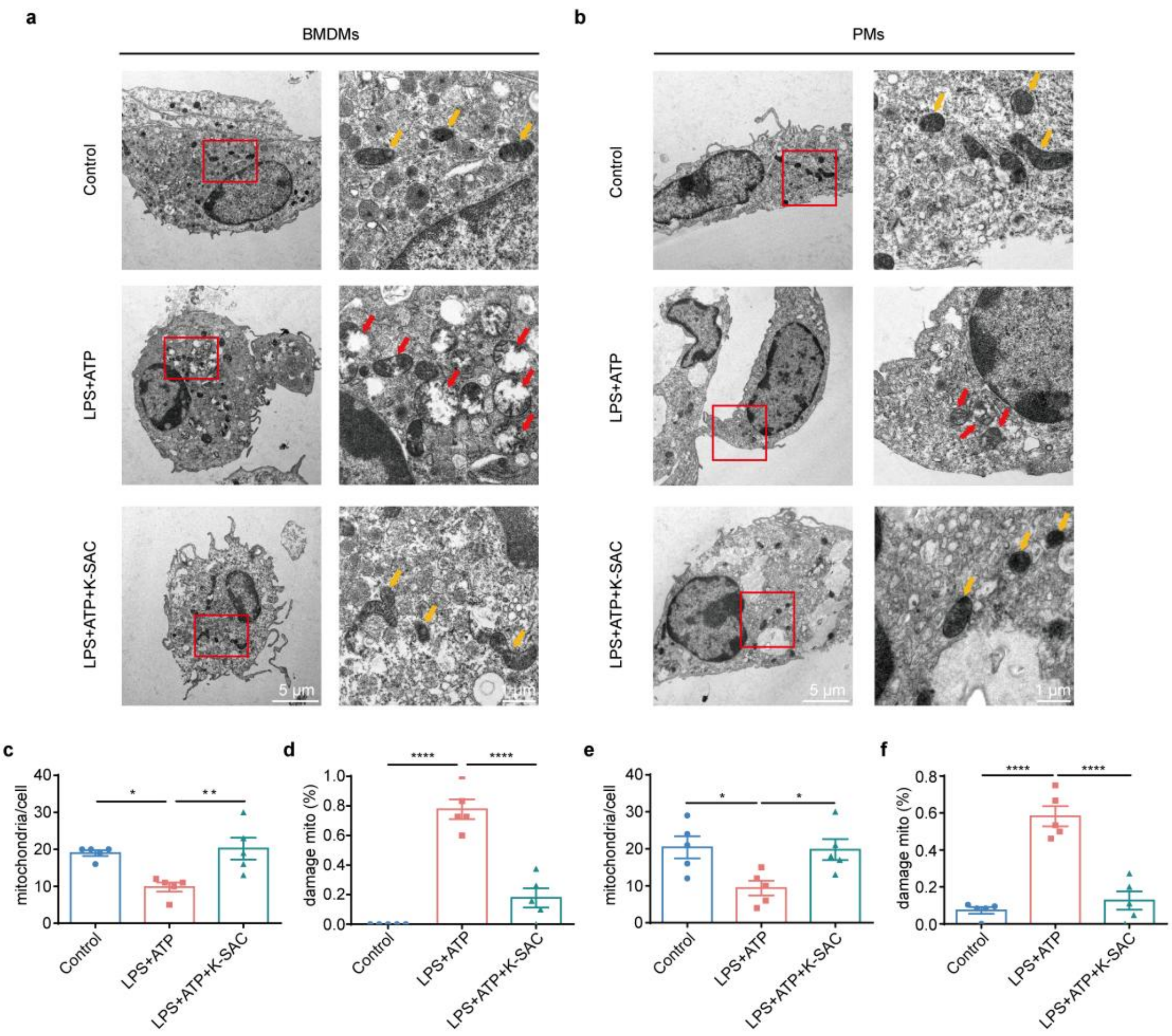

Supplementary Fig. 28. K-SAC alleviates mitochondria damage.

a,b, Representative images of TEM analysis of BMDMs **(a)** and PMs **(b)**. The right panels show higher magnification of the boxed area in the left panels. The yellow arrows pointed the normal mitochondria; red arrows pointed swelling mitochondria (Scale bar, upper: 5 μm, lower: 1 μm). **c-f,** Mitochondrial (mito) number and percentage of damaged mitochondria of BMDMs **(c,d)** and PMs **(e,f)** were quantified. Data are presented as mean±SD. Statistical significance was calculated via one-way ANOVA, Tukey's multiple comparisons. Source data are provided as a Source Data file.

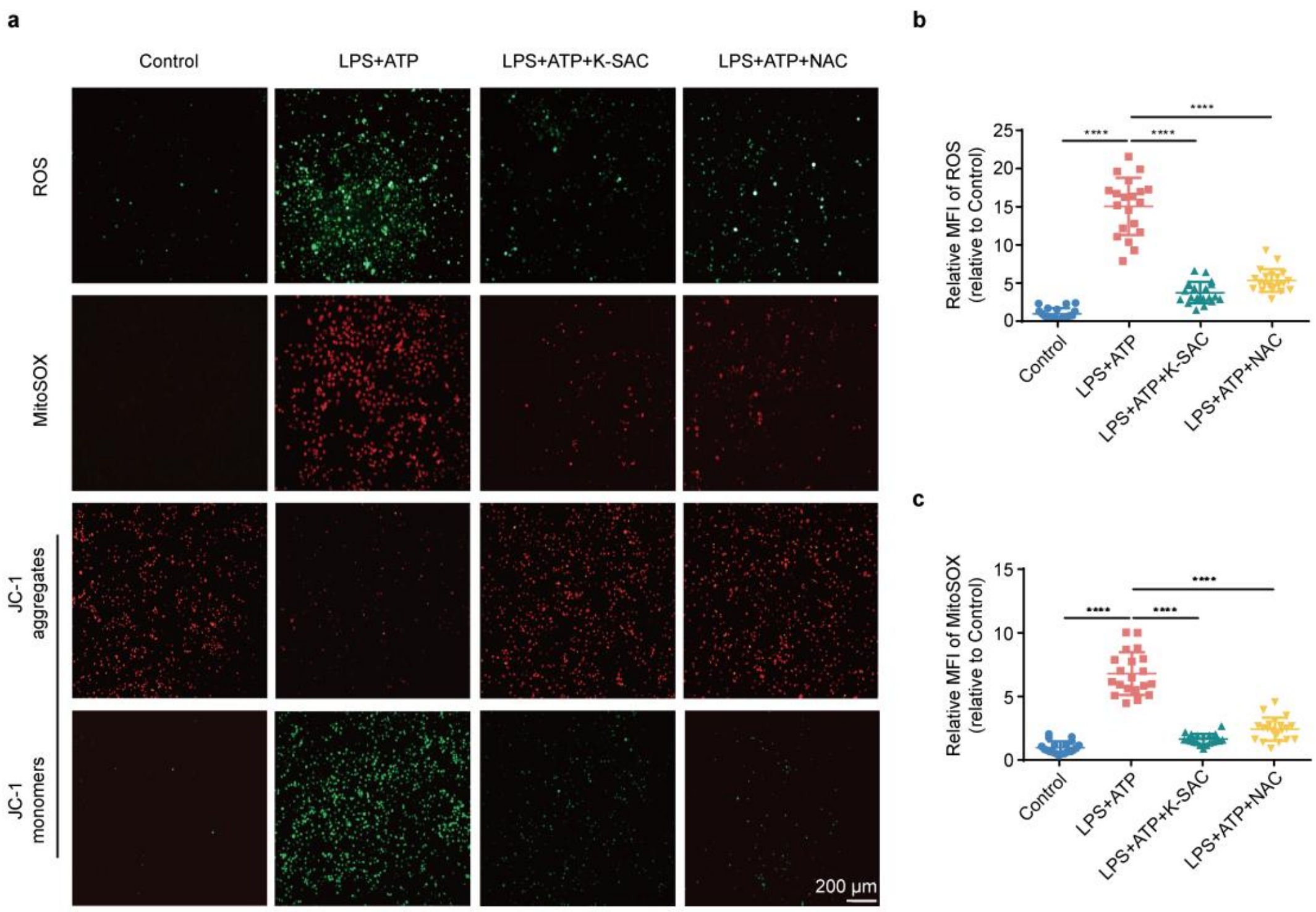

Supplementary Fig. 29. K-SAC scavenges ROS and restore mitochondrial membrane potential.

a, Representative fluorescence images of ROS, mitoSOX and JC-1 staining in different group of PMs (Scale bar, 200 μm). **b,c,** MFI of ROS **(b)** and mitoSOX **(c)**. Data are presented as mean±SD. Statistical significance was calculated via one-way ANOVA, Tukey's multiple comparisons. Source data are provided as a Source Data file.

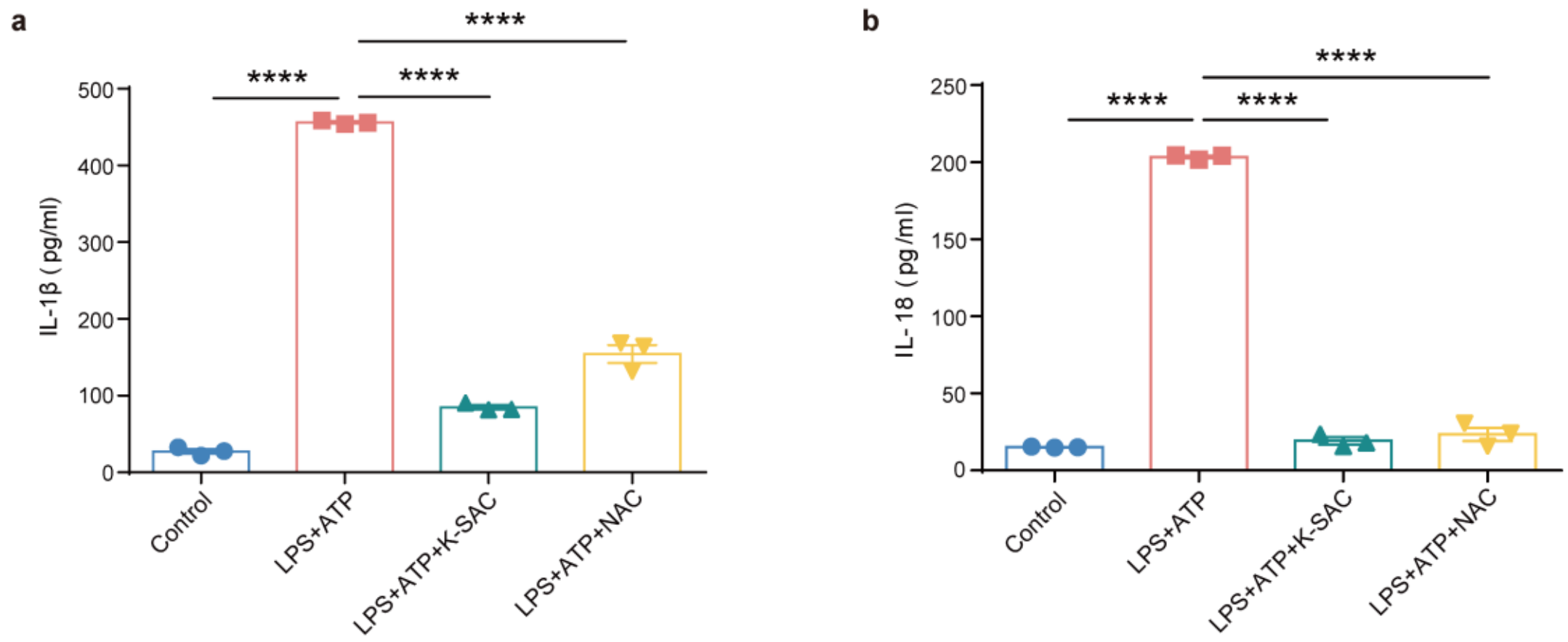

Supplementary Fig. 30. Anti-inflammation effects of K-SAC in PMs.

a,b, Expressions of IL-1β **(a)** and IL-18 **(b)** in the supernatants of PMs. Data are presented as mean ±SD. Statistical significance was calculated via one-way ANOVA, Tukey's multiple comparisons. Source data are provided as a Source Data file.

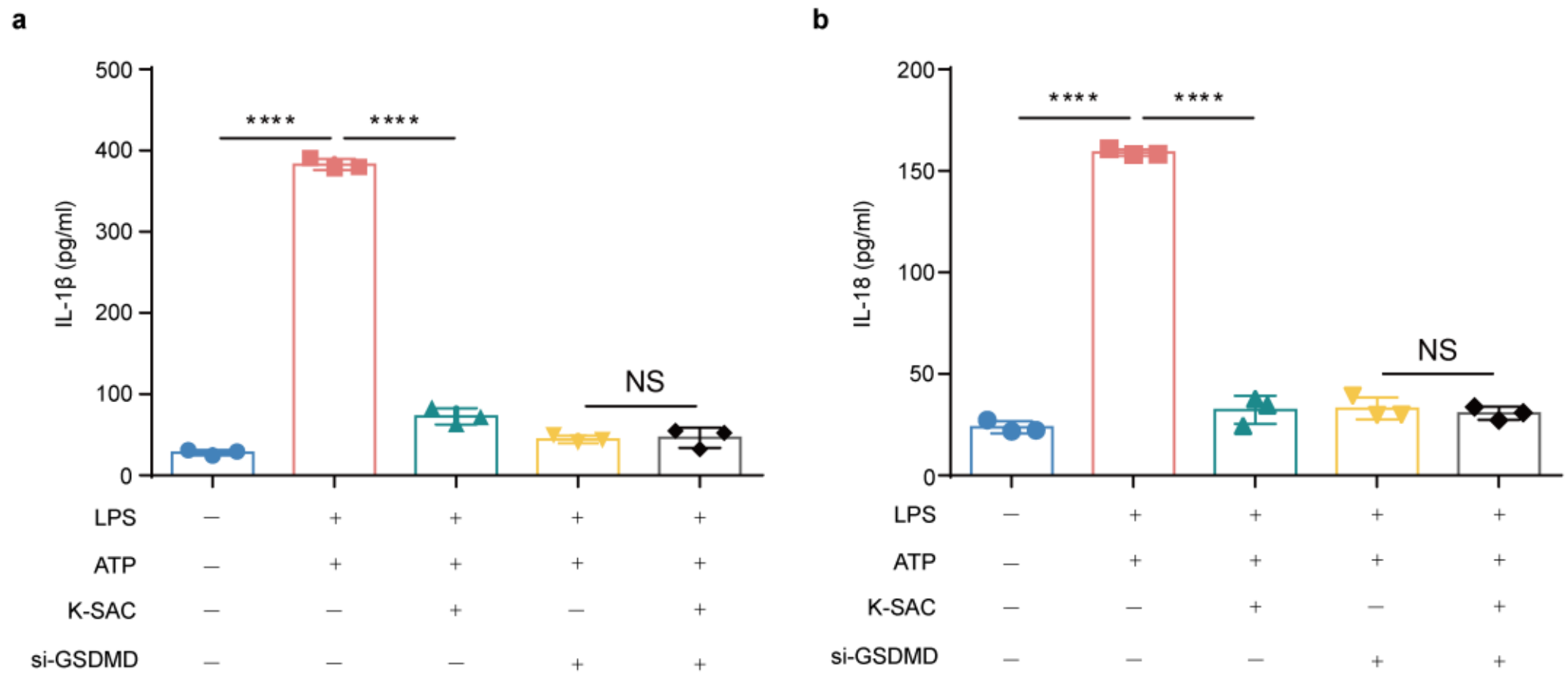

Supplementary Fig. 31. Anti-inflammation effects of K-SAC are GSDMD-dependent.

IL-1β **(a)** and IL-18 **(b)** levels in PMs supernatants treated with K-SAC, LPS and ATP, with or without GSDMD siRNA intervention. Data are presented as mean±SD. Statistical significance was calculated via one-way ANOVA, Tukey's multiple comparisons. Source data are provided as a Source Data file.

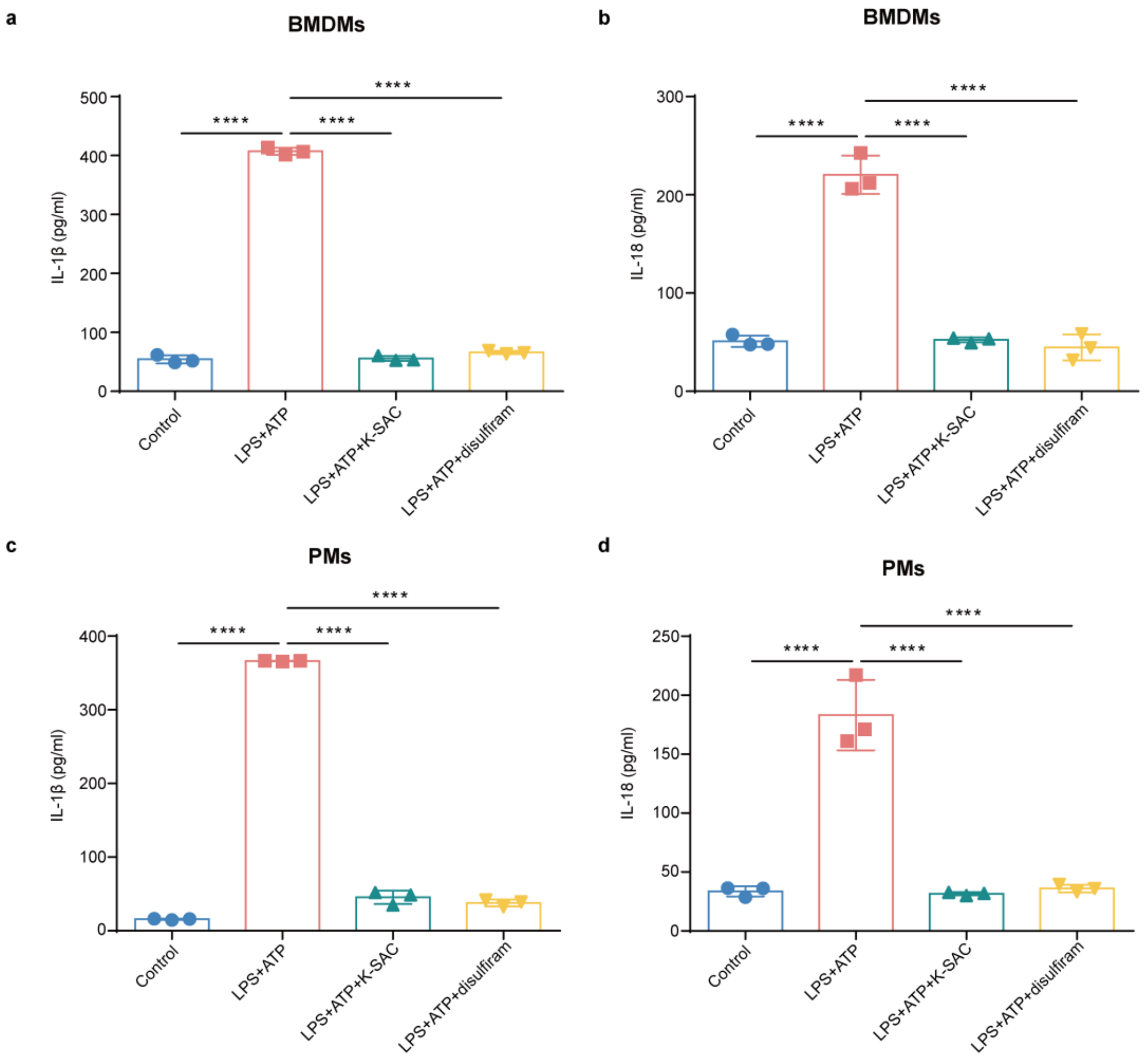

Supplementary Fig. 32. The pyroptosis inhibition efficacy of K-SAC compared with disulfiram.

a,b, IL-1β **(a)** and IL-18 **(b)** levels in BMDMs supernatants treated with K-SAC or disulfiram. **c,d,** IL-1β **(c)** and IL-18 **(d)** levels in PMs supernatants treated with K-SAC or disulfiram. Data are presented as mean ± SD. Statistical significance was calculated via one-way ANOVA, Tukey's multiple comparisons. Source data are provided as a Source Data file.

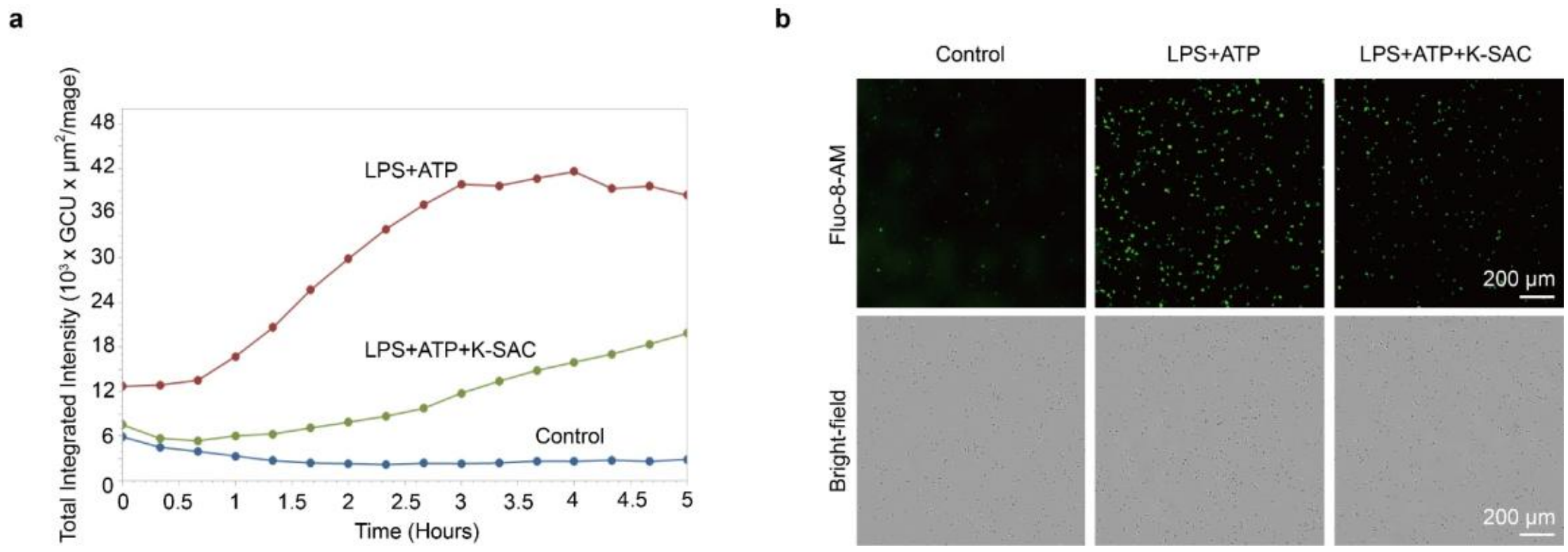

Supplementary Fig. 33. K-SAC alleviates cell membrane damage in PMs.

a, Total integrated intensity of Fluo-8-AM in PMs after LPS and ATP stimulation. **b,** Represent ative immunofluorescent images of Fluo-8-AM in PMs stimulated with LPS and ATP for 5 h (u pper) and corresponding bright-field images (lower) (Scale bar, 200 μm).

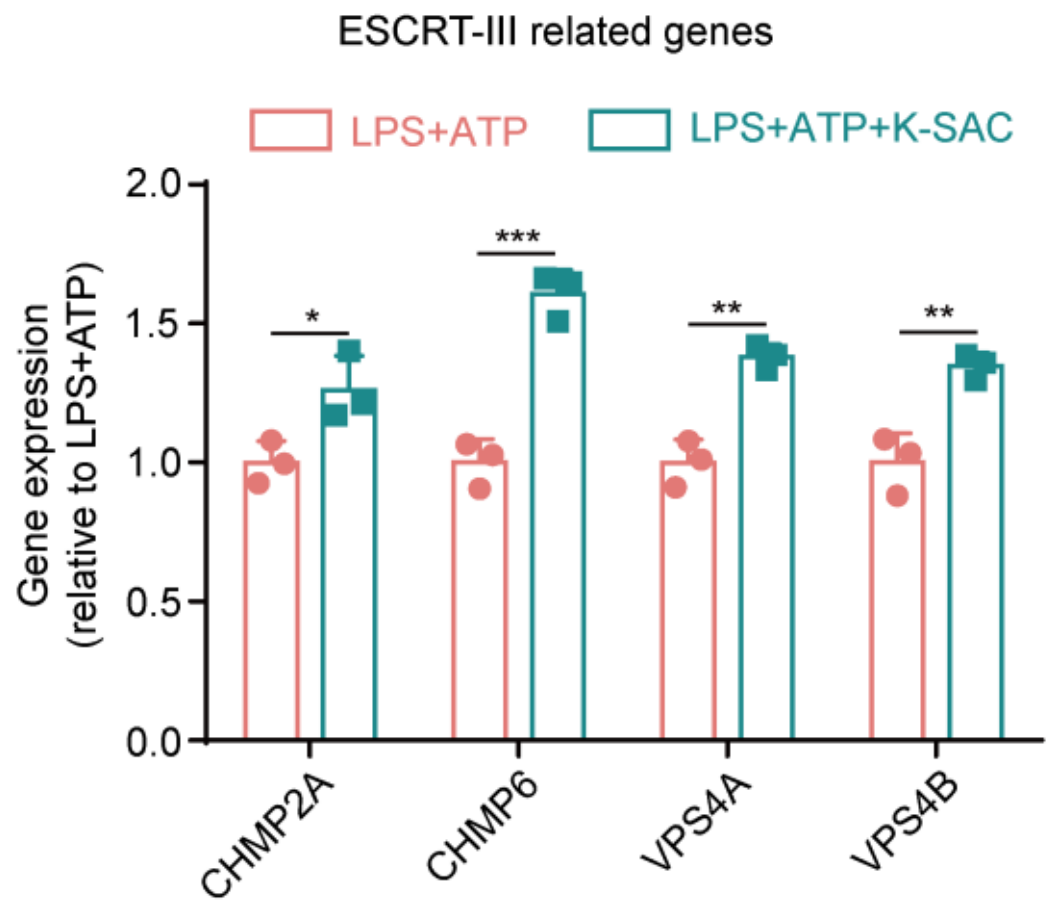

Supplementary Fig. 34. The changes of critical genes in ESCRT system after K-SAC treatment in PMs.

Data are presented as mean±SD. Statistical significance was calculated via unpaired t-test, two-tailed. Source data are provided as a Source Data file.

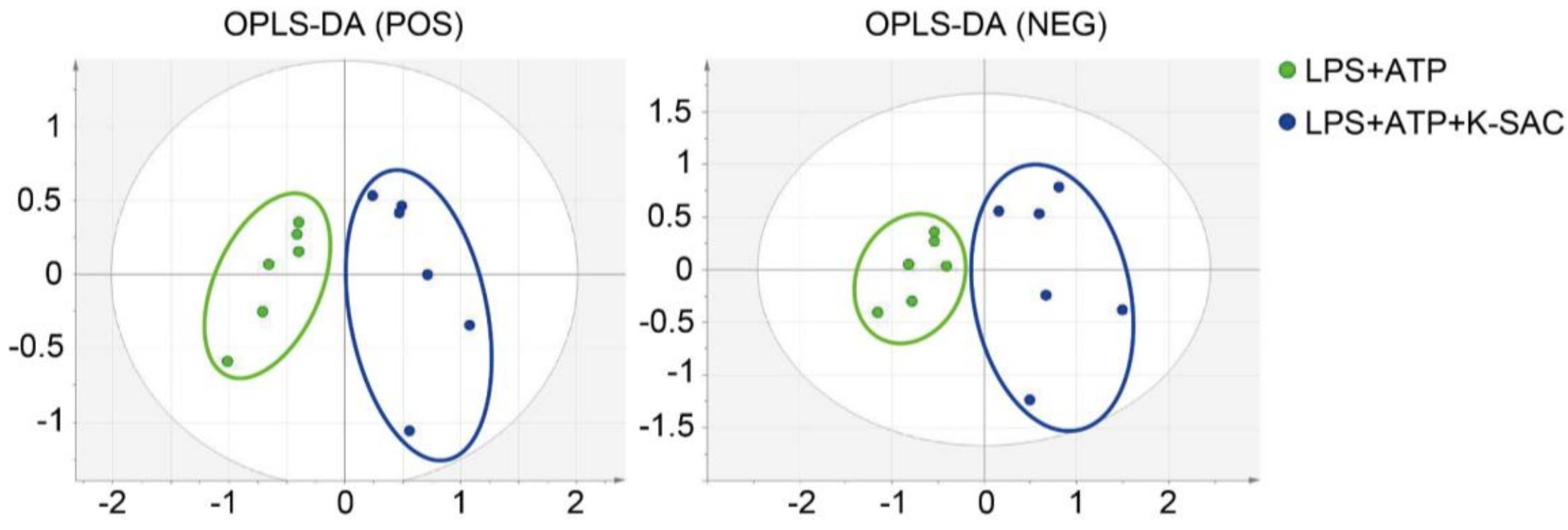

Supplementary Fig. 35. The OPLS-DA analysis of lipidomics analysis.

OPLS-DA score plot showing distinct clustering of lipid profiles between LPS+ATP treated BMDMs (e.g., green circles) and LPS+ATP+K-SAC treated BMDMs (e.g., blue circles).

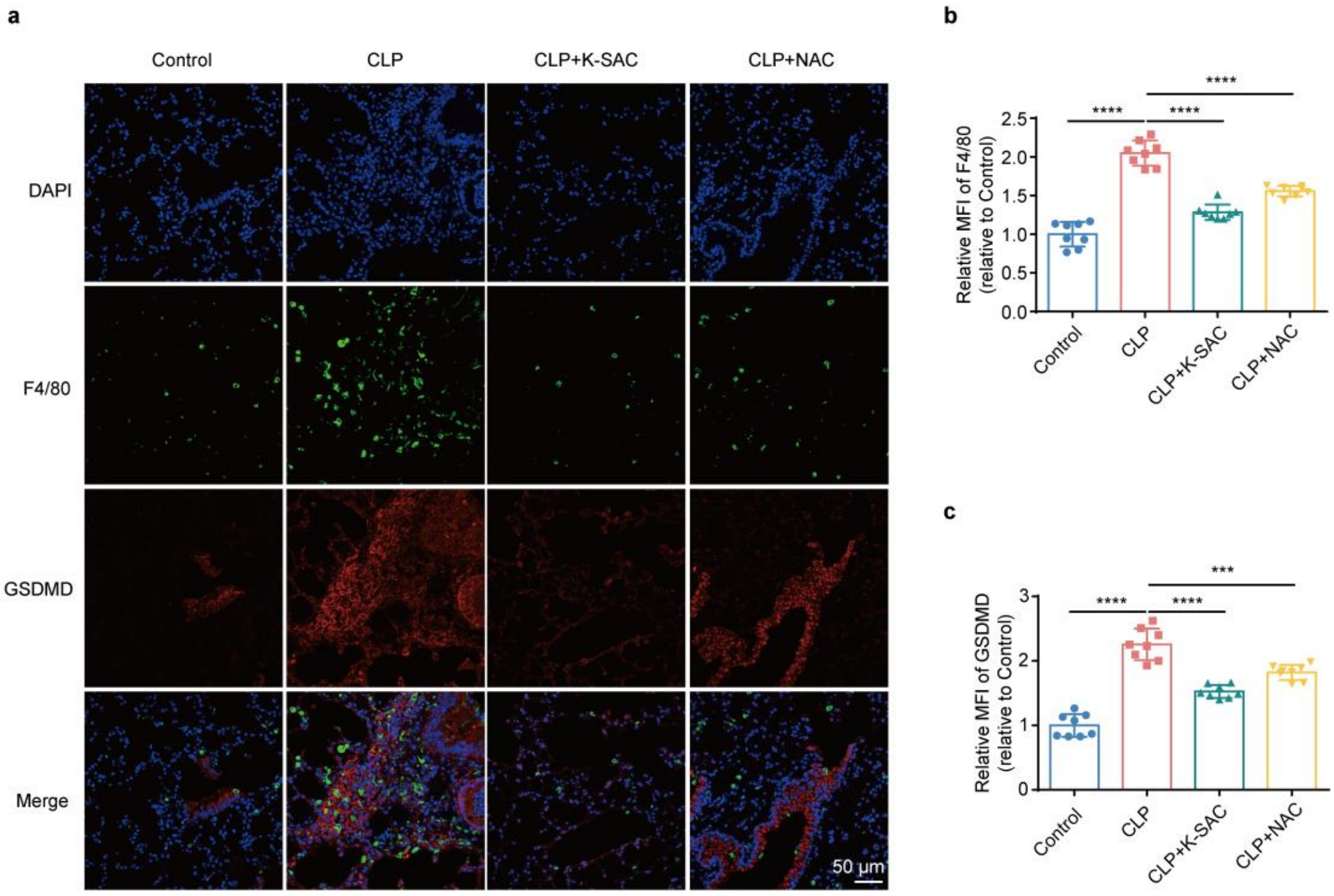

Supplementary Fig. 36. K-SAC reduces GSDMD expression in macrophages of sepsis-induced ALI.

a-c, Representative F4/80 immunofluorescent staining in lung sections **(a)** with quantitative analysis of F4/80 **(b)** and GSDMD **(c)** expression (Scale bar: 50 μm). The results are presented as mean ±SD with *n* = 8 independent samples for each group. Statistical significance was calculated via one-way ANOVA, Tukey's multiple comparisons. Source data are provided as a Source Data file.

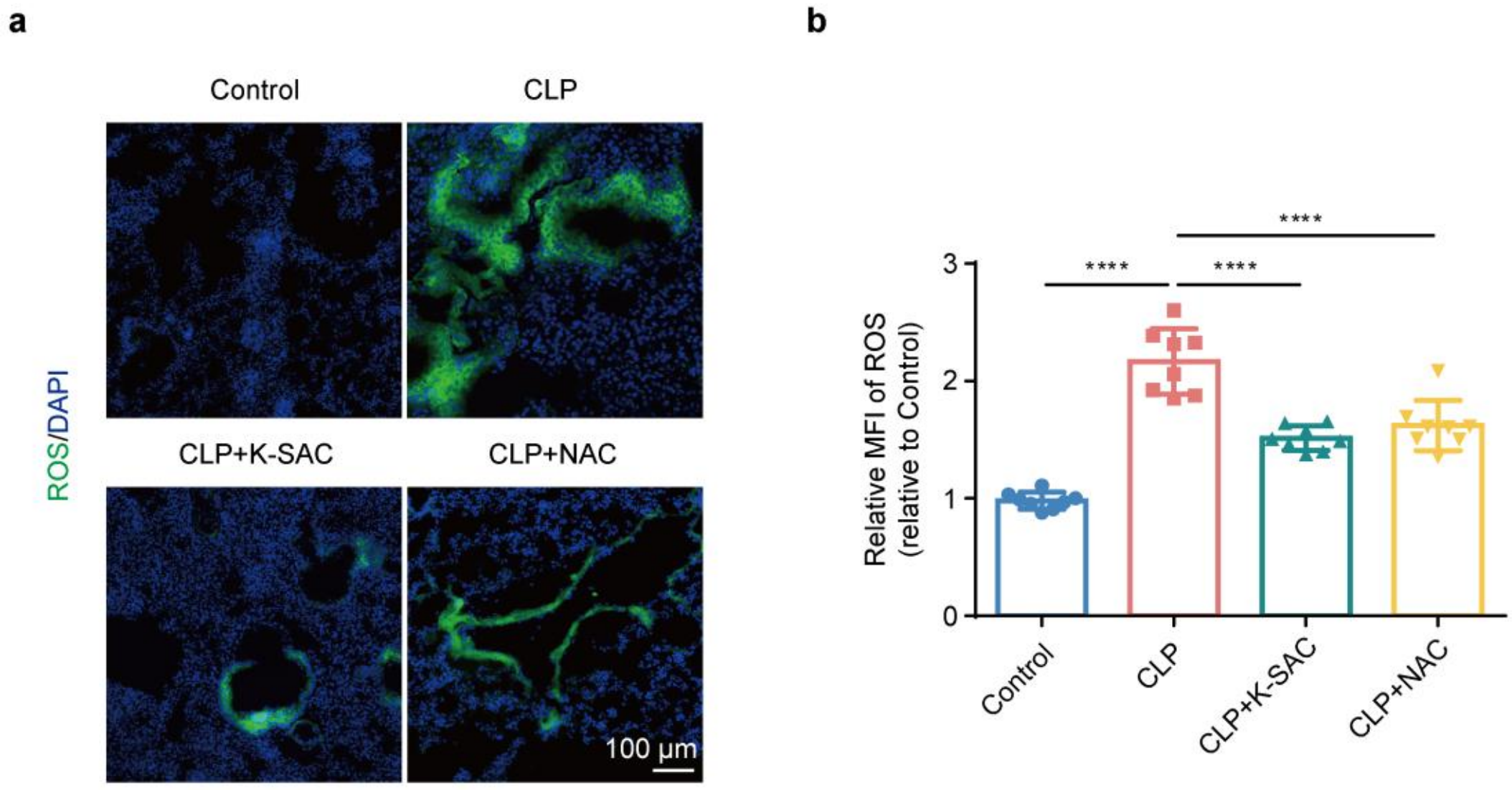

Supplementary Fig. 37. K-SAC scavenges ROS in sepsis-induced ALI.

Representative ROS staining images in lung sections **(a)** and the statistical analysis of ROS staining intensity **(b)** (Scale bar, 100 µm). The results are presented as mean ±SD with $n = 8$ independent samples for each group. Statistical significance was calculated via one-way ANOVA, Tukey's multiple comparisons. Source data are provided as a Source Data file.

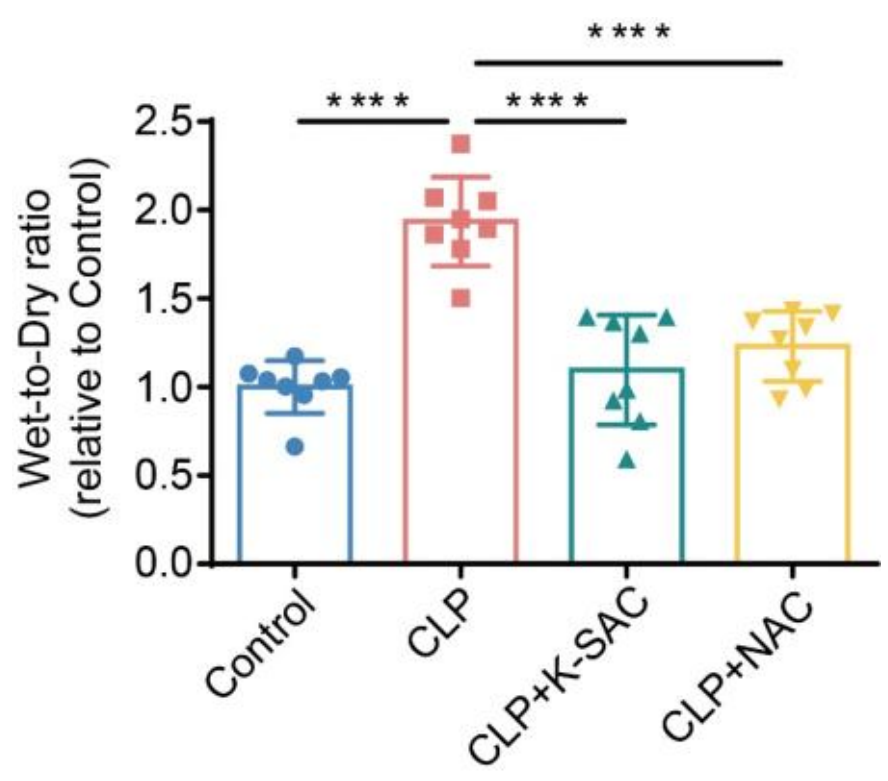

Supplementary Fig. 38. The lung W/D weight ratio of sepsis-induced ALI.

Data are presented as mean ± SD. Statistical significance was calculated via one-way ANOVA, Tukey's multiple comparisons. Source data are provided as a Source Data file.

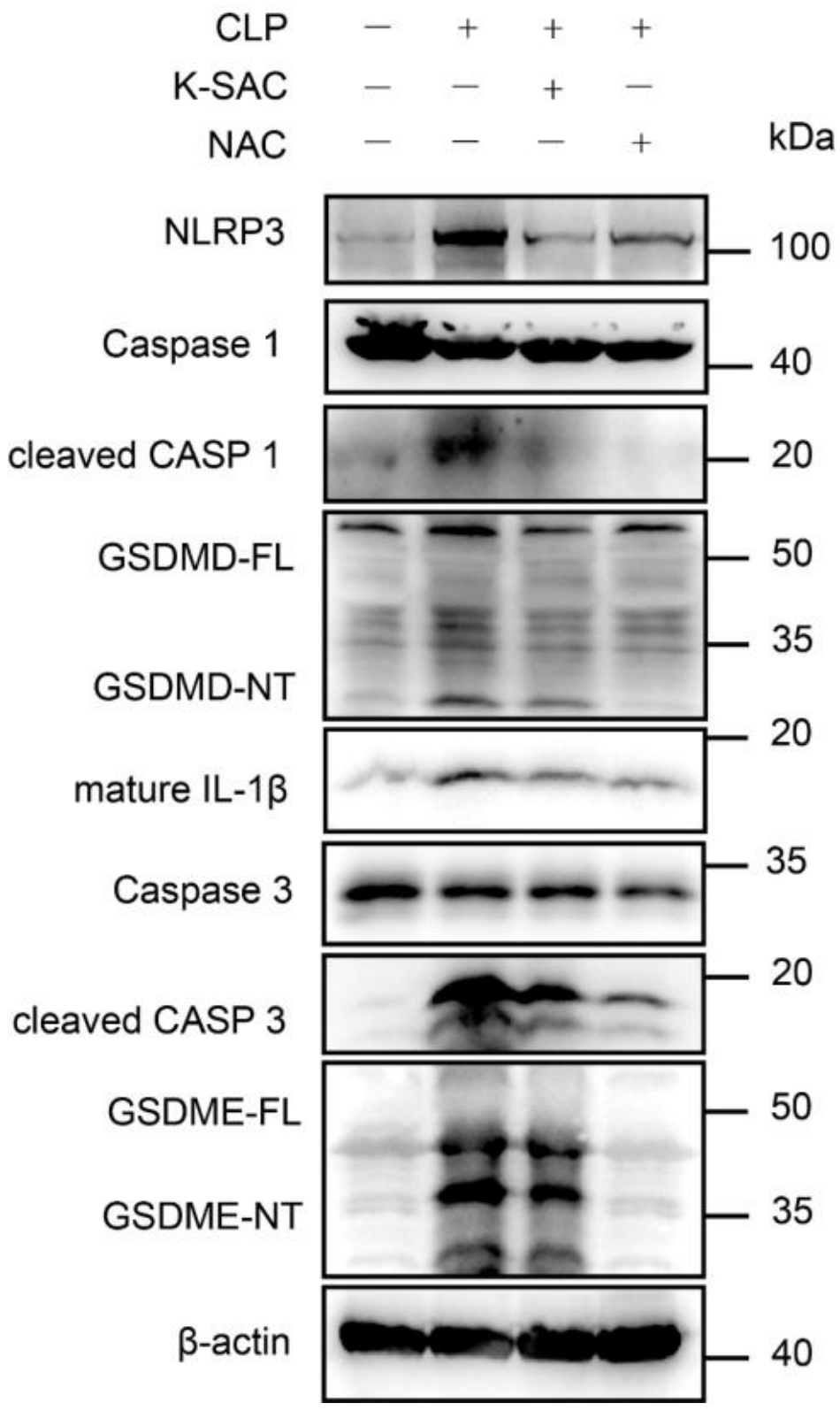

Supplementary Fig. 39. Representative Western blot images of pyroptosis in sepsis-induced ALI.

Western blot analysis of NLRP3, Caspase-1, cleaved Caspase-1, GSDMD, mature IL-1β, Caspase-3, cleaved Caspase-3, GSDME and β-actin protein levels in lung tissues from sepsis-induced ALI mice. Source data are provided as a Source Data file.

Supplementary Tables

Supplementary Table 1. EXAFS data fitting results of Samples.

<table>
<tr><th>Sample</th><th>Path</th><th>CN^a</th><th>R(Å)^b</th><th>σ^2 (Å^2)^c</th><th>ΔE_0(eV)^d</th><th>R factor</th></tr>
<tr><td rowspan="2">KCl</td><td>K-Cl</td><td>6 (set)</td><td>3.125±0.038</td><td>0.010</td><td rowspan="2">-0.06</td><td rowspan="2">0.0127</td></tr>
<tr><td>K-K</td><td>12 (set)</td><td>4.481±0.008</td><td>0.099</td></tr>
<tr><td rowspan="2">K-SAC</td><td>K-N</td><td>3.8±0.1</td><td>2.654±0.066</td><td>0.018</td><td rowspan="2">-4.6</td><td rowspan="2">0.0020</td></tr>
<tr><td>K-O</td><td>3.6±0.4</td><td>2.719±0.074</td><td>0.018</td></tr>
</table>

[a]*CN*, coordination number; [b]*R*, the distance between absorber and backscatter atoms; [c]σ^2, the Debye Waller factor value; [d]ΔE_0, inner potential correction to account for the difference in the inner potential between the sample and the reference compound; *R* factor indicates the goodness of the fit. S_0^2 was fixed to 0.96, according to the experimental EXAFS fit of KCl by fixing *CN* as the known crystallographic value. Fitting conditions: *k* range: 3 – 8.3 Å^{-1}; *R* range: 1.0 – 3.0 Å; fitting space: R space; *k*-weight = 2. A reasonable range of EXAFS fitting parameters: $0.800 < S_0^2 < 1.000$; $CN > 0$; $\sigma^2 > 0$ Å^2; $|\Delta E_0| < 10$ eV; *R* factor < 0.02.

Supplementary Table 2. The proportion of different nitrogen species for the two catalysts from XPS results.

Sample	oxidized N	graphitic N	pyrrolic N	pyridinic N
Peak position (eV)	402.7	401.0	399.1	398.3
NC	21.6	36.8	25.7	15.9
K-SAC	21.2	35.9	23.7	19.2

Supplementary Table 3. Sequences of the primers used for RT-qPCR

Serial number	Primer name	Gene ID	Forward sequences	Reverse sequences
1	Mouse CHMP2A	68953	AGACGCCAGAGGA ACTACTTC	ACCAGGTCTTTTGCCATG ATTC
2	Mouse CHMP6	208092	CAACTGCTCGATAG GACAGAAAA	CAGACATTCGTTCCCCAC CTG
3	Mouse VPS4A	116733	GATCTGGTGACAAA AGCCACA	CTTTGCTCGAATGCTCTC CTT
4	Mouse VPS4B	20479	TGGGAACTATGAGG AAGCTCTT	ACTTTGCTTGGCTTTATC ACCTT
5	Mouse GSDMD	69146	CCATCGGCCTTTGA GAAAGTG	ACACATGAATAACGGGG TTTCC

Supplementary Table 4. Sequences of the small interfering RNAs targeting GSDMD

Serial number	Primer name	Gene ID	Sequence (5'-3')
1	Mouse GSDMD	69146	CGTTATTCATGTGTCAACCTGTCAA

Reference:

1. Kresse G, Furthmüller J. Efficiency of ab-initio total energy calculations for metals and semiconductors using a plane-wave basis set. *Computational Materials Science* **6**, 15-50 (1996).
2. Kresse G, Furthmüller J. Efficient iterative schemes for ab initio total-energy calculations using a plane-wave basis set. *Physical review B, Condensed matter* **54 16**, 11169-11186 (1996).
3. Perdew JP, Burke K, Ernzerhof M. Generalized Gradient Approximation Made Simple. *Physical review letters* **77 18**, 3865-3868 (1996).
4. Kresse G, Joubert D. From ultrasoft pseudopotentials to the projector augmented-wave method. *Physical Review B* **59**, 1758-1775 (1999).
5. Blöchl PE. Projector augmented-wave method. *Physical review B, Condensed matter* **50 24**, 17953-17979 (1994).
6. Grimme S, Antony J, Ehrlich S, Krieg H. A consistent and accurate ab initio parametrization of density functional dispersion correction (DFT-D) for the 94 elements H-Pu. *The Journal of Chemical Physics* **132**, 154104 (2010).
7. Young MD, Wakefield MJ, Smyth GK, Oshlack A. Gene ontology analysis for RNA-seq: accounting for selection bias. *Genome Biol* **11**, R14 (2010).
8. Mao X, Cai T, Olyarchuk JG, Wei L. Automated genome annotation and pathway identification using the KEGG Orthology (KO) as a controlled vocabulary. *Bioinformatics* **21**, 3787-3793 (2005).
9. Liu M*, et al.* α-Ketoglutarate Modulates Macrophage Polarization Through Regulation of PPARγ Transcription and mTORC1/p70S6K Pathway to Ameliorate ALI/ARDS. *Shock (Augusta, Ga)* **53**, 103-113 (2020).
