# Supplementary material for: A potassium-based single-atom catalyst enables acute lung injury immunotherapy in mice by inhibiting macrophage pyroptosis
Source: Nat Commun. 2026 Aug 4;17:9400. doi: 10.1038/s41467-026-76331-8 (PMC13534642; doi:10.1038/s41467-026-76331-8)
Supplement: Supplementary file 2 [file 41467_2026_76331_MOESM2_ESM.pdf]

Description of Additional Supplementary Files

Title: Supplementary Data 1

Description: The differentially expressed lipids after K-SAC treatment.
