# Supplementary material for: A potassium-based single-atom catalyst enables acute lung injury immunotherapy in mice by inhibiting macrophage pyroptosis
Source: Nat Commun. 2026 Aug 4;17:9400. doi: 10.1038/s41467-026-76331-8 (PMC13534642; doi:10.1038/s41467-026-76331-8)
Supplement: Supplementary file 4 [file 41467_2026_76331_MOESM5_ESM.pdf]

nature portfolio

Peer Review File

Potassium Single-Atom Catalyst Enables Acute Lung Injury Immunotherapy by Inhibiting Macrophage Pyroptosis

Corresponding Author: Professor Jianlin Shi

This file contains all reviewer reports in order by version, followed by all author rebuttals in order by version.

Version 0:

Reviewer comments:

Reviewer #1

(Remarks to the Author)
The manuscript by Xiangyu Lu and colleagues presents a novel therapeutic approach for acute lung injury (ALI) using potassium-based single-atom catalysts (K-SACs). The authors demonstrate that K-SACs exhibit dual SOD- and CAT-like enzymatic activities, enabling ROS scavenging and inhibition of pyroptosis. The work is well-executed with comprehensive characterization of the nanomaterial and mechanistic investigations both in vitro and in vivo. The concept of using s-block metal single-atom catalysts represents an innovative approach to address metal toxicity concerns associated with transition metal-based therapeutics.
The use of potassium as the catalytic center is innovative and addresses biocompatibility concerns inherent to transition metal catalysts. The structural and catalytic properties of K-SACs are thoroughly characterized using relevant techniques (DLS, HAADF-STEM, EXAFS, DFT calculations, etc.). The study provides detailed mechanistic insights into pyroptosis inhibition, including ROS scavenging, GSDMD downregulation, ESCRT-mediated membrane repair, and phospholipid remodeling. The validation in both LPS-induced and CLP-induced ALI models strengthens the therapeutic relevance. The manuscript is generally well-written with logical flow and appropriate use of figures.
However, some flaws need to be addressed to improve the quality of the manuscript.
Major Concerns
1. The immunofluorescence images in Figure 6a showing F4/80 and GSDMD co-localization are not convincing. The GSDMD signal appears to follow an organized tissue pattern more consistent with epithelial cells (bronchial and alveolar) rather than infiltrating macrophages, as GSDMD is abundantly expressed in airway epithelium (as seen in Figure 1B from 10.1038/s41419-023-06258-1). The yellow co-localization is visible in only a few cells, raising concerns about the conclusion that K-SACs primarily target macrophage pyroptosis. Co-staining with epithelial markers (e.g., E-cadherin, EpCAM) is essential to confirm cell-type-specific GSDMD expression. Additionally, in vitro co-culture experiments with macrophages and epithelial cells would clarify which cell type is primarily affected. The conclusions should be revised to acknowledge potential epithelial cell effects. While the authors demonstrate high macrophage accumulation in vitro, the mechanism driving macrophage selectivity is not explored.

2. K-SACs may affect multiple cell types beyond macrophages (neutrophils, epithelial cells, endothelial cells). The manuscript focuses exclusively on macrophages without exploring broader cellular effects. Assessing K-SACs uptake and effects in other relevant lung cell types, particularly neutrophils given their role in ALI pathogenesis and epithelial cells, would strengthen the work and clarify the role of K-SAC in ALI.

3. While the authors demonstrate that K-SACs reduce GSDMD expression and pyroptotic markers, they do not directly prove that pyroptosis inhibition is GSDMD-dependent. The stated conclusion that K-SACs work "by inhibiting macrophage pyroptosis" would be strengthened by loss-of-function experiments. Including experiments with GSDMD- or ESCRT-deficient macrophages (knockout or siRNA knockdown) treated with K-SACs would be valuable. If pyroptosis inhibition persists in GSDMD- or ESCRT-null cells, alternative mechanisms should be investigated. This would definitively establish whether GSDMD is the primary target.

4. The methods describe three K-SACs administration regimens (48h, 24h, and simultaneous with LPS), but the results section does not clarify which protocol was used for Figure 6, nor are comparative data presented. Understanding the therapeutic window is critical for clinical translation.

Minor Concerns
5. BMDMs and PMs exhibit different phenotypic profiles and responses to stimulation, as evident in Supplementary Figure 21 showing differential GSDMD expression. These cell-type-specific differences are not addressed in the discussion. Including a brief discussion on how macrophage heterogeneity might influence K-SACs efficacy and whether targeting specific macrophage subsets would be advantageous could strengthen the manuscript.

6. Several figure legends lack essential information. Some examples:
o Figure 3e,f: NAC concentration not specified
o Figure 4: Drug concentrations and incubation times inconsistently reported
o Figure S25: Figure lettering is incorrect from b-e; f is missing
o The abbreviation OPLS-DA in Supplementary Fig. 30 is not described in the text (not even in the methodological section)
Systematically review all figure legends to include drug concentrations (such as for CeO_2 and Mn_3O_4), incubation times, and sample sizes. Ensure methods are fully detailed. Figures 5f-g present phospholipid composition changes but do not clearly state whether the percentages represent treated (LPS+ATP+K-SAC) vs. control or just treated samples. Presenting side-by-side comparisons showing lipid composition in control, LPS+ATP, and LPS+ATP+K-SAC groups or clarifying the data would help to understand the experiment. Include statistical analysis of compositional changes.
7. Some supplementary figures and tables are not referenced in numerical order (e.g., Fig. S4 before S3). Supplementary Figures 8 and 11 are not cited in the main text.

8. Figure legends describe using one-way ANOVA with Tukey post-hoc test for multiple comparisons, but sample sizes vary (n=3 to n=8). Power analysis is not mentioned. Provide justification for sample sizes and confirm adequate statistical power.

9. All in vivo experiments use a single dose (1 mg/kg). No dose optimization or dose-response relationship is established. Explain the rationale for the selection of this dose.

10. Biocompatibility is assessed only acutely. Chronic effects, tissue accumulation, and long-term outcomes are not evaluated. Conducting extended safety studies (2-4 weeks) monitoring organ function, biodistribution, and clearance kinetics, as well as assessing whether repeated dosing maintains efficacy without toxicity, would be very valuable for clinical translation. At a minimum, the acknowledgment of the need to conduct these studies should be mentioned in the manuscript. Similarly, practical considerations for human translation (manufacturing scalability, administration route optimization, regulatory pathway) are not discussed. Including a brief discussion on translational challenges and the proposed development pathway toward clinical application could be valuable for the study.

11. NAC is used as a positive control, but it is primarily an antioxidant rather than a specific pyroptosis inhibitor. Comparison with known pyroptosis inhibitors (e.g., disulfiram, necrosulfonamide) would better contextualize K-SACs' efficacy.

12. Some references lack complete information (page numbers).
In summary, this manuscript presents innovative and promising work on K-SACs for ALI treatment. The catalyst design is novel, characterization is comprehensive, and mechanistic insights are valuable. However, several critical experiments are needed to fully validate the proposed mechanism, particularly regarding GSDMD-dependency and cell-type specificity. Addressing the major concerns, especially validation of macrophage-specific GSDMD targeting and dose-response/temporal analyses, would significantly strengthen the manuscript. With appropriate revisions, this work has strong potential for publication and could make an important contribution to the field of nanomedicine-based immunotherapy.

Reviewer #2

(Remarks to the Author)
This manuscript presents an interesting study proposing potassium single-atom catalyst as a new strategy to inhibit macrophage pyroptosis in acute lung injury. The work is notable for using a safe, physiologically abundant metal that exhibits antioxidant activity, highlighting its potential for further therapeutic development. While the study is promising, I have several specific comments and suggestions that could help improve the clarify and presentation of the manuscript.
1. In the manuscript, the authors propose a structural model of the K center in the K-SACs based on the EXAFS data fitting, followed by DFT optimization. In this model, K is coordinated by four N atoms and is displaced from the plane of the support. However, such a K site would be expected to adsorb gas molecule from the atmosphere (e.g. H2O, CO2, O2), which should influence the first coordination sphere of K in the EXAFS spectrum.
EXAFS fitting showed the first K-N and second K-C shell at 2.72 and 3.04 Å, respectively. Could the authors justify the possibility of forming a K-C second coordination at 3.04 Å? Because K has a large ionic radius, its first-shell bond distances can reach up to 3.10 Å. Considering that K-O and K-C scattering paths cannot be reliably distinguished by EXAFS, the authors could modify EXAFS fitting by replacement of the K-C path with a K-O, where a K-O path can be rationalized as the first coordination shell formed upon adsorption of atmospheric gas molecules on the isolated K site. This interpretation does not alter the overall structural model of the K single center proposed in the manuscript but provides a more chemically reasonable description of the local K environment in the real catalyst.
2. The author should provide additional details regarding the EXAFS fitting procedure, including the k- and r-ranges, the specific k-weight(s) used in the analysis, and the method used to determine the amplitude reduction factor (S02).
3. It is well known that K K-edge XANES spectra strongly depend on the K-containing phase. It would be valuable if the authors could provide a brief discussion of the obtained K XANES spectrum in comparison with literature data. Such an analysis would further support the absence of bulk K-containing phases (including highly dispersed species that may be undetectable by XRD) and strengthen the evidence for the formation of isolated K center in the catalyst.

4. The FTIR data (Sup. Fig.5) are not entire clear. First, it would be helpful if the author could provide the exact values of the C-N and C=N stretching modes and cite relevant literature where similar bands have been observed. Additionally, it is not clear what specific information this experiment is intendent to provide. For instance, should the introduction of K on the support alter the ratio of C-N to C=N bonds? A similar issue arises with the XPS data (lines 157-160 in the main text, Sup. Fig.7). Is it unclear what evidence indicates the formation of different types of N on the surface of K-SACs and NC, and how the introduction of K affects the distribution of these N species. Based on the data presented, it would be helpful if the authors could provide clearer conclusions drawn from these measurements.
5. There are several typos and inaccuracies throughout the manuscript:
• Line 99 "…potassium-based single-atom catalysts (K-SACs)..", but this study presents only one catalyst. The abbreviation "K-SACs" may be confusing, suggesting multiple catalysts were studied. It is suggested to change K-SAC.
• Line 119 "As a control, K-free NC was also prepared…", abbreviation NC is not clarified.
• Figures: The sequence and referencing of figures in the manuscript are inconsistent. For example, in the text, Supplementary Fig. 4 is cited before Supplementary Fig.3. Additionally, some figures are included in the Supplementary Information but are not mentioned in the main text, such as Supplementary Fig. 8 and 11.
• Figure 1 (line 496): The caption "XPS spectrum" should be corrected to "XP spectrum"; similar errors are present in other figure captions with XP spectra.
• Supplementary Fig. 25: There is an error in the labeling of panels (a-f).

Reviewer #3

(Remarks to the Author)
The study describes the therapeutic effects of a novel potassium single-atom catalysts (K-SACs), which exerts potent catalytic activity with minimal metal toxicity, on sepsis-induced acute lung injury. The authors have adopted both in vitro and in vivo models and offered a comprehensive analysis of potential mechanism of pyroptosis in macrophages also using RNA seq-analysis. The chemical structure and activity of the enzyme appears well characterized with state-of-art technologies. The in vitro data appear robust, well-conceived and supportive of the conclusions. However, there are several concerns about the in vivo models of ALI.
Major comments:
Methods: Cecal ligation and puncture model. Details of the animal model of cecal ligation and puncture are missing (size of needle, number of punctures, use of anesthesia, time of treatment, and time of sacrifice and organ harvesting). The lack of this information makes difficult the interpretation of the severity and reliability of the model. Also, it is not clear why the authors used only male mice. A rationale for exclusion of female mice should be provided
Methods and title mismatch: In both in vitro and in vivo experiments, the authors adopted a pretreatment with the K-SACs. The authors should provide a rationale for this pre-treatment and comment on the pharmacokinetics of the catalyst. Also, the authors should test a post-treatment in ALI models to really demonstrate that catalyst initiate immunotherapy, as the authors claim in the title.
Methods: Lung injury score. Lung injury score is based on a scale of severity but the histological criteria (i.e, hemorrhage, cell infiltration, mucin formation, alveolar patency and others) are not described. Although the author state that "The degree of lung injury was estimated based on the previously reported semiquantitative scoring standard", no reference is provided.
Results: Figure 3. While architecture of lungs appears improved in the groups of animals pre-treated with K-SACs, it appears that there is still a discrete neutrophil infiltration. The authors authors should quantified cell infiltration/neutrophils in the lung.

Version 1:

Reviewer comments:

Reviewer #1

(Remarks to the Author)
The authors have addressed all the points raised by me and the other reviewers in a very detailed and comprehensive manner. In particular, the inclusion of new experiments has helped to clarify several mechanisms involved in the therapeutic effect of the nanosystem (K-SAC), thereby strengthening the manuscript.

From my perspective, the manuscript has been satisfactorily revised, and I consider it suitable for publication in its current form.

Reviewer #2

(Remarks to the Author)
The authors of the manuscript by Lu et al. have provided clear and appropriate responses to the comments raised. The corresponding revisions have been implemented consistently and have improved the overall clarity and presentation of the manuscript. The main issues have been adequately addressed, and no further major concerns remain. In its current form, the manuscript can be considered suitable for publication.

Reviewer #3

(Remarks to the Author)

Thank you for your careful and thoughtful responses to the review comments. The revisions effectively address the prior concerns. I have no further comments.

Open Access This Peer Review File is licensed under a Creative Commons Attribution 4.0 International License, which permits use, sharing, adaptation, distribution and reproduction in any medium or format, as long as you give appropriate credit to the original author(s) and the source, provide a link to the Creative Commons license, and indicate if changes were made.
In cases where reviewers are anonymous, credit should be given to 'Anonymous Referee' and the source.
The images or other third party material in this Peer Review File are included in the article's Creative Commons license, unless indicated otherwise in a credit line to the material. If material is not included in the article's Creative Commons license and your intended use is not permitted by statutory regulation or exceeds the permitted use, you will need to obtain permission directly from the copyright holder.
To view a copy of this license, visit https://creativecommons.org/licenses/by/4.0/

Response to Reviewer #1.

Comments from Reviewer #1:

The manuscript by Xiangyu Lu and colleagues presents a novel therapeutic approach for acute lung injury (ALI) using potassium-based single-atom catalysts (K-SACs). The authors demonstrate that K-SACs exhibit dual SOD- and CAT-like enzymatic activities, enabling ROS scavenging and inhibition of pyroptosis. The work is well-executed with comprehensive characterization of the nanomaterial and mechanistic investigations both in vitro and in vivo. The concept of using s-block metal single-atom catalysts represents an innovative approach to address metal toxicity concerns associated with transition metal-based therapeutics.

The use of potassium as the catalytic center is innovative and addresses biocompatibility concerns inherent to transition metal catalysts. The structural and catalytic properties of K-SACs are thoroughly characterized using relevant techniques (DLS, HAADF-STEM, EXAFS, DFT calculations, etc.). The study provides detailed mechanistic insights into pyroptosis inhibition, including ROS scavenging, GSDMD downregulation, ESCRT-mediated membrane repair, and phospholipid remodeling. The validation in both LPS-induced and CLP-induced ALI models strengthens the therapeutic relevance. The manuscript is generally well-written with logical flow and appropriate use of figures.

However, some flaws need to be addressed to improve the quality of the manuscript.

Response: Thank you very much for the positive comment and kind recommendation. Please find the following detailed responses to your suggestions.

Major Concerns

1. The immunofluorescence images in Figure 6a showing F4/80 and GSDMD co-localization are not convincing. The GSDMD signal appears to follow an organized tissue pattern more consistent with epithelial cells (bronchial and alveolar) rather than infiltrating macrophages, as GSDMD is abundantly expressed in airway epithelium (as seen in Figure 1B from 10.1038/s41419-023-06258-1). The yellow co-localization is visible in only a few cells, raising concerns about the conclusion that K-SACs primarily

target macrophage pyroptosis. Co-staining with epithelial markers (e.g., E-cadherin, EpCAM) is essential to confirm cell-type-specific GSDMD expression. Additionally, in vitro co-culture experiments with macrophages and epithelial cells would clarify which cell type is primarily affected. The conclusions should be revised to acknowledge potential epithelial cell effects. While the authors demonstrate high macrophage accumulation in vitro, the mechanism driving macrophage selectivity is not explored.

Response: We appreciate the reviewer's comments, which are very helpful for improving our study. We appreciate this insightful comment that the original Figure 6a did not sufficiently distinguish between macrophage and epithelial GSDMD expression, and that our previous claims regarding "macrophage-specific targeting" were overstated. To address this concern, we have performed multiplex fluorescence staining on lung tissues from mice treated with Cy5.5-labeled K-SAC, targeting lung epithelial cells (EpCAM), endothelial cells (CD31), neutrophils (MPO), and macrophages (F4/80). This represents the most straightforward approach to identify which cell populations are targeted by K-SAC. The staining results showed that Cy5.5-labeled K-SAC colocalized extensively with the macrophage marker F4/80, but not with markers of epithelial or endothelial cells (revised **Supplementary Figure 14**).

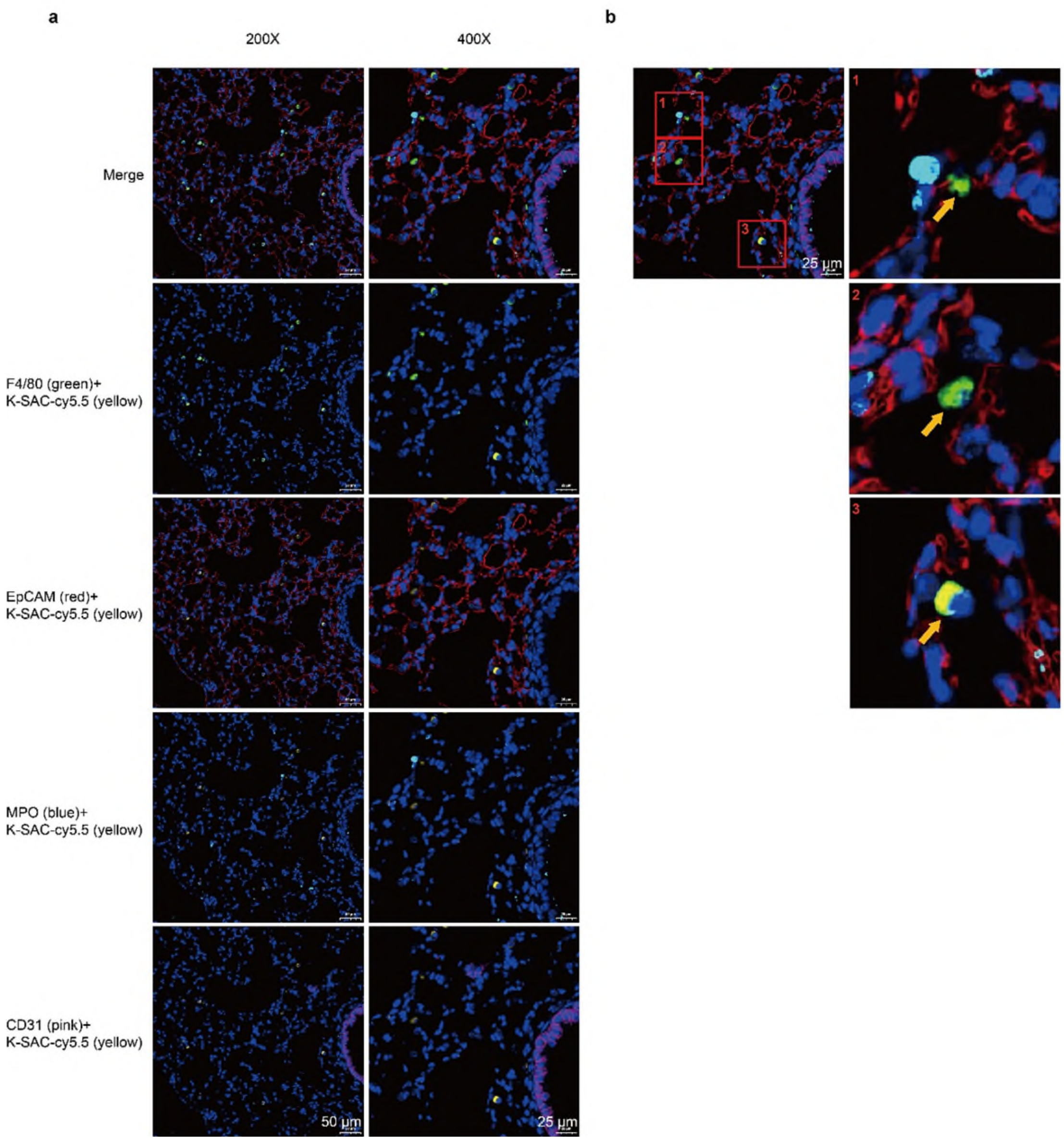

Supplementary Fig. 14. Immunofluorescence co-staining images for different cell types with Cy5.5-labeled K-SAC in mouse lung tissue.

a. Immunofluorescence co-staining images for F4/80 (green), EpCAM (red), MPO (blue) and CD31 (pink) and K-SAC-cy5.5 (yellow) in mouse lung tissue. **b.** The enlarged immunofluorescence.

As various cell types in lung tissue form an integrated organ with extensive crosstalk, the alleviation of lung injury by K-SAC may involve effects on alveolar epithelial cells. It has been reported that oncostatin M (OSM) derived from alveolar macrophages can drive the repair of lung epithelial cells[1], and reduced secretion of pro-inflammatory factors from macrophages can effectively alleviate epithelial cell injury[2]. Therefore, epithelial cells also play an important role in the process by which K-SAC mitigate lung

injury. However, since we observed colocalization of K-SAC-Cy5.5 with macrophage markers in lung tissues after K-SAC treatment, we mainly investigated the therapeutic effects of K-SAC in macrophages. Future studies will further explore how K-SAC regulate pyroptosis in epithelial cells through macrophages.

In revised manuscript, we clarified that K-SAC preferentially uptake by macrophages is primarily driven by the physical properties of the K-SAC (hydrodynamic diameter of ~200 nm and mesoporous structure) and the inherent highly phagocytic nature of activated alveolar macrophages. We have added a detailed discussion on this passive targeting mechanism in the **Results section 5** to address the reviewer's concern.

The part of **Results section 5** is revised as follows:

"We systematically evaluated the biomedical potential of K-SAC by examining their cellular uptake, therapeutic efficacy, and safety profile using both in vitro and in vivo models. Following nasal administration of Cy5.5-labeled K-SAC, strong fluorescence signals are detected in lung tissues (Fig. 3a). Immunofluorescence staining demonstrates co-localization of the macrophage marker F4/80 with Cy5.5 signals (Fig. 3b; Supplementary Figure 14), confirming efficient macrophage uptake. This finding is further supported by in vitro studies with bone marrow-derived macrophages (BMDMs) and peritoneal macrophages (PMs). After 24-hour incubation with Cy5.5-labeled K-SAC in transwell systems, fluorescent particles (K-SAC) are detected in the lower compartment, and TEM images of BMDMs (Fig. 3c,d) and PMs (Supplementary Fig. 15,16) visualize intracellular accumulation of K-SAC, confirming preferential macrophage uptake. This preferential uptake is primarily driven by a passive targeting mechanism. The specific physical properties of K-SAC, including their hydrodynamic diameter of ~200 nm and abundant mesoporous structure, synergize with the inherently high phagocytic capacity of activated alveolar macrophages in the inflammatory microenvironment to facilitate this efficient internalization."

1. Hoagland DA, *et al.* Macrophage-derived oncostatin M repairs the lung epithelial barrier during inflammatory damage. *Science (New York, NY)* **389**, 169-175 (2025).

2. Ruscitti C, *et al.* Recruited atypical Ly6G(+) macrophages license alveolar regeneration after

lung injury. *Science immunology* **9**, eado1227 (2024).

2. K-SACs may affect multiple cell types beyond macrophages (neutrophils, epithelial cells, endothelial cells). The manuscript focuses exclusively on macrophages without exploring broader cellular effects. Assessing K-SACs uptake and effects in other relevant lung cell types, particularly neutrophils given their role in ALI pathogenesis and epithelial cells, would strengthen the work and clarify the role of K-SAC in ALI.

Response: We appreciate the reviewer's comments, which are very helpful for improving our study. As noted in your comments, neutrophils and epithelial cells play critical roles in acute lung injury (ALI)[1, 2]. As shown in **Supplementary Figure 14**, K-SAC-Cy5.5 signals colocalized extensively with the macrophage marker F4/80 in lung tissues, confirming that K-SAC mainly target alveolar macrophages. However, the number of neutrophils colocalized with K-SAC-Cy5.5 is far less than macrophages. Thus, we focused our study on the role of macrophages in K-SAC-mediated ALI therapy.

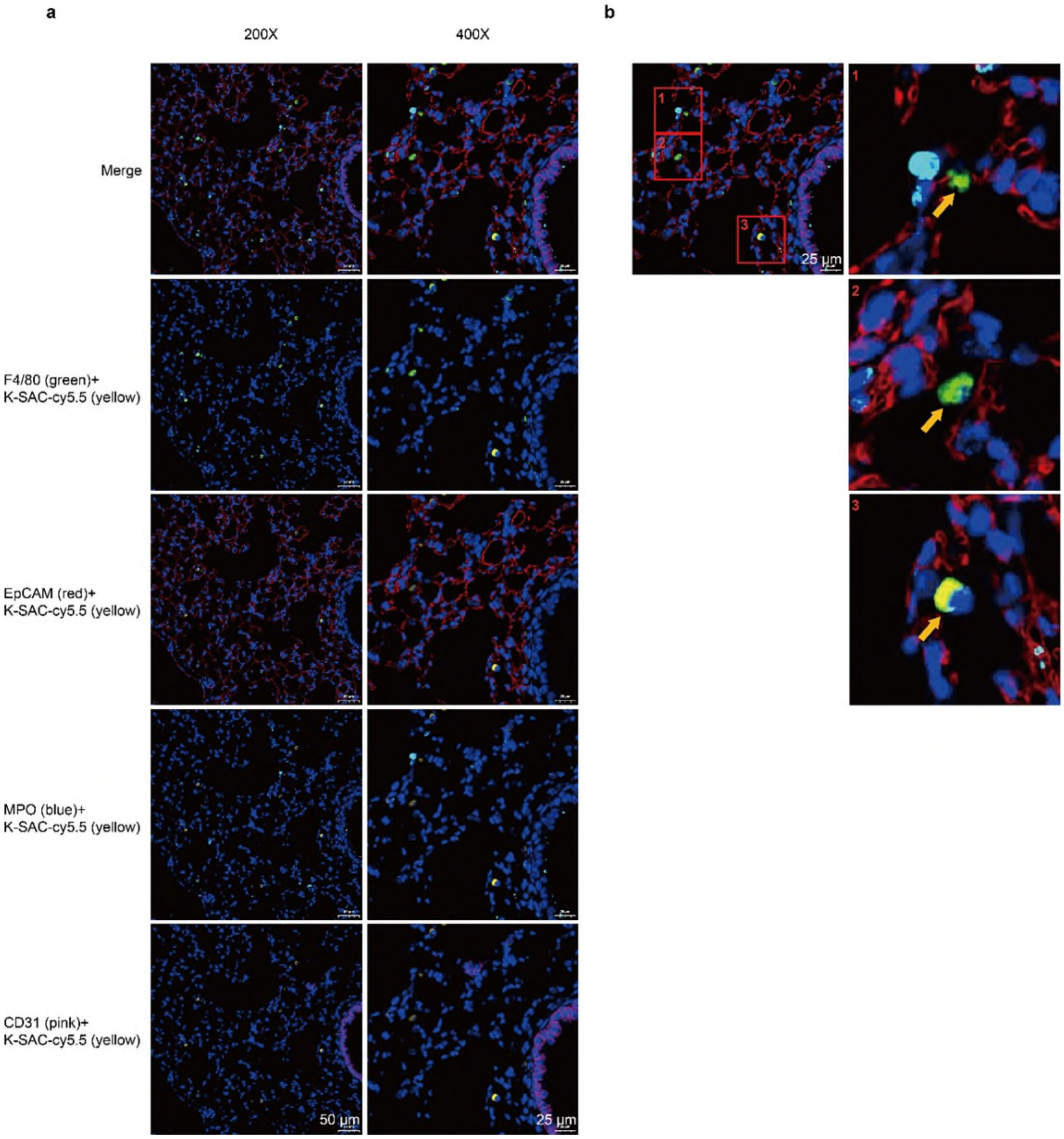

Supplementary Fig. 14. Immunofluorescence co-staining images for different cell types with Cy5.5-labeled K-SAC in mouse lung tissue.

a. Immunofluorescence co-staining images for F4/80 (green), EpCAM (red), MPO (blue) and CD31 (pink) and K-SAC-cy5.5 (yellow) in mouse lung tissue. **b.** The enlarged immunofluorescence.

Per your suggestion, we depleted alveolar macrophages in mice using clodronate liposomes. After macrophage depletion, the therapeutic effect of inhaled K-SAC was markedly attenuated (**Supplementary Figure 21 a,b**), verifying the indispensable role of macrophages in K-SAC treatment for ALI.

Additionally, we detected the neutrophil marker MPO in lung tissues of control, LPS, LPS+K-SAC, and LPS+NAC groups. K-SAC intervention reduced pulmonary MPO

expressions (**Supplementary Figure 21 c,d**), **indicating that K-SAC modulates neutrophils during ALI treatment**. During inflammation, macrophages secrete large amounts of chemokines, which recruit neutrophils to accumulate at the injured site. After inflammation is resolved, the secretion of chemokines decreases, leading to a marked reduction in neutrophil chemotaxis and the observed decrease in MPO.

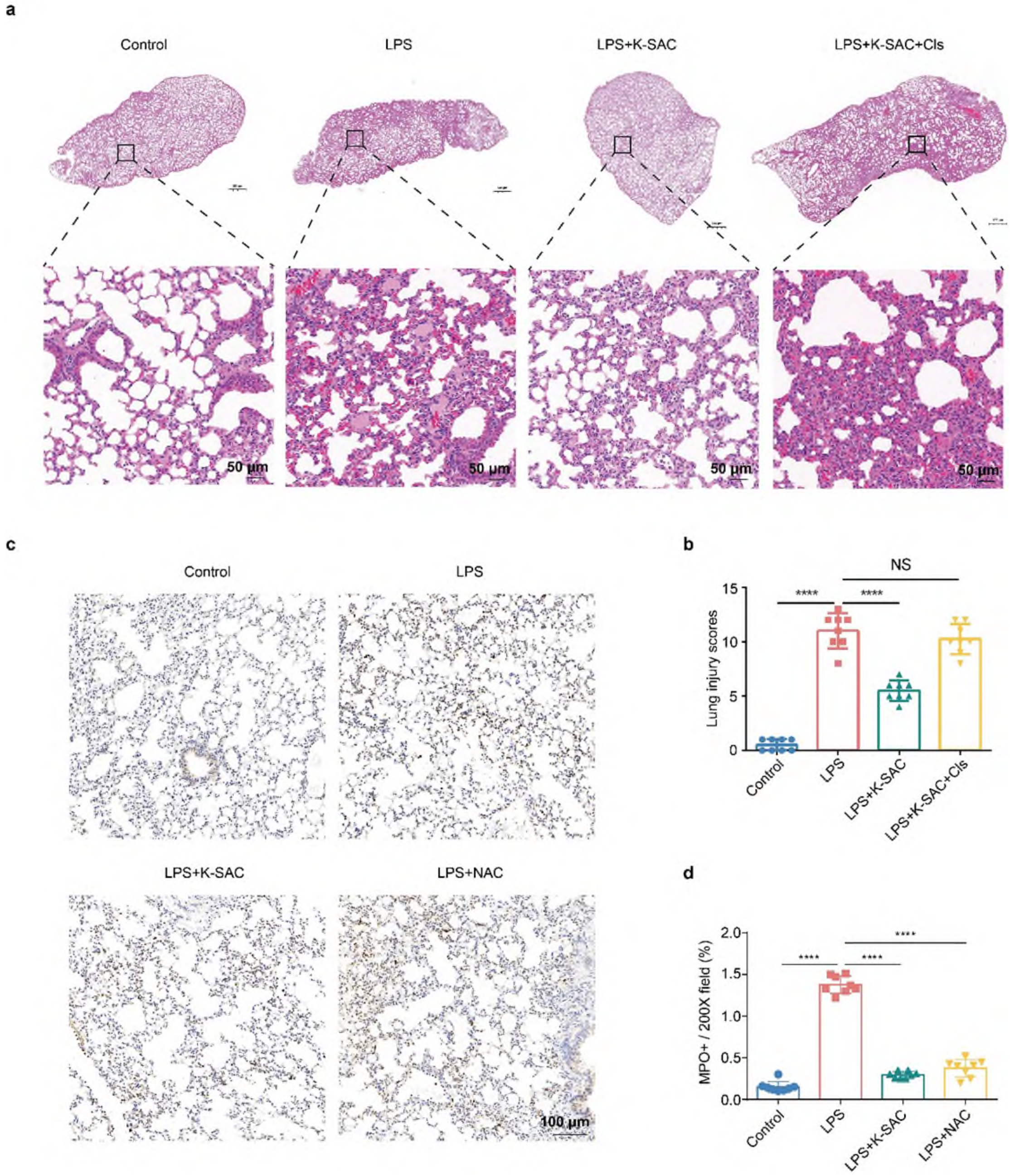

Supplementary Fig. 21. Macrophages play a critical role in the alleviation of lung injury by K-SAC.

a,b, The therapeutic effect of K-SAC was significantly attenuated after intraperitoneal macrophage

depletion. **c,d,** Immunohistochemistry detection of neutrophil myeloperoxidase (MPO) in lung tissue.

The part of **Results section 5** is revised as follows:

"The therapeutic potential of K-SAC was assessed in two established ALI models induced by lipopolysaccharide (LPS) and cecal ligation and puncture (CLP), respectively. Histological examination shows that K-SAC treatment substantially attenuated pathological changes, including alveolar wall thickening and inflammatory cell infiltration (Fig. 3e,f). Quantitative analysis confirms significant improvement in lung injury scores (Fig. 3g,h), accompanied by the reduced IL-1β level (Fig. 3i,j), indicating potent anti-inflammatory activity, which is comparable to N-acetylcysteine (NAC) treatment. To investigate the role of macrophages in the therapeutic effect of K-SAC against ALI, we depleted macrophages in lung tissue using clodronate liposomes and examined whether the therapeutic efficacy of K-SAC was altered. We found that the therapeutic effect of K-SAC on lung injury was markedly attenuated following macrophage depletion (Supplementary Fig. 21a,b), demonstrating that macrophages are essential for K-SAC to ameliorate acute lung injury. Furthermore, our experiments revealed a marked decrease in pulmonary neutrophil accumulation following K-SAC treatment (Supplementary Fig. 21c,d). Because K-SAC exclusively co-localized with macrophages in vivo, we directed our current mechanistic focus toward macrophage modulation. The precise direct or indirect role of neutrophils in this therapeutic process will be explored in future studies."

The part of **Outlook section** is revised as follows:

"In this study, we have developed a novel kind of potassium-based single-atom catalysts (K-SAC) by making use of potassium (K), the most abundant intracellular metal in human bodies, and demonstrated that K-SAC can (1) effectively scavenge ROS and suppress ROS-induced inflammatory responses by mimicking the catalytic activities of SOD and CAT; (2) inhibit GSDMD-mediated pyroptosis, and meanwhile activate the ESCRT system and membrane phospholipid remodeling to mitigate macrophage pyroptosis; (3) effectively protect mitochondrial integrity; (4) effectively attenuate macrophage infiltration, inflammatory cascades, and oxidative stress in ALI with

excellent biocompatibility. Notably, K-SAC alleviates both LPS- and sepsis-induced ALIs, two typical conditions characterized by immune dysregulation.

Furthermore, the inherent heterogeneity of macrophage populations presents a critical avenue for future precision medicine. Because monocyte-derived macrophages (MoMs) function as the primary drivers of early-stage, pyroptosis-prone inflammation, future material optimization should focus on functionalizing K-SAC to selectively target these specific infiltrating subsets. Tailoring nanomaterial targeting capabilities based on the dynamic spatiotemporal distribution of these subsets will pave the way for more precise immunotherapeutic interventions. Beyond macrophages, navigating the broader pulmonary immune network, particularly the involvement of neutrophils, will be a key focus of our future investigations. Neutrophils play a dual role in lung injury, functioning in pathogen surveillance while also driving pulmonary microcirculatory dysfunction via capillary occlusion. While our experimental data demonstrated a marked decrease in pulmonary neutrophil accumulation following K-SAC administration, the strict in vivo co-localization of inhaled K-SAC exclusively with macrophages led us to center our current mechanistic exploration on macrophage modulation."

1. Hsu AY, *et al.* Neutrophil-derived vesicles control complement activation to facilitate inflammation resolution. *Cell* **188**, 1623-1641.e1626 (2025).
2. Chung KP, *et al.* Alveolar epithelial cells mitigate neutrophilic inflammation in lung injury through regulating mitochondrial fatty acid oxidation. *Nat Commun* **15**, 7241 (2024).

3. While the authors demonstrate that K-SACs reduce GSDMD expression and pyroptotic markers, they do not directly prove that pyroptosis inhibition is GSDMD-dependent. The stated conclusion that K-SACs work "by inhibiting macrophage pyroptosis" would be strengthened by loss-of-function experiments. Including experiments with GSDMD- or ESCRT-deficient macrophages (knockout or siRNA knockdown) treated with K-SACs would be valuable. If pyroptosis inhibition persists in GSDMD- or ESCRT-null cells, alternative mechanisms should be investigated. This

would definitively establish whether GSDMD is the primary target.

Response: We appreciate the reviewer's insightful suggestions. To establish the direct causal relationship between the therapeutic effects of K-SACs with GSDMD, loss-of-function experiments are indispensable.

We knocked down GSDMD in primary macrophages. Classical pyroptosis was induced in vitro by stimulation with LPS (1 μg/ml) for 5 hours, followed by ATP (5 mM) for 1 hour. The experiment was divided into five groups: control, LPS+ATP, LPS+ATP+K-SAC, si-GSDMD+LPS+ATP, and si-GSDMD+LPS+ATP+K-SAC groups. The levels of IL-1β and IL-18 in the culture supernatant were determined by ELISA to verify that the inhibitory effect of K-SAC on pyroptosis is GSDMD-dependent. The results showed that K-SAC significantly reduced the concentration of IL-1β and IL-18. However, in si-GSDMD cells, treatment with K-SAC did not confer any further protective effect compared with untreated si-GSDMD cells.

This absence of an additive protective effect suggests that the anti-pyroptotic and anti-inflammatory effects of K-SAC depend on inhibition of the GSDMD pathway. The relevant data have been added to **Figure 4m,n**, **supplementary Figure 31** and the **Supplementary Materials** in the revised manuscript.

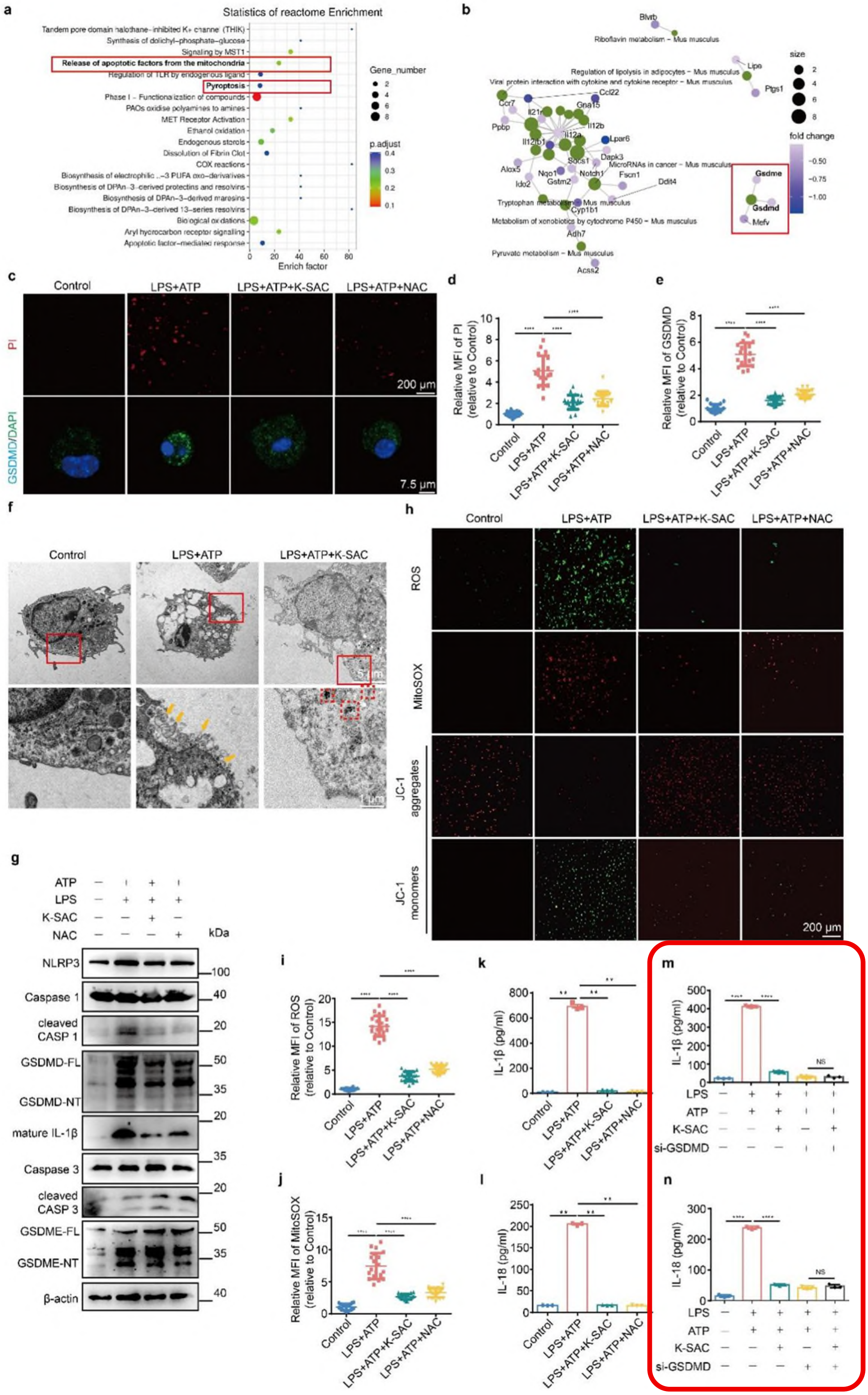
a
Statistics of reactome Enrichment
Tandem pore domain halothane-inhibited K+ channel (THIK)
Synthesis of dolichyl-phosphate-glucose
Signaling by MST1
Release of apoptotic factors from the mitochondria
Regulation of TLR by endogenous ligand
Pyroptosis
Phase I - Functionalization of compounds
PAOs oxidise polyamines to amines
MET Receptor Activation
Ethanol oxidation
Endogenous sterols
Dissolution of Fibrin Clot
COX reactions
Biosynthesis of DPAn-3-derived protectins and resolvins
Biosynthesis of DPAn-3-derived maresins
Biosynthesis of DPAn-3-derived 13-series resolvins
Biological oxidations
Aryl hydrocarbon receptor signalling
Apoptotic factor-mediated response
Enrich factor
Gene_number
p.adjust
b
Blvrb
Riboflavin metabolism - Mus musculus
Regulation of lipolysis in adipocytes - Mus musculus
Lipe
Ptgs1
Viral protein interaction with cytokine and cytokine receptor - Mus musculus
Ccl22
Ccr7
Il21r
Gna15
Il12b
Pppb
Il12rb1
Il12a
Lpar6
Socs1
Dapk3
MicroRNAs in cancer - Mus musculus
Alox5
Nqo1
Notch1
Fscn1
Ido2
Gstm2
Ddit4
Tryptophan metabolism
Cyp1b1
Metabolism of xenobiotics by cytochrome P450 - Mus musculus
Adh7
Pyruvate metabolism - Mus musculus
Acss2
Gsdme
Gsdmd
Mefv
size
fold change
c
Control
LPS+ATP
LPS+ATP+K-SAC
LPS+ATP+NAC
PI
GSDMD/DAPI
200 μm
7.5 μm
d
Relative MFI of PI (relative to Control)
e
Relative MFI of GSDMD (relative to Control)
f
Control
LPS+ATP
LPS+ATP+K-SAC
5 μm
1 μm
h
Control
LPS+ATP
LPS+ATP+K-SAC
LPS+ATP+NAC
ROS
MitoSOX
JC-1 aggregates
JC-1 monomers
200 μm
g
ATP
LPS
K-SAC
NAC
kDa
NLRP3
Caspase 1
cleaved CASP 1
GSDMD-FL
GSDMD-NT
mature IL-1β
Caspase 3
cleaved CASP 3
GSDME-FL
GSDME-NT
β-actin
i
Relative MFI of ROS (relative to Control)
j
Relative MFI of MitoSOX (relative to Control)
k
IL-1β (pg/ml)
l
IL-18 (pg/ml)
m
IL-1β (pg/ml)
NS
LPS
ATP
K-SAC
si-GSDMD
n
IL-18 (pg/ml)
NS
LPS
ATP
K-SAC
si-GSDMD

Fig. 4: K-SAC alleviate GSDMD-mediated pyroptosis and mitochondrial damage in macrophages.

a,b, Reactome **(a)** and KEGG **(b)** enrichment analyses revealing the significantly inhibition of pyroptosis-related pathways by K-SAC in LPS+ATP-stimulated BMDMs. **c-e,** Representative PI immunofluorescent staining **(c, upper panel)** and GSDMD immunofluorescent staining **(c, lower panel)** images in LPS and ATP-stimulated BMDMs with K-SAC or NAC treatment (Scale bar: 200 μm, upper panel; 7.5 μm, lower panel). The MFI quantifications of PI **(d)** and GSDMD **(e)**. **f,** TEM images of BMDMs: yellow arrows indicate damaged membranes and pyroptotic bodies after LPS and ATP stimulation (Scale bar: upper, 5 μm; lower, 1 μm). **g,** Western blot analyses of NLRP3, Caspase-1, cleaved Caspase-1, GSDMD, mature IL-1β, Caspase-3, cleaved Caspase-3, GSDME, and β-actin in BMDMs. **h,** Fluorescence images of ROS, mitoSOX, and JC-1 staining in BMDMs. **i,j,** MFI values of ROS **(i)** and mitoSOX **(j)**. **k,l,** IL-1β **(k)** and IL-18 **(l)** levels in BMDMs supernatants. **m,n,** IL-1β **(m)** and IL-18 **(n)** levels in BMDMs supernatants treated with K-SAC, LPS and ATP, with or without GSDMD siRNA intervention. The results are presented as mean ± SD with $n = 3$ **(k-n)** independent samples for each group. Statistical significance was calculated via one-way ANOVA with a Tukey post-hoc test **(d-e, i-n)**.

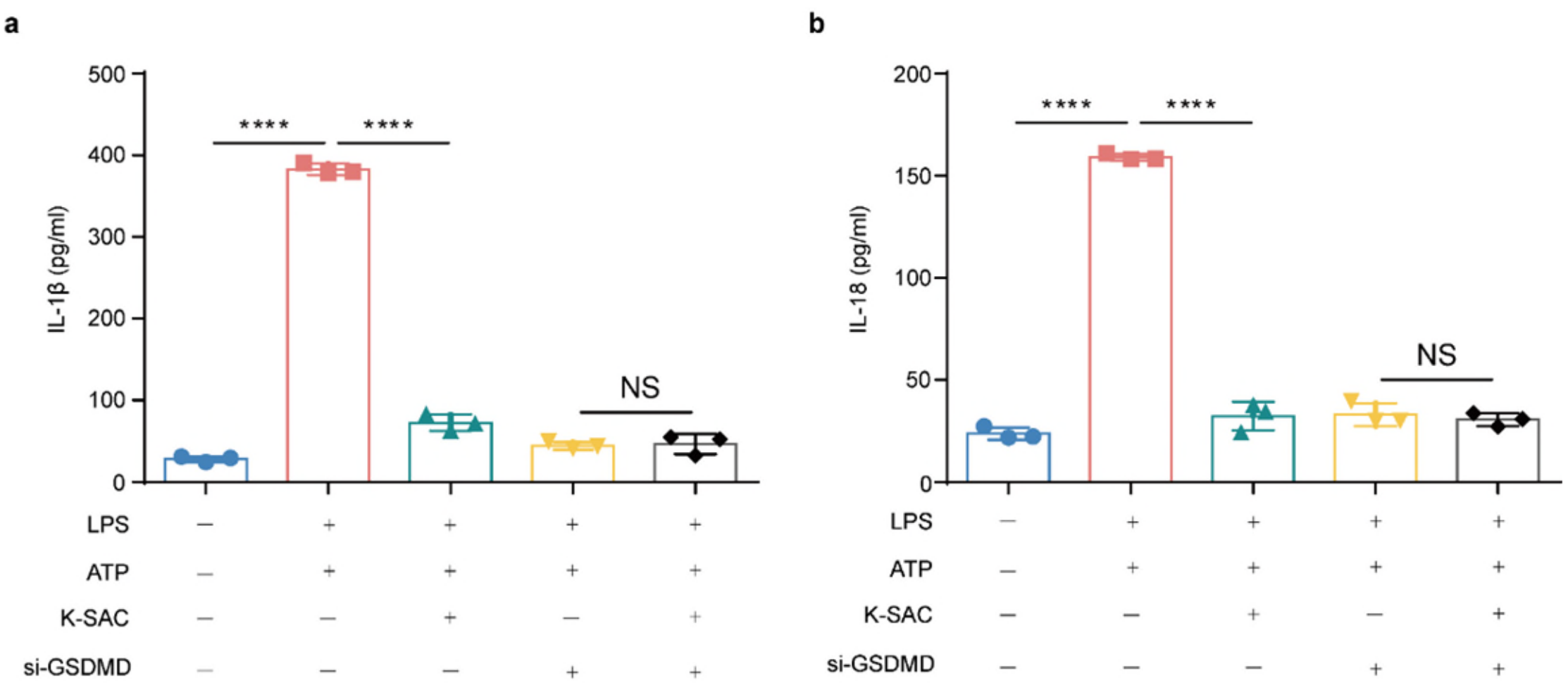

Supplementary Fig. 31. Anti-inflammation effects of K-SAC is GSDMD-dependent.

IL-1β **(a)** and IL-18 **(b)** levels in PMs supernatants treated with K-SAC, LPS and ATP, with or without GSDMD siRNA intervention.

The part of **Supplementary Methods 13** is revised as follows:

"13. RNA interference

Small interfering RNAs (siRNAs) targeting GSDMD were obtained from GenePharma.

Sequence was shown in Supplementary Table 5. Transfection was performed using the INTERFERin reagent (101000028; Polyplus) according to the manufacturer's protocol. Knockdown efficiency was confirmed by RT-qPCR (data not shown)."

We detected the anti-inflammatory effect of K-SAC in primary macrophages with GSDMD knockdown to establish the primary anti-pyroptotic mechanism. Meanwhile, we respectfully offer the following rationale for why ESCRT knockdown experiments were not performed:

Studies have confirmed that the ESCRT machinery modulates multiple cell death pathways, with its core function being the repair of damaged plasma and intracellular membranes. Deficiency in the ESCRT system impairs the cellular capacity to resolve membrane damage, allowing membrane disruption triggered by various death signals to proceed unchecked, thereby rendering cells more susceptible to death[1]. Interference with key genes encoding ESCRT components results in prominent cellular injury and plasma membrane rupture. For these reasons, we did not perform knockdown or interference experiments targeting ESCRT components. In addition, Rühl et al. demonstrated in *Science* that the ESCRT machinery acts downstream of GSDMD[2]. Accordingly, GSDMD knockdown is sufficient to validate the mechanism underlying the effects of K-SAC.

1. Liu J, Kang R, Tang D. ESCRT-III-mediated membrane repair in cell death and tumor resistance. Cancer Gene Ther. 2021 Feb;28(1-2):1-4.
2. Rühl S, Shkarina K, Demarco B, Heilig R, Santos JC, Broz P. ESCRT-dependent membrane repair negatively regulates pyroptosis downstream of GSDMD activation. Science. 2018 Nov 23;362(6417):956-960.

4. The methods describe three K-SACs administration regimens (48h, 24h, and simultaneous with LPS), but the results section does not clarify which protocol was used for Figure 6, nor are comparative data presented. Understanding the therapeutic window is critical for clinical translation.

Response: Thank you for your comments. We apologize for the inaccurate description

of the administration timing in the original manuscript, which may have caused confusion. Actually, mice received a total of three inhaled administrations, at 48 h before model establishment, 24 h before model establishment, and concurrently with model establishment. We have revised the description in the **Methods section 8** of the revised manuscript as follows:

"For LPS-induced acute lung injury, 10 mg/kg LPS was injected intraperitoneally. Before SI-ALI, mice were administered K-SAC (1 mg/kg in PBS) at 48 h before, 24 h before, and concurrently with LPS challenge."

Minor Concerns

5. BMDMs and PMs exhibit different phenotypic profiles and responses to stimulation, as evident in Supplementary Figure 21 showing differential GSDMD expression. These cell-type-specific differences are not addressed in the discussion. Including a brief discussion on how macrophage heterogeneity might influence K-SACs efficacy and whether targeting specific macrophage subsets would be advantageous could strengthen the manuscript.

Response: Thank you for your attention to the issue of macrophage heterogeneity. BMDMs and PMs display substantial differences in developmental origin, surface markers, and functions: BMDMs are more skewed toward an inflammatory phenotype and play a prominent role in pathogen clearance and pro-inflammatory responses, whereas PMs exhibit stronger phagocytic activity and greater tissue repair potential[1]. In lung tissue, alveolar macrophages are moderately sensitive to pyroptosis, whereas monocyte-derived macrophages (major effector cells in acute lung injury) are highly sensitive and readily activated by LPS or DAMPs. Since BMDMs and lung-infiltrating macrophages are both monocyte-derived, they respond similarly to stimulation and treatment. As a type of primary macrophage, PMs also reflect in vivo macrophage behavior, which was reported in *Nature Communications*[2]. We therefore used both cell types for more solid outcomes.

As the reviewer observed, K-SAC showed minor differences in regulating GSDMD mRNA between BMDMs and PMs. However, western blot (Figure 4 g; Supplementary

Figure 27) and immunofluorescence (Figure 4 c,e; Supplementary Figure 25) verified that K-SAC effectively decreased GSDMD protein expression in both cells.

We have revised the description in the **Outlook section** of the revised manuscript as follows (The underlined parts address the reviewer's question):

Outlook

In this study, we have developed a novel kind of potassium-based single-atom catalyst (K-SAC) by making use of potassium (K), the most abundant intracellular metal in human bodies, and demonstrated that K-SAC can (1) effectively scavenge ROS and suppress ROS-induced inflammatory responses by mimicking the catalytic activities of SOD and CAT; (2) inhibit GSDMD-mediated pyroptosis, and meanwhile activate the ESCRT system and membrane phospholipid remodeling to mitigate macrophage pyroptosis; (3) effectively protect mitochondrial integrity; (4) effectively attenuate macrophage infiltration, inflammatory cascades, and oxidative stress in ALI with excellent biocompatibility. Notably, K-SAC alleviate both LPS- and sepsis-induced ALIs, two typical conditions characterized by immune dysregulation.

<u>Furthermore, the inherent heterogeneity of macrophage populations presents a critical avenue for future precision medicine. Because monocyte-derived macrophages (MoMs) function as the primary drivers of early-stage, pyroptosis-prone inflammation, future material optimization should focus on functionalizing K-SAC to selectively target these specific infiltrating subsets. Tailoring nanomaterial targeting capabilities based on the dynamic spatiotemporal distribution of these subsets will pave the way for more precise immunotherapeutic interventions.</u> Beyond macrophages, navigating the broader pulmonary immune network, particularly the involvement of neutrophils, will be a key focus of our future investigations. Neutrophils play a dual role in lung injury, functioning in pathogen surveillance while also driving pulmonary microcirculatory dysfunction via capillary occlusion. While our experimental data demonstrated a marked decrease in pulmonary neutrophil accumulation following K-SAC administration, the strict in vivo co-localization of inhaled K-SAC exclusively with macrophages led us to center our current mechanistic exploration on macrophage

modulation.

1. Bisgaard LS, *et al.* Bone marrow-derived and peritoneal macrophages have different inflammatory response to oxLDL and M1/M2 marker expression - implications for atherosclerosis research. *Scientific reports* **6**, 35234 (2016).
2. Wei Y, *et al.* GSDME-mediated pyroptosis promotes the progression and associated inflammation of atherosclerosis. *Nat Commun* **14**, 929 (2023).

6. Several figure legends lack essential information. Some examples:

o Figure 3e,f: NAC concentration not specified

o Figure 4: Drug concentrations and incubation times inconsistently reported

o Figure S25: Figure lettering is incorrect from b-e; f is missing

o The abbreviation OPLS-DA in Supplementary Fig. 30 is not described in the text (not even in the methodological section)

Systematically review all figure legends to include drug concentrations (such as for CeO_2 and Mn_3O_4), incubation times, and sample sizes. Ensure methods are fully detailed. Figures 5f-g present phospholipid composition changes but do not clearly state whether the percentages represent treated (LPS+ATP+K-SAC) vs. control or just treated samples. Presenting side-by-side comparisons showing lipid composition in control, LPS+ATP, and LPS+ATP+K-SAC groups or clarifying the data would help to understand the experiment. Include statistical analysis of compositional changes.

Response: Thank you for your suggestions.

1) Figure 3e,f: We have clearly indicated the concentration of NAC for in vitro intervention as 5 mM in all relevant Methods sections. For inhaled NAC, the administration protocol is as follows: inhalation was performed once at three time points, at a concentration of 3.6 mg/ml and a volume of 2 ml per treatment[1]. We have added the detailed concentration and inhalation timing of NAC in **Methods section 8**.

1. Sun D, et al. Softness enhanced macrophage-mediated therapy of inhaled apoptotic-cell-inspired nanosystems for acute lung injury. Journal of nanobiotechnology 21, 172 (2023).

The reference (*Nat Protoc* **4**, 31-36 (2009)) has been cited as ref. [55].

The revised **Methods section 8** is shown below:

"**8. ALI Mouse Models and Treatment**

For LPS-induced acute lung injury, 10 mg/kg LPS was injected intraperitoneally. Mice were administered K-SAC (1 mg/kg in PBS) or NAC (360 mg/kg in PBS) at 48 h before, 24 h before, and concurrently with LPS challenge. The sepsis-induced ALI model was induced by cecal ligation and puncture (CLP) as standardized previously[55]. Mice were anesthetized with 2,2,2-tribromoethanol (400 mg/kg, i.p.). After a midline abdominal incision, the cecum was exposed and 50% of the distal segment was ligated with 4-0 silk suture without blocking the ileocecal valve. A single through-and-through puncture was made with a 21-gauge needle, and a small volume of feces was gently extruded. The cecum was repositioned and the incision was closed layer-by-layer. Sham-operated mice underwent the same procedure without ligation and puncture. All animals received subcutaneous fluid resuscitation (prewarmed saline, 5 mL/100 g) postoperatively. 24 h after lung injury, mice were sacrificed for further analysis."

55. Rittirsch, D., Huber-Lang, M.S., Flierl, M.A. & Ward, P.A. Immunodesign of experimental sepsis by cecal ligation and puncture. Nat Protoc 4, 31-36 (2009).

2) Figure 4: We have added the intervention time and concentration of the drugs in the relevant sections of the revised manuscript.

3) Figure S25: We have corrected the figure lettering.

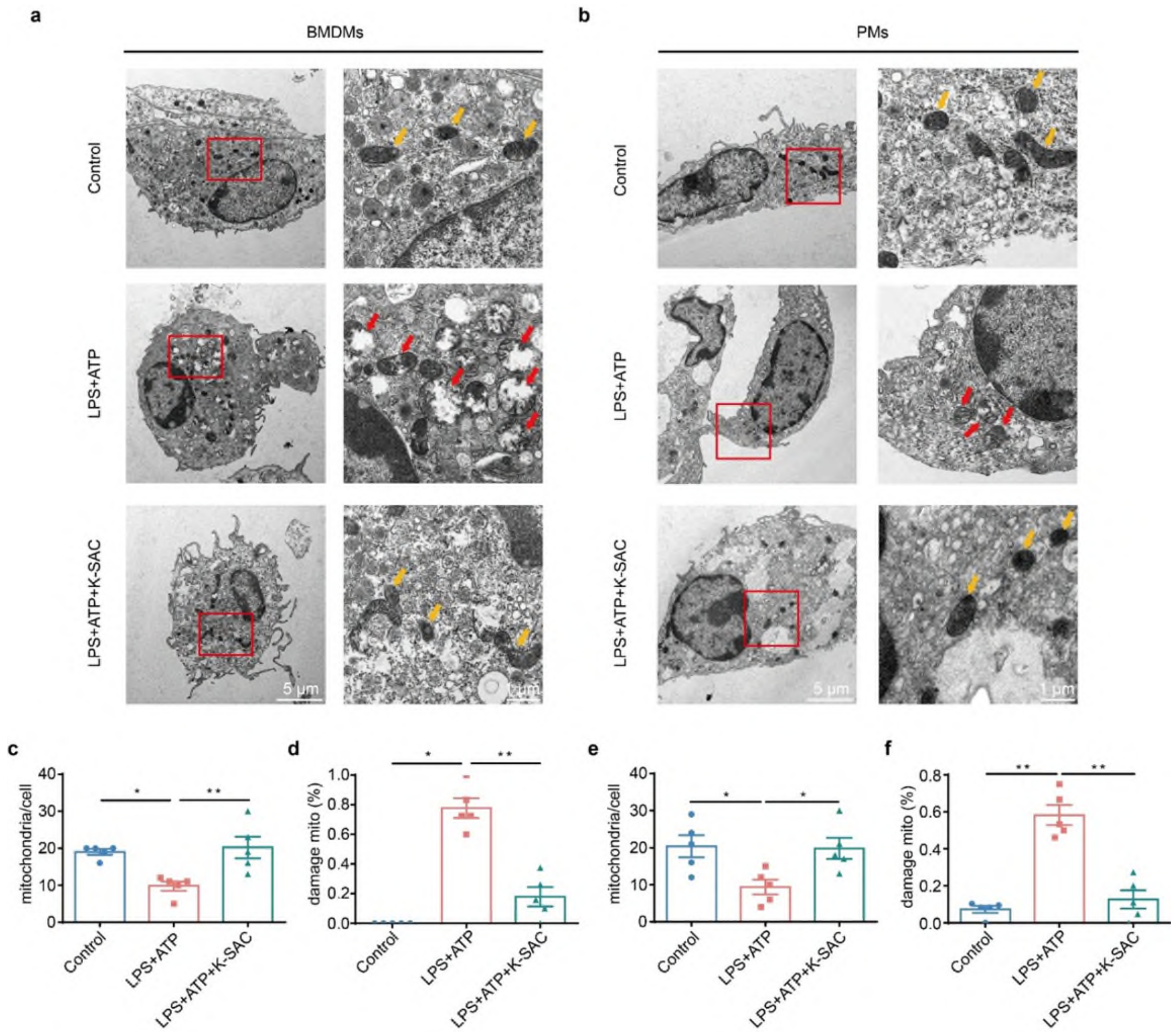

Supplementary Fig. 28. K-SAC alleviate mitochondria damage.

a,b, Representative images of TEM analysis of BMDMs **(a)** and PMs **(b)**. The right panels show higher magnification of the boxed area in the left panels. The yellow arrows pointed the normal mitochondria; red arrows pointed swelling mitochondria (Scale bar, upper: 5 μm, lower: 1 μm). **c-f,** Mitochondrial (mito) number and percentage of damaged mitochondria of BMDMs **(c,d)** and PMs **(e,f)** were quantified.

4) Supplementary Fig. 30: We have added a detailed description of OPLS-DA in **supplementary methods section 7** (Lipid UPLC-QE MS Analysis). The added content is as follows:

"Data were imported into SIMCA-P 11.0 (Umetrics) software for multivariate statistical analysis after normalization. Orthogonal partial least squares discriminant analysis (OPLS-DA) scatter plots were obtained to show the potential biomarkers of lipomics from the obtained data."

5) Figures 5f-g show the classification of differential lipids between the LPS group and

the LPS+K-SAC group. These panels illustrate the classification of the differential lipid metabolites identified in Figure 5e and Supplementary Table 4. With this experiment, we aimed to identify which specific classes of membrane structural phospholipids were remodeled by K-SAC.

Following your suggestion, we have revised the legend of Figure 5 as follows:

f,g, Classification of differential lipids between the LPS group and LPS+K-SAC group as shown in panel e. Lipids were classified by lipid class including PC, PE, SPH, Cer, etc. **(f)**, and also categorized into saturated fatty acids and unsaturated fatty acids based on the presence or absence of unsaturated double bonds **(g)**.

7. Some supplementary figures and tables are not referenced in numerical order (e.g., Fig. S4 before S3). Supplementary Figures 8 and 11 are not cited in the main text.

Response: Thank you for your comments. We have carefully checked the manuscript and ensured that each figure is correctly cited in the appropriate places.

Supplementary Figures 8 have been cited in **Results section 2**:

High-resolution N 1s spectra of the samples were also recorded and deconvoluted into four components: Pyridinic N (398.3 eV), Pyrrolic N (399.1 eV), Graphitic N (401.0 eV) and Oxidized N (402.7 eV) (Supplementary Fig. 8).

Supplementary Figures 11 have been cited in **Results section 3**:

Subsequently, the CAT-like activity of K-SAC was evaluated using a CAT assay kit and UV spectrophotometry. As depicted in Fig. 2d and Supplementary Fig. 10, K-SAC exhibit an enzyme activity of 26.69 U/mg, surpassing both NC and transition metal oxide nanoparticles. Kinetic studies monitoring H_2O_2 depletion at 240 nm reveal time- and concentration-dependent H_2O_2 consumption (Fig. 2e), with degradation efficiency increasing with K-SAC concentration at fixed H_2O_2 levels or with H_2O_2 concentration at fixed K-SAC doses. Lineweaver–Burk plot analysis yields a Michaelis constant (K_m) of 20.379 mM and a maximum reaction velocity (V_m) of 1.843 mM/min (Supplementary Fig. 11), indicating that catalytic activity and substrate affinity are comparable to those of reported transition metal-based single-atom catalysts and nanoparticles.

8. Figure legends describe using one-way ANOVA with Tukey post-hoc test for multiple comparisons, but sample sizes vary (n=3 to n=8). Power analysis is not mentioned. Provide justification for sample sizes and confirm adequate statistical power.

Response: Thank you for your valuable suggestion. We sincerely appreciate the reviewer's insightful comments regarding our sample size calculation and statistical power analysis. We fully agree that the rigorous determination of sample size and explicit validation of statistical power are crucial for ensuring the reliability of our conclusions. For the present study, we determined the sample size strictly based on the research objectives, and the general standards commonly adopted in the field:

1) **In vitro experiments (n=3):**

For cell-based studies, culture conditions (temperature, CO_2 concentration, media composition) were strictly standardized. Cells were derived from the same batch of isolated primary macrophages, minimizing inter-individual variability. In this condition, **n=3 independent biological replicates** are recognized in the field of acute lung injury research.

2) **Animal experiments (n=6-8):**

In vivo studies inherently exhibit higher biological variability due to differences in metabolism, inflammatory responses, and surgical stress among individual mice. To account for this heterogeneity and avoid false-negative results, a sample size of **6-8 mice per group** is recognized in the field of acute lung injury research.

Meanwhile, all key results in this study showed clear statistical significance (*$P<0.05$, **$P<0.01$, etc.) with no marginally significant findings ($P\approx0.05$), confirming that the differences between groups are genuine.

To further improve the description of statistical methods, we will add the following statements in the revised manuscript:

In the Methods section 15 Statistical analysis:

"Statistical analyses were performed using GraphPad Prism 9 software. In vitro experiments used n=3, and in vivo experiments used n=8, consistent with common standards in ALI research. Statistical significance was assessed by unpaired two-tailed

Student's *t* test with a value of $p < 0.05$ considered to be statistically significant (*$p < 0.05$, **$p < 0.01$, ***$p < 0.001$, ****$p < 0.0001$, NS, not significant). All experiments were repeated at least three times, independently. Representative data were obtained from at least three independent experiments. The investigators were blinded to allocation during experiments and outcome assessment."

9. All in vivo experiments use a single dose (1 mg/kg). No dose optimization or dose-response relationship is established. Explain the rationale for the selection of this dose.

Response: Thank you for your comments. The dose of 1 mg/kg was selected based on our preliminary dose-escalation and safety studies, which have now been added to the revised manuscript.

In the safety study, K-SAC were administered alone via aerosol inhalation. No obvious lung injury was observed even at a high dose of 10 mg/kg (**Supplementary Figure 17a,b**), demonstrating the biosafety of K-SAC.

In LPS-induced ALI mice, we further evaluated the therapeutic effects of three inhaled doses: 0.1, 1, and 2.5 mg/kg. We found that at 0.1 mg/kg, K-SAC showed almost no improvement in lung injury, and at 2.5 mg/kg, similar therapeutic efficacy was observed compared with 1 mg/kg (**Supplementary Figure 17c,d**). These results clearly demonstrate that 1 mg/kg is the optimal effective dose.

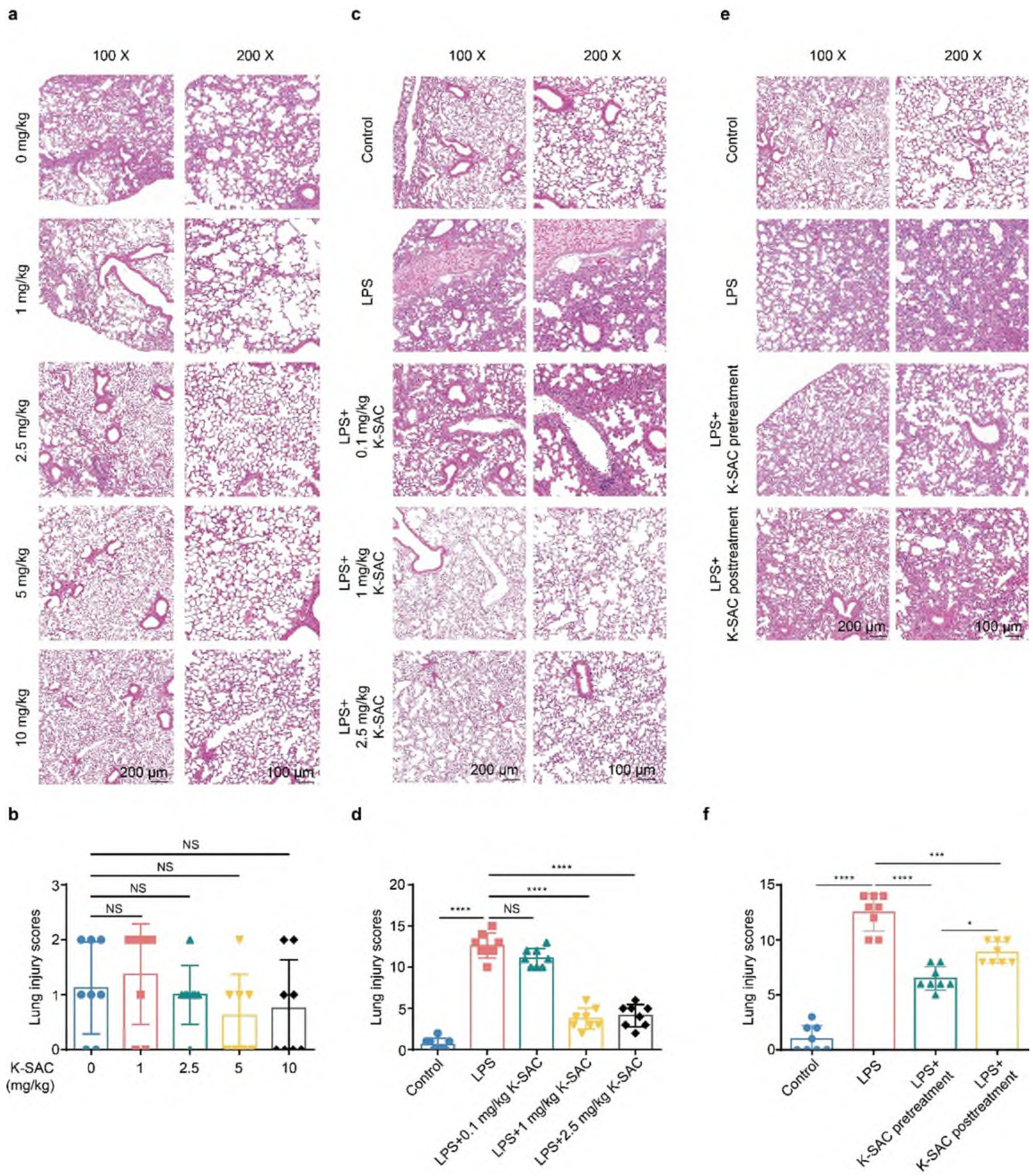

Supplementary Fig. 17. Pulmonary safety evaluation and efficacy detection of different doses of K-SAC.

a,b, Representative H&E staining of mouse lung tissues following nasal inhalation of different doses of K-SAC (1 mg/kg, 2.5 mg/kg, 5 mg/kg, 10 mg/kg) **(a)** and lung injury score **(b)** (scale bar: left, 200 μm; right, 100 μm). **c,d,** Representative H&E staining of mouse lung tissues following LPS injection and nasal inhalation of different doses of K-SAC (0.1 mg/kg, 1 mg/kg, 2.5 mg/kg) **(c)** and lung injury score **(d)** (scale bar: left, 200 μm; right, 100 μm). **e,f,** Representative H&E staining of mouse lung tissues following nasal inhalation of K-SAC (1 mg/kg) before or after LPS injection **(e)** and lung injury score **(f)** (scale bar: left, 200 μm; right, 100 μm).

Accordingly, we used 1 mg/kg as the therapeutic dose in all subsequent in vivo experiments. The corresponding results have been added in the **Results section 5**:

"To evaluate the biosafety and therapeutic potential of K-SAC, mice were intranasally administered K-SAC at doses of 1 mg/kg, 2.5 mg/kg, 5 mg/kg, and 10 mg/kg three times within 48 h. H&E staining revealed no obvious toxicological manifestations following nasal administration (Supplementary Fig. 17a,b). We next examined the therapeutic efficacy of K-SAC at lower doses (0.1, 1, and 2.5 mg/kg), and 1 mg/kg K-SAC exhibited comparable protective effects relative to 2.5 mg/kg (Supplementary Fig. 17c,d). Histopathological examination of major organs and analysis of serum biomarkers (ALT, AST, creatinine, and urea) detected no signs of toxicity following nasal administration (Supplementary Fig. 18,19). Comprehensive safety evaluations reveal the excellent biocompatibility. Cell viability assays showed no significant cytotoxicity at concentrations up to 10 μg/mL (Supplementary Fig. 20). These results collectively demonstrate the favorable safety profile of K-SAC for potential clinical application. Furthermore, we compared the therapeutic outcomes of prophylactic and post-challenge administration of K-SAC, and found that pre-administration conferred superior protection against acute lung injury (Supplementary Fig. 17e,f)."

10. Biocompatibility is assessed only acutely. Chronic effects, tissue accumulation, and long-term outcomes are not evaluated. Conducting extended safety studies (2-4 weeks) monitoring organ function, biodistribution, and clearance kinetics, as well as assessing whether repeated dosing maintains efficacy without toxicity, would be very valuable for clinical translation. At a minimum, the acknowledgment of the need to conduct these studies should be mentioned in the manuscript. Similarly, practical considerations for human translation (manufacturing scalability, administration route optimization, regulatory pathway) are not discussed. Including a brief discussion on translational challenges and the proposed development pathway toward clinical application could be valuable for the study.

Response: Thank you for highlighting these critical aspects of clinical translation. Given that ALI is characterized by rapid onset and acute pathogenesis, our current

experimental design primarily focused on evaluating the short-term biocompatibility. However, we completely agree that establishing a comprehensive long-term safety profile and addressing translational logistics are mandatory prerequisites for clinical application.

Following your constructive suggestion, we have abstained from over-extrapolating our acute safety data and have incorporated a dedicated paragraph in the revised **Outlook section**. This new section explicitly acknowledges the necessity of extended safety evaluations (including biodistribution, clearance kinetics, and repeated dosing assessments over 2-4 weeks). These have been added in the **Outlook section**:

"Despite these promising acute outcomes, advancing K-SAC toward clinical translation necessitates addressing long-term safety and practical logistical hurdles. Future studies must establish extended safety profiles spanning 2 to 4 weeks to systematically evaluate chronic toxicity, tissue accumulation, clearance kinetics, and the safety of repeated dosing regimens. Bridging to human trials will further demand scalable manufacturing, optimized administration routes for intensive care (e.g., transitioning to aerosolized nebulization for ventilator compatibility), and navigating rigorous regulatory pathways. Ultimately, overcoming these translational challenges will help fully realize K-SAC as a versatile immunomodulatory platform, highlighting their tremendous potential in treating a broad range of inflammation-driven diseases, such as COVID-19, systemic sepsis, and inflammatory bowel disease (IBD)."

11. NAC is used as a positive control, but it is primarily an antioxidant rather than a specific pyroptosis inhibitor. Comparison with known pyroptosis inhibitors (e.g., disulfiram, necrosulfonamide) would better contextualize K-SACs' efficacy.

Response: We appreciate this excellent comment. At the initial stage of study design, NAC was chosen as a positive control because it has been extensively studied and is widely recognized for its potent antioxidant and anti-inflammatory properties[1]. Clinical studies have also confirmed the favorable safety profile of NAC and its ability to reduce the risk of moderate or severe exacerbations in patients with COPD of GOLD stage 1-2[2].

As you suggested, to further improve the rigor of this study, **we introduced disulfiram, a specific GSDMD inhibitor, as an additional positive control**. We performed pyroptosis-related experiments to verify the inhibitory effect of K-SACs on pyroptosis. In cellular experiments, disulfiram was administered following previously published protocols: 30 μM, 1 h before LPS stimulation. We found that disulfiram treatment significantly suppressed pyroptosis and the release of inflammatory factors in BMDMs (**Supplementary Figure 32**). K-SACs exerted similar inhibitory effects compared with disulfiram.

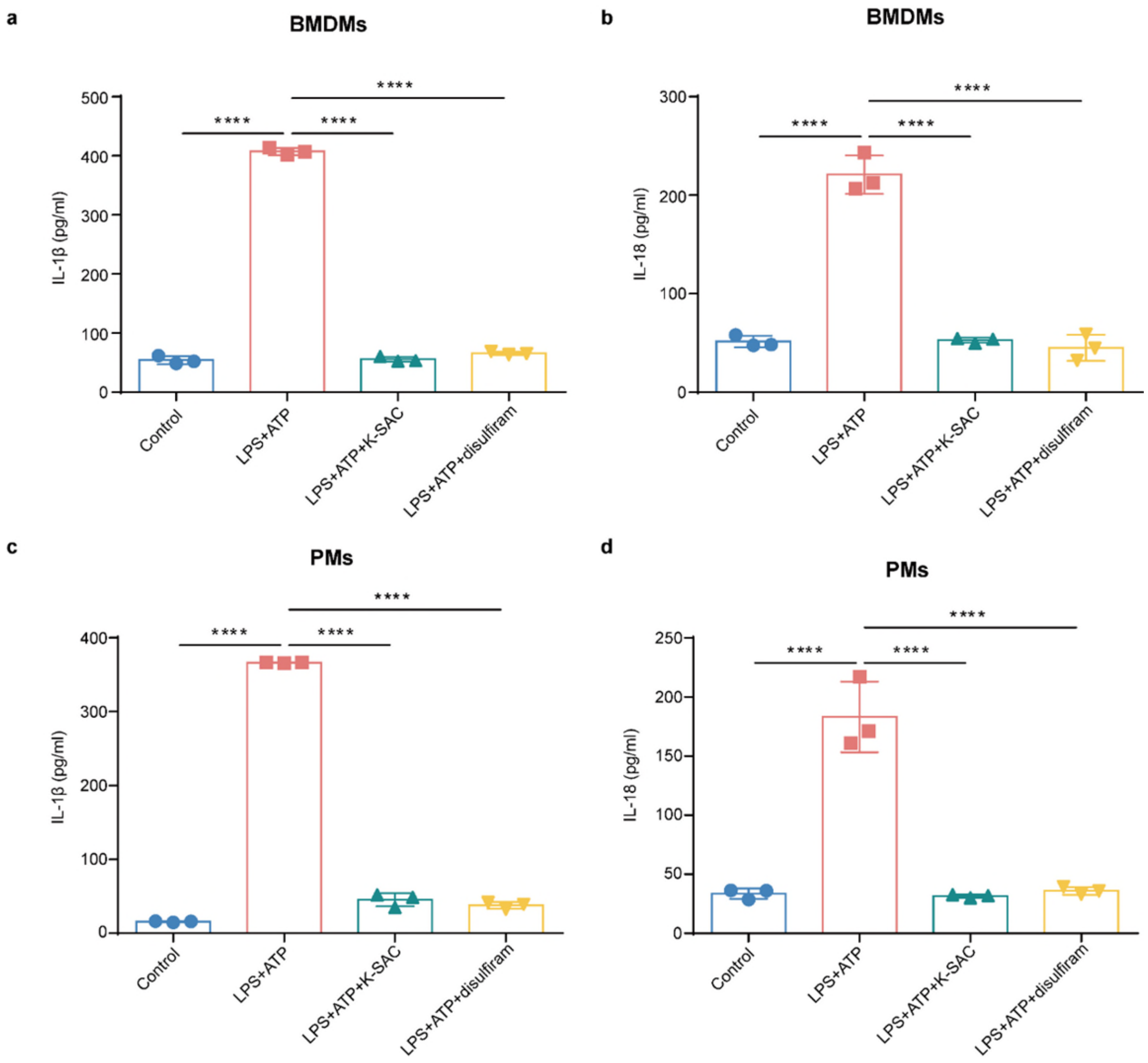

Supplementary Fig. 32. The pyroptosis inhibition efficacy of K-SAC compared with disulfiram.

a,b, IL-1β **(a)** and IL-18 **(b)** levels in BMDMs supernatants treated with K-SAC or disulfiram. **c,d,** IL-1β **(c)** and IL-18 **(d)** levels in PMs supernatants treated with K-SAC or disulfiram.

These results confirm that K-SACs can effectively inhibit macrophage pyroptosis. We added the part in **Results section 7**:

"The anti-inflammatory effects paralleled these antioxidant properties. K-SAC significantly decrease IL-1β and IL-18 release in supernatants of LPS- and ATP-stimulated BMDMs and PMs (Fig. 4k,l; Supplementary Fig. 30). We detected K-SAC's effects on pyroptosis in macrophages with or without GSDMD siRNA intervention (Fig. 4m,n; Supplementary Fig. 31) and in macrophages interfered with disulfiram (Supplementary Fig. 32). We found that K-SAC's protective effect is similar to that of disulfiram, and is GSDMD-dependent. These findings position K-SAC as a dual-action therapeutic agent that simultaneously neutralizes ROS and attenuates inflammation, offering promising potential for treating inflammatory lung diseases."

1. Santus P, Signorello JC, Danzo F, Lazzaroni G, Saad M, Radovanovic D. Anti-Inflammatory and Anti-Oxidant Properties of N-Acetylcysteine: A Fresh Perspective. *Journal of clinical medicine* **13**, (2024).
2. Zhou Y, *et al.* Effect of high-dose N-acetylcysteine on exacerbations and lung function in patients with mild-to-moderate COPD: a double-blind, parallel group, multicentre randomised clinical trial. *Nat Commun* **15**, 8468 (2024).

12. Some references lack complete information (page numbers).

Response: Thank you very much for your comment. We have added the complete information in references.

Response to Reviewer #2.

Comments from Reviewer #2:

This manuscript presents an interesting study proposing potassium single-atom catalyst as a new strategy to inhibit macrophage pyroptosis in acute lung injury. The work is notable for using a safe, physiologically abundant metal that exhibits antioxidant activity, highlighting its potential for further therapeutic development. While the study is promising, I have several specific comments and suggestions that could help improve the clarify and presentation of the manuscript.

Response: Thank you very much for the positive comment and kind suggestions. Please find the following detailed responses to your suggestions.

1. In the manuscript, the authors propose a structural model of the K center in the K-

SAC based on the EXAFS data fitting, followed by DFT optimization. In this model, K is coordinated by four N atoms and is displaced from the plane of the support. However, such a K site would be expected to adsorb gas molecule from the atmosphere (e.g. H_2O, CO_2, O_2), which should influence the first coordination sphere of K in the EXAFS spectrum.

EXAFS fitting showed the first K-N and second K-C shell at 2.72 and 3.04 Å, respectively. Could the authors justify the possibility of forming a K-C second coordination at 3.04 Å? Because K has a large ionic radius, its first-shell bond distances can reach up to 3.10 Å. Considering that K-O and K-C scattering paths cannot be reliably distinguished by EXAFS, the authors could modify EXAFS fitting by replacement of the K-C path with a K-O, where a K-O path can be rationalized as the first coordination shell formed upon adsorption of atmospheric gas molecules on the isolated K site. This interpretation does not alter the overall structural model of the K single center proposed in the manuscript but provides a more chemically reasonable description of the local K environment in the real catalyst.

Response: Thank you for the insightful and constructive comments on the EXAFS fitting and local coordination environment of K single atoms.

In our original EXAFS analysis, we assigned the scattering path at 3.04 Å to a K–C second shell, mainly based on the carbon support framework and the DFT-optimized structural model. We agree with you that K has a large ionic radius, and the bond distance of the first coordination shell can be up to ~3.10 Å. Meanwhile, EXAFS alone cannot unambiguously distinguish K–C and K–O scattering paths due to their similar backscattering amplitudes and phase shifts.

To provide a more chemically reasonable description, we have **revised the EXAFS fitting** by replacing the K–C path at 3.04 Å with a **K–O scattering path**, which can be reasonably attributed to the adsorption of atmospheric species (e.g., H_2O, CO_2, O_2) on the isolated K sites under ambient conditions (Supplementary Table 1). This adjustment does not change the main structural features of the K single-atom center, including the four N-coordinated configuration and the displacement of K from the carbon plane.

We have carefully revised the relevant description in the manuscript to emphasize that

the local K environment includes minor O-containing species from ambient adsorption, which is consistent with the chemical nature of highly electropositive K sites. All related figures and tables have been updated accordingly.

The reference (*Nano letters*, 2024. *Angewandte Chemie-International Edition*, 61(12): e202215157 (2022), and *Angewandte Chemie-International Edition*, 62(50): 110173 (2023)) has been cited as ref. [30, 32, 33].

The revisions to the revised manuscript are presented as follows:

"Furthermore, X-ray absorption fine structure (XAFS) measurements were conducted to investigate the local coordination environment of K atoms. The K K-edge X-ray absorption near-edge structure (XANES) spectra of K-SAC and the KCl reference sample are displayed in Fig. 1h. Comparison with bulk KCl, the absorption edge of K-SAC is slightly shifted to lower energy, which indicates that the K single atoms in K-SAC have a distinct electronic configuration and local chemical environment relative to the K(I) ions in KCl. In addition, the Fourier-transformed extended X-ray absorption fine structure (FT-EXAFS) spectrum of K-SAC features a dominant peak at ~1.99 Å in Fig. 1i, which is assigned to **K–N/O scattering**. By contrast, KCl and relevant literature data exhibit prominent peaks at ~2.7 Å (K–Cl) and ~3.9 Å (K–K), and the absence of these characteristic peaks in K-SAC confirms the atomic dispersion of K species in the carbon matrix, thus excluding the presence of crystalline KCl or metallic K aggregates[30, 32]. To quantitatively determine the exact coordination configuration of K atoms, least-squares fitting was performed on the EXAFS data. The optimal fitting model reveals that each K atom in K-SAC is coordinated by both N and O atoms, and specifically, the K coordination environment in K-SAC can be well simulated by a **K–N path** (coordination number ≈3.8, bond length 2.65 Å) **and a K–O path** (coordination number ≈3.6, bond length 2.72 Å) (Fig. 1j,k, Supplementary Fig. 7, and Supplementary Table 1). **The formation of K–O coordination is attributed to the spontaneous adsorption of ambient O-containing species (e.g., H_2O, CO_2, O_2) onto the electropositive isolated K sites, rather than representing a well-defined intrinsic coordination shell. Accordingly, the intrinsic structural motif of the K center is identified as K–N_4.** Moreover, wavelet transform (WT) analysis was used to further elucidate the

backscattering environment around K sites in Fig. 1l,m. The WT contour plot of K-SAC presents a distinct intensity maximum at ~2.6 Å^{-1} in k space, a feature characteristic of **K–N/O coordination,** which is clearly different from the signals of KCl and those reported in the literature with intensity maxima at 3.9 Å^{-1} for K–K and 2.7 Å^{-1} for K–Cl in k space[33]. Collectively, these XAFS analyses confirm that K species are anchored as isolated single atoms in the N-doped carbon framework."

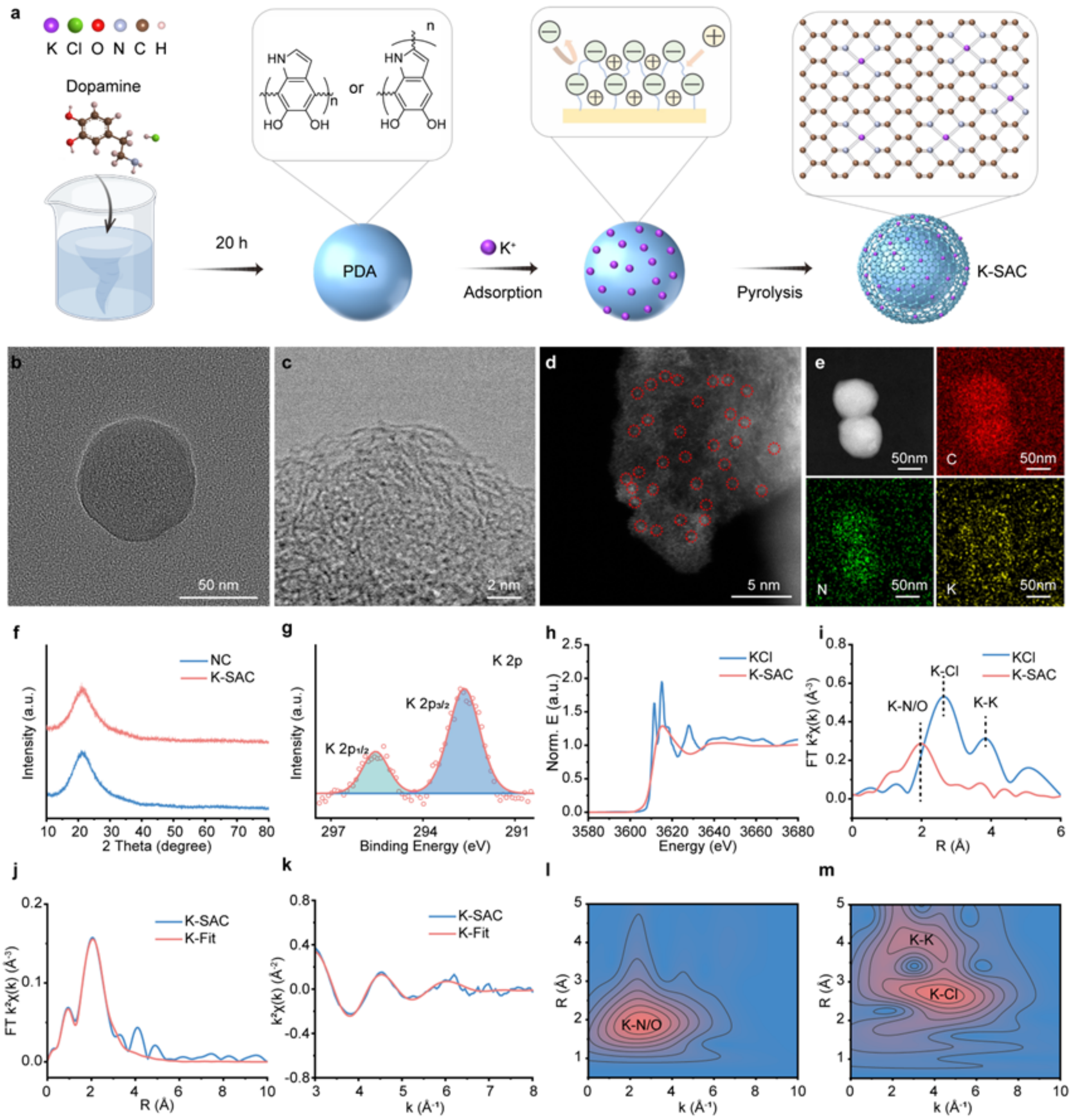

Fig. 1: Synthesis and characterization of K-SAC.

a, Schematic illustration of K-SAC synthesis. [Editorial note: by figdraw.com.] **b,** TEM image of K-SAC. **c,** BF-STEM image of K-SAC. **d,** AC HAADF-STEM image of K-SAC. **e,** HAADF-STEM image and corresponding EDS mappings of K-SAC: C (red), N (green), K (yellow). **f,** XRD patterns of K-SAC and NC. **g,** High-resolution K 2p

XPS of K-SAC. **h**, Normalized XANES spectra of K-SAC and KCl. **i,** FT-EXAFS results of K-SAC and KCl. **j-k,** EXAFS fitting for K-SAC in R space (**j**) and in k-space (**k**). **l-m,** Wavelet transformation of K-SAC (**l**) and KCl (**m**).

Supplementary Table 1. EXAFS data fitting results of Samples.

Sample	Path	CN^a	$R(\text{Å})^b$	$\sigma^2\ (\text{Å}^2)^c$	$\Delta E_0(\text{eV})^d$	*R* factor
KCl	K-Cl	6 (set)	3.125±0.038	0.010	-0.06	0.0127
	K-K	12 (set)	4.481±0.008	0.099		
K-SAC	K-N	3.8±0.1	2.654±0.066	0.018	-4.6	0.0020
	K-O	3.6±0.4	2.719±0.074	0.018		

30. Wu Y, Su R, Jiang W, et al. Nanozyme metabolism controls air pollution over an atomic potassium cyano site. *Nano letters*, (2024).

32. Peng S S, Shao X B, Gu M X, et al. Catalytically stable potassium single-atom solid superbases. *Angewandte Chemie-International Edition*, **61**(12): e202215157 (2022).

33. Guo N K, Xue H, Ren R, et al. S-block potassium single-atom electrocatalyst with k-n4 configuration derived from k+/polydopamine for efficient oxygen reduction. *Angewandte Chemie-International Edition*, **62**(50): e202312409 (2023).

2. The author should provide additional details regarding the EXAFS fitting procedure, including the k- and r-ranges, the specific k-weight(s) used in the analysis, and the method used to determine the amplitude reduction factor (S_0^2).

Response: Thank you so much for your thoughtful reminder.

The obtained XAFS data was processed in Athena (version 0.9.26) for background, pre-edge line and post-edge line calibrations. Then Fourier transformed fitting was carried out in Artemis (version 0.9.26).

The k^2 weighting k-range of 3 - ~8.3 Å^{-1} and R range of 1.0 – 3.0 Å were used for the fitting of K samples. For Wavelet Transform analysis, the $\chi(k)$ exported from Athena was imported into the Hama Fortran code. The parameters were listed as follows: R range, 0.5 - 6 Å, k range, 0 - 9 Å^{-1} for samples; k weight = 2; and Morlet function with $\kappa = 8$, $\sigma = 1$ was used as the mother wavelet to provide the overall distribution. S_0^2 was fixed to 0.96, according to the experimental EXAFS fit of KCl by fixing *CN* as the known crystallographic value. All additional details are provided in the revised Supplementary Information.

The revisions to the revised manuscript are presented as follows:

"[a]*CN*, coordination number; [b]*R*, the distance between absorber and backscatter atoms; [c]σ^2, the Debye Waller factor value; [d]ΔE_0, inner potential correction to account for the difference in the inner potential between the sample and the reference compound; *R* factor indicates the goodness of the fit. S_0^2 was fixed to 0.96, according to the experimental EXAFS fit of KCl by fixing *CN* as the known crystallographic value. Fitting conditions: *k* range: 3 – 8.3 Å^{-1}; *R* range: 1.0 – 3.0 Å; fitting space: R space; *k*-weight = 2. A reasonable range of EXAFS fitting parameters: $0.800 < S_0^2 < 1.000$; $CN > 0$; $\sigma^2 > 0$ Å^2; $|\Delta E_0| < 10$ eV; *R* factor < 0.02."

3. It is well known that K K-edge XANES spectra strongly depend on the K-containing phase. It would be valuable if the authors could provide a brief discussion of the obtained K XANES spectrum in comparison with literature data. Such an analysis would further support the absence of bulk K-containing phases (including highly dispersed species that may be undetectable by XRD) and strengthen the evidence for the formation of isolated K center in the catalyst.

Response: Thank you for this valuable suggestion. We fully agree that comparing the K K-edge XANES spectrum with reference data from literature is essential to clarify the local chemical environment and existing phases of potassium.

Accordingly, we have added a brief comparative analysis of the measured K K-edge XANES spectrum with typical reference spectra of bulk K foil, KCl, and highly dispersed atomic potassium species (K-N-C, K_1/G) reported in the literature (*Angew. Chem. Int. Ed.* **2023**, e202312409; *Angew. Chem. Int. Ed.* **2022**, e202215157)[1, 2]. The characteristic features in our spectrum show significant differences from those of crystalline or bulk K-containing phases. This comparison clearly supports the absence of bulk potassium-containing phases. Meanwhile, the spectral features are consistent with the characteristics of isolated potassium sites stabilized in the catalyst framework, thus providing more direct and convincing evidence for the formation of isolated K centers. We have revised the relevant section in the manuscript accordingly to include this discussion.

1. Guo N, et al. S-Block Potassium Single-atom Electrocatalyst with K-N(4) Configuration Derived from K(+) /Polydopamine for Efficient Oxygen Reduction. *Angew Chem Int Ed Engl* **62**, e202312409 (2023).
2. Peng SS, et al. Catalytically Stable Potassium Single-Atom Solid Superbases. *Angew Chem Int Ed Engl* **61**, e202215157 (2022).

The revisions to the revised manuscript are presented as follows:

"Furthermore, X-ray absorption fine structure (XAFS) measurements were conducted to investigate the local coordination environment of K atoms. The K K-edge X-ray absorption near-edge structure (XANES) spectra of K-SAC and the KCl reference sample are displayed in Fig. 1h. **Comparison with bulk KCl,** the absorption edge of K-SAC is slightly shifted to lower energy, which indicates that the K single atoms in K-SAC have a distinct electronic configuration and local chemical environment relative to the K(I) ions in KCl. In addition, the Fourier-transformed extended X-ray absorption fine structure (FT-EXAFS) spectrum of K-SAC features a dominant peak at ~1.99 Å in Fig. 1i, which is assigned to K–N/O scattering. **By contrast,** KCl and relevant literature data exhibit prominent peaks at ~2.7 Å (K–Cl) and ~3.9 Å (K–K), and the absence of these characteristic peaks in K-SAC confirms the atomic dispersion of K species in the carbon matrix, thus excluding the presence of crystalline **KCl or metallic K aggregates**[30, 32]. To quantitatively determine the exact coordination configuration of K atoms, least-squares fitting was performed on the EXAFS data. The optimal fitting model reveals that each K atom in K-SAC is coordinated by both N and O atoms, and specifically, the K coordination environment in K-SAC can be well simulated by a K–N path (coordination number ≈3.8, bond length 2.65 Å) and a K–O path (coordination number ≈3.6, bond length 2.72 Å) (Fig. 1j,k, Supplementary Fig. 7, and Supplementary Table 1). The formation of K–O coordination is attributed to the spontaneous adsorption of ambient O-containing species (e.g., H_2O, CO_2, O_2) onto the electropositive isolated K sites, rather than representing a well-defined intrinsic coordination shell. Accordingly, the intrinsic structural motif of the K center is identified as K–N_4. Moreover, wavelet transform (WT) analysis was used to further elucidate the backscattering environment

around K sites in Fig. 1l,m. The WT contour plot of K-SAC presents a distinct intensity maximum at ~2.6 $Å^{-1}$ in k space, a feature characteristic of K–N/O coordination, **which is clearly different from the signals of KCl and those reported in the literature** with intensity maxima at 3.9 $Å^{-1}$ for K–K and 2.7 $Å^{-1}$ for K–Cl in k space[33]. Collectively, these XAFS analyses confirm that K species are anchored as isolated single atoms in the N-doped carbon framework."

30. Wu Y, Su R, Jiang W, et al. Nanozyme metabolism controls air pollution over an atomic potassium cyano site. *Nano letters*, 2024.
32. Peng S S, Shao X B, Gu M X, et al. Catalytically stable potassium single-atom solid superbases. *Angewandte Chemie-International Edition*, **61**(12): e202215157 (2022).
33. Guo N K, Xue H, Ren R, et al. S-block potassium single-atom electrocatalyst with k-n4 configuration derived from k+/polydopamine for efficient oxygen reduction. *Angewandte Chemie-International Edition*, **62**(50): e202312409 (2023).

4. The FTIR data (Sup. Fig.5) are not entire clear. First, it would be helpful if the author could provide the exact values of the C-N and C=N stretching modes and cite relevant literature where similar bands have been observed. Additionally, it is not clear what specific information this experiment is intended to provide. For instance, should the introduction of K on the support alter the ratio of C-N to C=N bonds? A similar issue arises with the XPS data (lines 157-160 in the main text, Sup. Fig.7). Is it unclear what evidence indicates the formation of different types of N on the surface of K-SACs and NC, and how the introduction of K affects the distribution of these N species. Based on the data presented, it would be helpful if the authors could provide clearer conclusions drawn from these measurements.

Response: Thank you very much for the professional comments and constructive suggestions. We have carefully revised the manuscript to address these points.

(1) Regarding the FTIR data: We have clarified the exact wavenumbers of the C–N and C=N stretching modes with relevant literature citations, in the revised Supplementary Fig. 5 and the main text. Specifically, we report that the FTIR spectra of NC and K-SAC show bands at 1235 cm^{-1} and 1605 cm^{-1}, assigned to the C–N and C=N stretching vibrations in N-doped carbon frameworks, respectively[1, 2]. Similar results for N-doped

carbon materials have been reported in the literature, and the corresponding references have been incorporated. These results **clearly confirm the successful formation of the N-doped carbon framework** in both samples.

1. Wu Y, et al. Nanozyme Metabolism Controls Air Pollution over an Atomic Potassium Cyano Site. *Nano letters*, (2024).
2. Han Y, et al. Modulating the Coordination Environment of Carbon-Dot-Supported Fe Single-Atom Nanozymes for Enhanced Tumor Therapy. *Small* **20**, 2306656 (2023).

(2) Regarding the XPS data: To **precisely quantify the impact of K incorporation on the N species distribution**, we performed a systematic deconvolution of the N 1s spectra for both K-SAC and NC using consistent fitting parameters. The detailed deconvolution results are presented in Supplementary Fig. 8 and Supplementary Table 2. The N 1s spectra are deconvoluted into pyridinic N (~398.3 eV), pyrrolic N (~399.1 eV), graphitic N (~401.0 eV), and oxidized N (~402.7 eV). Quantitatively, **the content of pyridinic N increases from 15.9% (NC) to 19.2% (K-SAC)**, while those of pyrrolic and graphitic N decrease slightly. This enrichment suggests that the introduction of K atoms **selectively stabilizes pyridinic N sites.** Consistent with the literature on single-atom catalysts, the introduction of metal atoms during thermal treatment tends to stabilize pyridinic N sites through preferential coordination (forming thermodynamically stable pyridinic N-metal motifs), while less stable pyrrolic species are consumed or converted[1, 2].

1. Wang L, et al. A polymer tethering strategy to achieve high metal loading on catalysts for Fenton reactions. *Nat Commun* **14**, 7841 (2023).
2. Kumar A, et al. Moving beyond bimetallic-alloy to single-atom dimer atomic-interface for all-pH hydrogen evolution. *Nat Commun* **12**, 6766 (2021).

The reference (*Nano letters*, 2024; *Small* **20**, 2306656 (2023)) has been cited as ref. [30, 31]. The reference (*Nat. Commun.* **12**, 6766 (2021), *Nat. Commun.* **14**, 7841 (2023), *Carbon* **162**, 528-544 (2020), *Nat. Commun.* **14**, 4430 (2023)) has been cited as ref. [34-37].

The revisions to the revised manuscript are presented as follows:

"Fourier transform infrared spectroscopy (FTIR) spectra reveal that K-SAC possess similar vibrational features to those of NC in the 1200-1600 cm^{-1} region, assignable to stretching modes of C–N (1235 cm^{-1}) and C=N (1605 cm^{-1}) bonds (Supplementary Fig. 5)[30, 31]. These results confirm the successful formation of the N-doped carbon framework in both samples."

"High-resolution N 1s spectra of the samples were also recorded and deconvoluted into four components: pyridinic N (398.3 eV), pyrrolic N (399.1 eV), graphitic N (401.0 eV), and oxidized N (402.7 eV) (Supplementary Fig. 8). Detailed statistical analysis reveals that the pyridinic N content increases from 15.9% in NC to 19.2% in K-SAC (Supplementary Table 2). This evolution suggests that **the introduction of K atoms promotes the stabilization of pyridinic N sites**. This phenomenon aligns with the formation mechanism of single-atom catalysts, where metal atoms preferentially coordinate with pyridinic N at defect sites to form thermodynamically stable metal-N motifs[34-37]. Collectively, these results suggest that the K species are effectively anchored via coordination with pyridinic N, thereby defining the intrinsic atomic structure of the K-SAC."

30. Wu, Y. et al. Nanozyme Metabolism Controls Air Pollution over an Atomic Potassium Cyano Site. Nano letters (2024).
31. Han, Y. et al. Modulating the Coordination Environment of Carbon-Dot-Supported Fe Single-Atom Nanozymes for Enhanced Tumor Therapy. Small 20, 2306656 (2023).
34. Kumar, A. et al. Moving beyond bimetallic-alloy to single-atom dimer atomic-interface for all-pH hydrogen evolution. Nat. Commun. 12, 6766 (2021).
35. Wang, L. et al. A polymer tethering strategy to achieve high metal loading on catalysts for Fenton reactions. Nat. Commun. 14, 7841 (2023).
36. Ayiania, M. et al. Deconvoluting the XPS spectra for nitrogen-doped chars: An analysis from first principles. Carbon 162, 528-544 (2020).
37. Peng, W. et al. Facilitating two-electron oxygen reduction with pyrrolic nitrogen sites for electrochemical hydrogen peroxide production. Nat. Commun. 14, 4430 (2023).

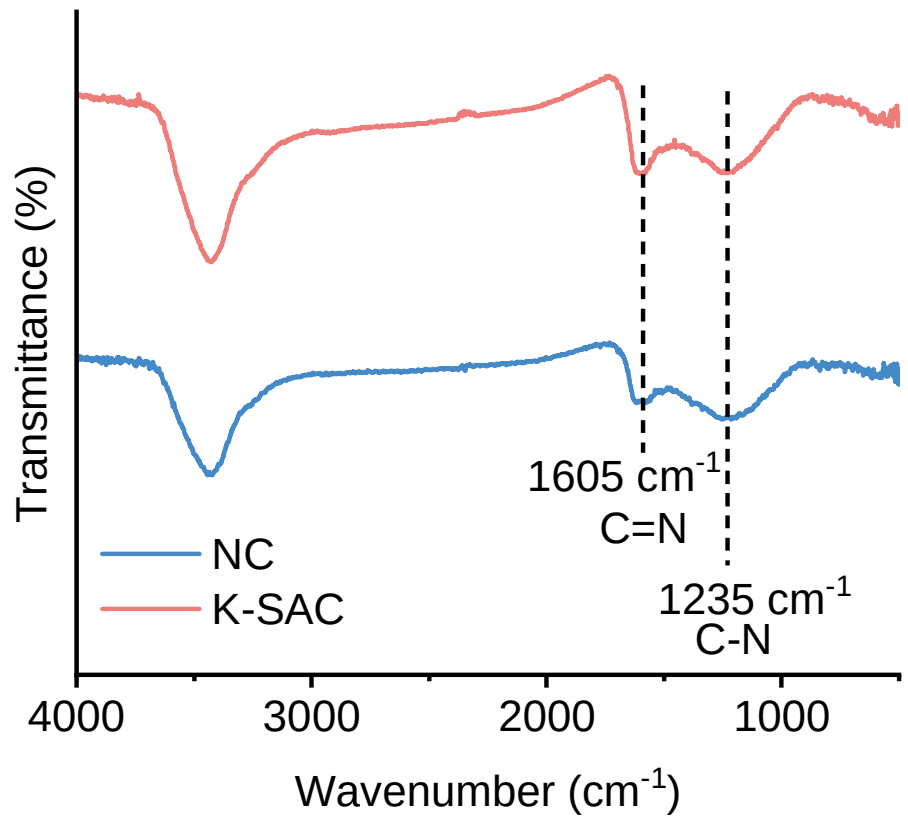

Supplementary Fig. 5. FTIR Spectra of K-SAC and NC.

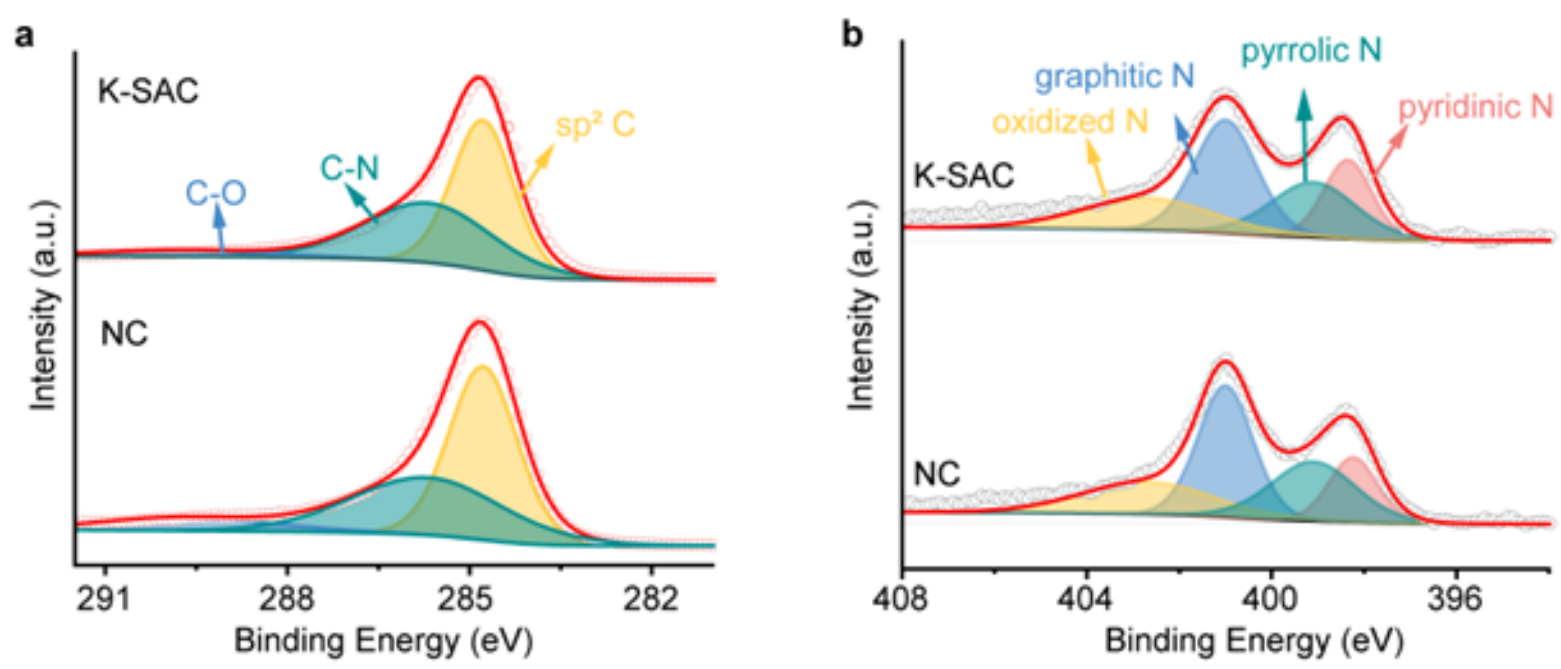

Supplementary Fig. 8. High-resolution XPS results.

a, High-resolution C 1s spectra of K-SAC and NC. **b,** High-resolution N 1s spectra of K-SAC and NC.

Supplementary Table 2. The proportion of different nitrogen species for the two catalysts from XPS results.

Sample	oxidized N	graphitic N	pyrrolic N	pyridinic N
Peak position (eV)	402.7	401.0	399.1	398.3
NC	21.6	36.8	25.7	15.9
K-SAC	21.2	35.9	23.7	19.2

5. There are several typos and inaccuracies throughout the manuscript:

- Line 99 "…potassium-based single-atom catalysts (K-SACs)..", but this study

presents only one catalyst. The abbreviation “K-SACs” may be confusing, suggesting multiple catalysts were studied. It is suggested to change K-SAC.

Response: Thank you very much for your valuable input. As suggested, we have revised the abbreviation "K-SACs" to "K-SAC", and the abbreviation "K-SAC" has been used consistently throughout the manuscript.

• Line 119 “As a control, K-free NC was also prepared…”, abbreviation NC is not clarified.

Response: Thank you very much for the kind reminder. Based on your suggestion, we have now clarified "NC" as its full name (N-doped carbon) at its first appearance in the revised manuscript to avoid confusion.

The revisions to the revised manuscript are presented as follows:

“For comparison, a K-free N-doped carbon (NC) was prepared by the same procedure as K-SAC without the addition of KCl.”

• Figures: The sequence and referencing of figures in the manuscript are inconsistent. For example, in the text, Supplementary Fig. 4 is cited before Supplementary Fig.3. Additionally, some figures are included in the Supplementary Information but are not mentioned in the main text, such as Supplementary Fig. 8 and 11.

Response: Thank you very much for your valuable suggestion. We have carefully checked the entire manuscript and revised the citation order of supplementary figures (e.g., adjusted the citation sequence of Supplementary Fig. 3 and 4 to be consistent with their logical order). We have also carefully checked and added descriptions of figures in the supplementary information in the main text, to ensure the consistency between the main text and supplementary information.

• Figure 1 (line 496): The caption “XPS spectrum” should be corrected to “XP spectrum”; similar errors are present in other figure captions with XP spectra.

Response: Thank you for your kind suggestion. We have corrected the caption “XPS spectrum” to “High-resolution K 2p XPS of K-SAC.” in Figure 1 and revised all other

figure captions with similar errors consistently throughout the manuscript.

• Supplementary Fig. 25: There is an error in the labeling of panels (a-f).

Response: Thank you for your kind suggestion. We have corrected the figure lettering.

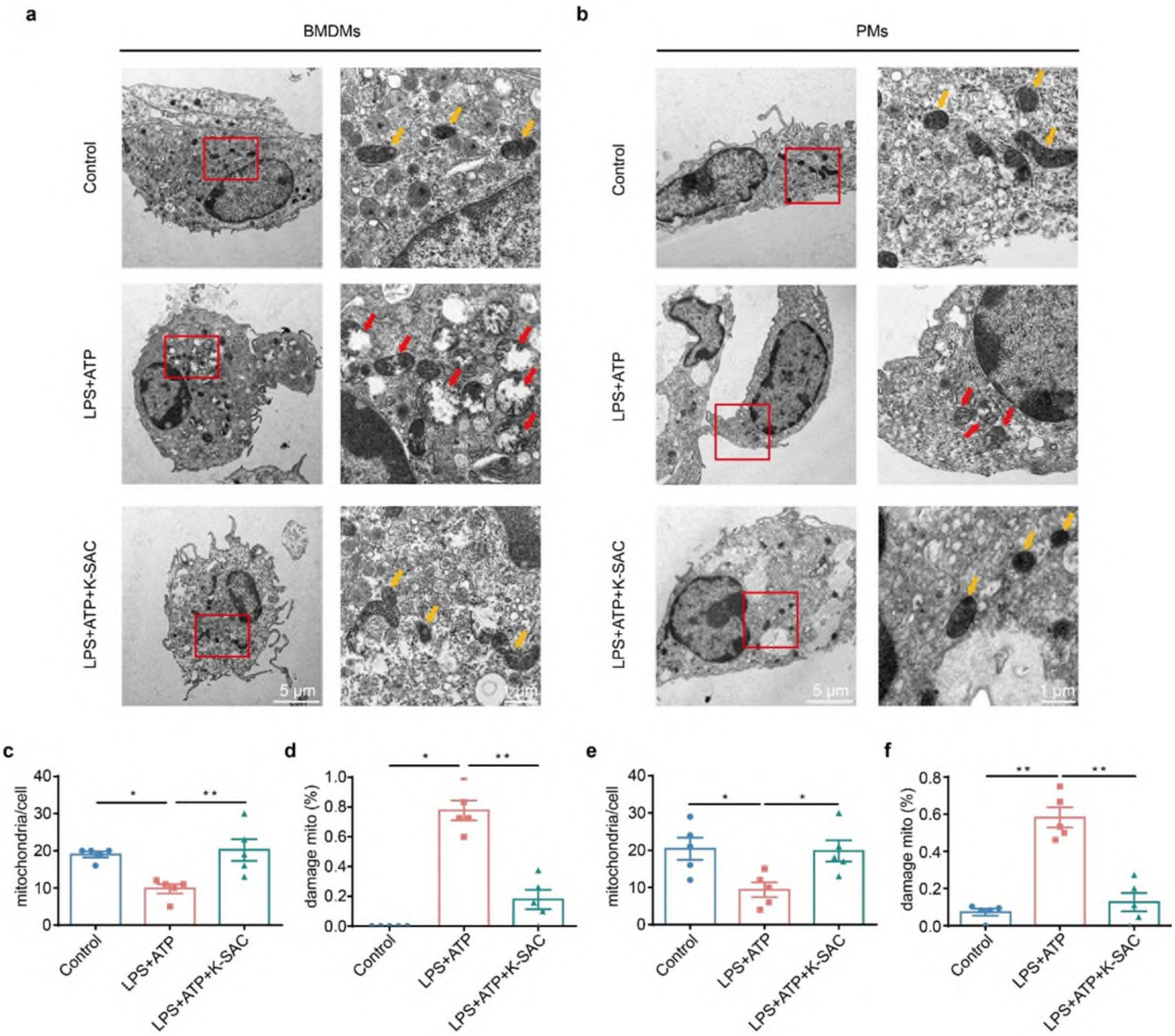

Supplementary Fig. 28. K-SAC alleviate mitochondria damage.

a,b, Representative images of TEM analysis of BMDMs **(a)** and PMs **(b)**. The right panels show higher magnification of the boxed area in the left panels. The yellow arrows pointed the normal mitochondria; red arrows pointed swelling mitochondria (Scale bar, upper: 5 μm, lower: 1 μm). **c-f,** Mitochondrial (mito) number and percentage of damaged mitochondria of BMDMs **(c,d)** and PMs **(e,f)** were quantified.

Response to Reviewer #3.

Comments from Reviewer #3:

The study describes the therapeutic effects of a novel potassium single-atom catalysts

(K-SACs), which exerts potent catalytic activity with minimal metal toxicity, on sepsis-induced acute lung injury. The authors have adopted both in vitro and in vivo models and offered a comprehensive analysis of potential mechanism of pyroptosis in macrophages also using RNA seq-analysis. The chemical structure and activity of the enzyme appears well characterized with state-of-art technologies. The in vitro data appear robust, well-conceived and supportive of the conclusions. However, there are several concerns about the in vivo models of ALI.

Response: Thank you very much for the insightful comments and suggestions. We have revised the manuscript carefully and provided detailed responses below in a point-by-point manner according to your suggestions.

Major comments:

1. Methods: Cecal ligation and puncture model. Details of the animal model of cecal ligation and puncture are missing (size of needle, number of punctures, use of anesthesia, time of treatment, and time of sacrifice and organ harvesting). The lack of this information makes difficult the interpretation of the severity and reliability of the model. Also, it is not clear why the authors used only male mice. A rationale for exclusion of female mice should be provided.

Response: Thank you for the professional comments.

For your first comment: Cecal ligation and puncture model. Details of the animal model of cecal ligation and puncture are missing (size of needle, number of punctures, use of anesthesia, time of treatment, and time of sacrifice and organ harvesting). The lack of this information makes difficult the interpretation of the severity and reliability of the model.

We sincerely apologize for omitting the details of the animal model of cecal ligation and puncture. We have added the details of the animal model of cecal ligation and puncture in **Methods section 8**.

The reference (*Nat Protoc* **4**, 31-36 (2009)) has been cited as ref. [55].

The details are as follows:

"8. ALI Mouse Models and Treatment

For LPS-induced acute lung injury, 10 mg/kg LPS was injected intraperitoneally. Mice were administered K-SAC (1 mg/kg in PBS) or NAC (360 mg/kg in PBS) at 48 h before, 24 h before, and concurrently with LPS challenge. The sepsis-induced ALI model was induced by cecal ligation and puncture (CLP) as standardized previously[55]. Mice were anesthetized with 2,2,2-tribromoethanol (400 mg/kg, i.p.). After a midline abdominal incision, the cecum was exposed and 50% of the distal segment was ligated with 4-0 silk suture without blocking the ileocecal valve. A single through-and-through puncture was made with a 21-gauge needle, and a small volume of feces was gently extruded. The cecum was repositioned and the incision was closed layer-by-layer. Sham-operated mice underwent the same procedure without ligation and puncture. All animals received subcutaneous fluid resuscitation (prewarmed saline, 5 mL/100 g) postoperatively. 24 h after lung injury, mice were sacrificed for further analysis."

55. Rittirsch, D., Huber-Lang, M.S., Flierl, M.A. & Ward, P.A. Immunodesign of experimental sepsis by cecal ligation and puncture. *Nat Protoc* **4**, 31-36 (2009).

For your second comment: it is not clear why the authors used only male mice. A rationale for exclusion of female mice should be provided.

We appreciate for this important point regarding sex as a biological variable. male C57BL/6 mice were exclusively utilized to minimize the substantial intra-group biological variability introduced by the female estrous cycle. It is well-documented that fluctuations in female sex hormones, particularly estrogen, significantly modulate innate immune responses, macrophage activation, and the baseline severity of LPS- or sepsis-induced acute lung injury[1, 2].

By employing a single-sex (male) cohort, we aimed to standardize the baseline inflammatory threshold and accurately isolate the specific therapeutic efficacy of K-SAC. However, we fully acknowledge that therapeutic responses may exhibit sex dimorphism. To address your concern, we have now explicitly provided this rationale in the Methods section, committing to dual-sex evaluations in our forthcoming translational studies.

1. Speyer CL, *et al.* Regulatory effects of estrogen on acute lung inflammation in mice. *Am J Physiol Cell Physiol* **288**, C881-890 (2005).
2. Chai X, *et al.* Oestrogen sulfotransferase ablation sensitizes mice to sepsis. *Nat Commun* **6**, 7979 (2015).

We have added the rationale for exclusion of female mice in **Methods section 7**:

"**7. Animal Studies**

Male C57BL/6 mice (8 weeks old) were obtained from Joint Ventures Sipper BK Experimental Animals (Shanghai, China) and maintained under specific-pathogen-free (SPF) conditions at 22 ± 1°C with 50 ± 10% humidity and a 12-hour light/dark cycle. Male mice were exclusively utilized in the current study to eliminate the confounding effects of estrous cycle-driven hormonal fluctuations on baseline immune responses and to reduce intra-group variability in the severity of lung inflammation. All procedures followed the NIH Guide for the Care and Use of Laboratory Animals (Publication No. 8023, revised 1978) with approval of the Scientific Investigation Board of Shanghai Pulmonary Hospital (Shanghai, China)."

2. Methods and title mismatch: In both in vitro and in vivo experiments, the authors adopted a pretreatment with the K-SACs. The authors should provide a rationale for this pre-treatment and comment on the pharmacokinetics of the catalyst. Also, the authors should test a post-treatment in ALI models to really demonstrate that catalyst initiate immunotherapy, as the authors claim in the title.

Response: We appreciate your professional comments. We appreciate your insightful comment on the suitability of our title. We fully acknowledge the concern that "Initiate" could imply a specific clinical treatment timeline. However, we respectfully prefer to retain the original title for the following reason:

In molecular and cellular biology, "initiate" is commonly used to describe a catalyst or molecular event that triggers a defined intracellular biological program. In this study, K-SAC initiates the restoration of immune homeostasis by inhibiting macrophage pyroptosis and activating ESCRT-mediated membrane repair (Figure 5). Thus,

"Catalysts Initiate Acute Lung Injury Immunotherapy" emphasizes that the material itself elicits an immunoprotective cellular response, rather than defining a specific dosing schedule.

Our current administration protocol (pre- and co-treatment) primarily demonstrates a prophylactic and early-intervention effect rather than a therapeutic reversal of an established disease. The pre-treatment regimen was strategically chosen due to the extremely rapid and highly aggressive onset of the LPS- and CLP-induced ALI models. In these models, the initial massive burst of reactive oxygen species (ROS) and subsequent macrophage pyroptosis occur within hours. Pre-treatment ensures that K-SAC are pre-positioned in the alveolar microenvironment to scavenge this initial ROS surge, allowing us to mechanistically isolate and observe the inhibition of the primary pyroptotic cascade before irreversible tissue necrosis occurs.

We have explicitly stated this rationale in the revised **Results section 9**:

"Having established the anti-inflammatory effects of K-SAC in vitro, we next examined the therapeutic potential in mouse models of ALI. In LPS- and CLP-induced ALI, pathological changes and the initial ROS burst develop rapidly. To efficiently scavenge early oxidative stress and assess early blockade of the pyroptotic cascade, we used prophylactic and co-treatment administration. This strategy ensures sufficient local bioavailability of K-SAC in the alveolar microenvironment before irreversible severe tissue damage occurs."

Following intranasal administration, K-SAC are directly delivered to the pulmonary space, establishing high local bioavailability and rapid cellular uptake by alveolar macrophages. This local delivery bypasses systemic circulation, which justifies our dosing schedule. However, comprehensive systemic pharmacokinetics profiling (absorption, distribution, metabolism, and excretion) remains to be fully elucidated. As suggested, we administered K-SAC inhalation three times at 1, 3, and 5 hours after intraperitoneal LPS injection, and evaluated lung tissue injury via H&E staining (**Supplementary Figure 17e,f**). We found that post-treatment also alleviated lung injury, although the protective effect was weaker than that of pre-treatment.

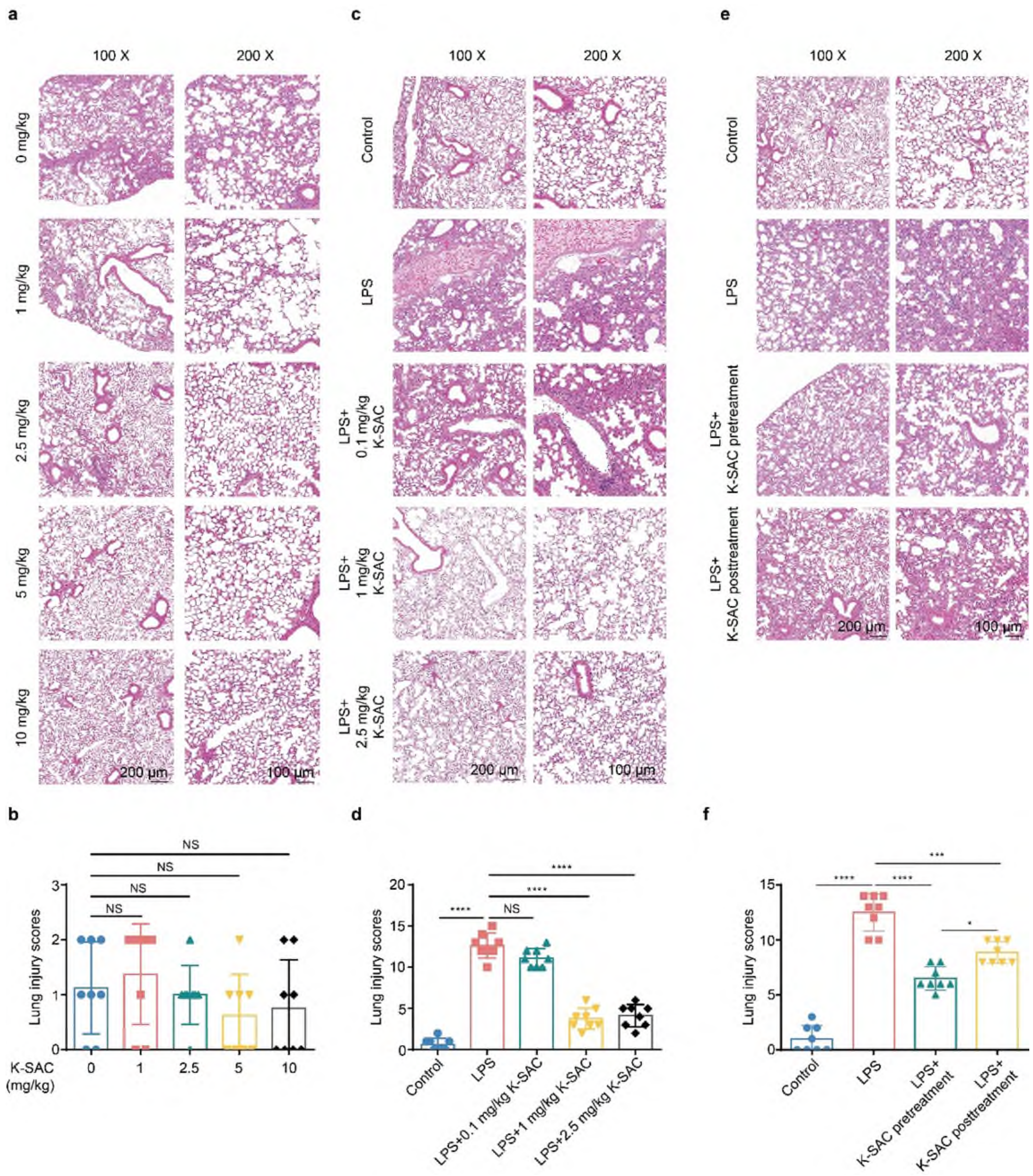

Supplementary Fig. 17. Pulmonary safety evaluation and efficacy detection of different doses of K-SAC.

a,b, Representative H&E staining of mouse lung tissues following nasal inhalation of different doses of K-SAC (1 mg/kg, 2.5 mg/kg, 5 mg/kg, 10 mg/kg) **(a)** and lung injury score **(b)** (scale bar: left, 200 μm; right, 100 μm). **c,d,** Representative H&E staining of mouse lung tissues following LPS injection and nasal inhalation of different doses of K-SAC (0.1 mg/kg, 1 mg/kg, 2.5 mg/kg) **(c)** and lung injury score **(d)** (scale bar: left, 200 μm; right, 100 μm). **e,f,** Representative H&E staining of mouse lung tissues following nasal inhalation of K-SAC (1 mg/kg) before or after LPS injection **(e)** and lung injury score **(f)** (scale bar: left, 200 μm; right, 100 μm).

3. Methods: Lung injury score. Lung injury score is based on a scale of severity but the histological criteria (i.e, hemorrhage, cell infiltration, mucin formation, alveolar patency and others) are not described. Although the author state that "The degree of lung injury was estimated based on the previously reported semiquantitative scoring standard", no reference is provided.

Response: We sincerely apologize for omitting the relevant citation and detailed scoring criteria. We have clearly stated in the revised manuscript that the histopathological quantitative evaluation of lung injury was strictly conducted in accordance with previous study.

The reference (*Shock (Augusta, Ga.)* **53**, 103-113 (2020)) has been cited as ref. [9].

The detailed scoring criteria are revised in **supplementary methods section 9** as follows:

"The degree of lung injury was estimated based on semiquantitative scoring standard established in our previous studies[9]. The lung injury score was assessed by two blinded pathologists. The histologic features considered for scoring included interstitial/intra-alveolar edema, hemorrhage, inflammation, and alveolar septal thickening/hyaline membrane formation. On a 5-point scale, each item was given a grade of 0 (little), 1 (mild), 2 (moderate), 3 (severe), or 4 (maximum), correspondingly. The total of all the variables was used to calculate the lung injury score (total score, 0-16)."

9. Liu, M. et al. α-Ketoglutarate Modulates Macrophage Polarization Through Regulation of PPARγ Transcription and mTORC1/p70S6K Pathway to Ameliorate ALI/ARDS. *Shock (Augusta, Ga.)* **53**, 103-113 (2020).

4. Results: Figure 3. While architecture of lungs appears improved in the groups of animals pre-treated with K-SACs, it appears that there is still a discrete neutrophil infiltration. The authors should quantified cell infiltration/neutrophils in the lung.

Response: We acknowledge the reviewer's accurate observation regarding the residual neutrophil infiltration in the K-SAC-treated groups. To address this comment and provide an objective evaluation of the inflammatory cell burden, we have now

quantified neutrophil infiltration in the lung tissues.

We performed immunohistochemical (IHC) staining for MPO in the lung tissues of control, LPS, LPS+K-SAC, and LPS+NAC groups. The quantitative results, which have been added to the revised manuscript (**Supplementary Figure 21c,d**), demonstrate that K-SAC treatment significantly reduced the MPO-positive area compared to the untreated ALI group.

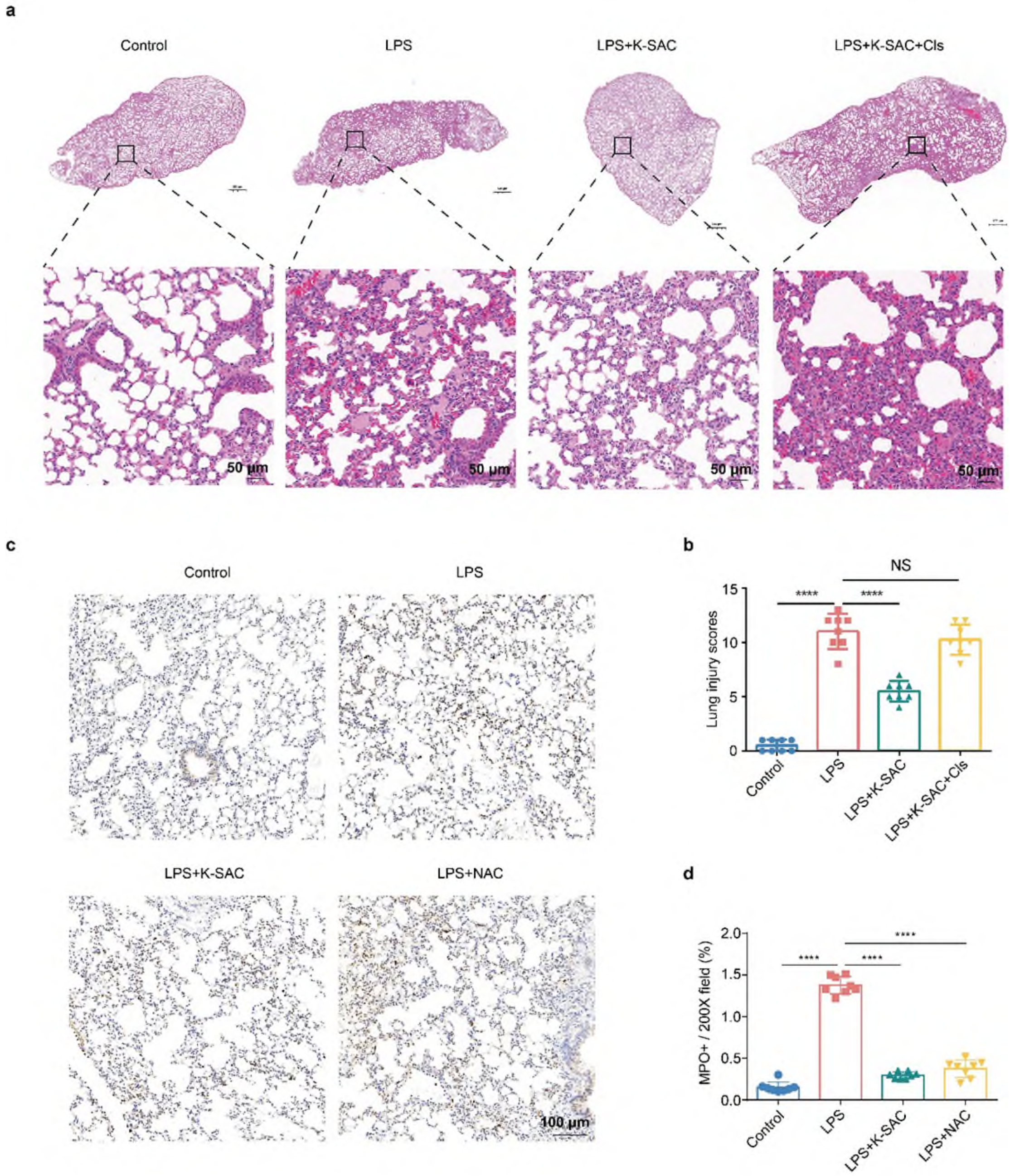

Supplementary Fig. 21. Macrophages play a critical role in the alleviation of lung injury by K-SAC.

a,b, The therapeutic effect of K-SAC was significantly attenuated after intraperitoneal macrophage depletion. **c,d,** Immunohistochemistry detection of neutrophil myeloperoxidase (MPO) in lung tissue.

These are revised in **Results section 5** as follows:

"To investigate the role of macrophages in the therapeutic effect of K-SAC against ALI, we depleted macrophages in lung tissue using clodronate liposomes and examined whether the therapeutic efficacy of K-SAC was altered. We found that the therapeutic effect of K-SAC on lung injury was markedly attenuated following macrophage depletion (Supplementary Fig. 21a,b), demonstrating that macrophages are essential for K-SAC to ameliorate acute lung injury. Although neutrophils were also significantly decreased after K-SAC treatment (Supplementary Fig. 21c,d), given that our experimental data confirmed K-SAC was predominantly enriched in macrophages, we speculate that this change may be attributed to reduced chemokine secretion following the resolution of inflammation by macrophages."

Reviewer #1 (Remarks to the Author):

The authors have addressed all the points raised by me and the other reviewers in a very detailed and comprehensive manner. In particular, the inclusion of new experiments has helped to clarify several mechanisms involved in the therapeutic effect of the nanosystem (K-SAC), thereby strengthening the manuscript.

From my perspective, the manuscript has been satisfactorily revised, and I consider it suitable for publication in its current form.

Response: Thank you very much for the positive comment.

Reviewer #2 (Remarks to the Author):

The authors of the manuscript by Lu et al. have provided clear and appropriate responses to the comments raised. The corresponding revisions have been implemented consistently and have improved the overall clarity and presentation of the manuscript. The main issues have been adequately addressed, and no further major concerns remain. In its current form, the manuscript can be considered suitable for publication.

Response: Thank you very much for the kind suggestion.

Reviewer #3 (Remarks to the Author):

Thank you for your careful and thoughtful responses to the review comments. The revisions effectively address the prior concerns. I have no further comments.

Response: Thank you for your kind positive feedback.
