# Supplementary material for: A potassium-based single-atom catalyst enables acute lung injury immunotherapy in mice by inhibiting macrophage pyroptosis
Source: Nat Commun. 2026 Aug 4;17:9400. doi: 10.1038/s41467-026-76331-8 (PMC13534642; doi:10.1038/s41467-026-76331-8)
Supplement: Supplementary file 5 [file 41467_2026_76331_MOESM3_ESM.docx]

**Supplementary Data 1.** The differentially expressed lipids after K-SAC treatment.

| **Lipid** | **CalcMass** | **RT(min)** | **Formula** | **Ion** | **VIP** | **p** | **FC(L+A/K)** |
| --- | --- | --- | --- | --- | --- | --- | --- |
| SM(t27:3) | 616.4216 | 1.67 | C32H61N2O7P1 | M+Na | 1.01 | 0.02 | 66.90 |
| PC(O-23:1) | 591.4264 | 1.69 | C31H62N1O7P1 | M+H | 1.35 | 0.01 | 21.19 |
| Hex1Cer(t25:1) | 589.419 | 1.66 | C31H59N1O9 | M+H | 5.19 | 0.01 | 19.54 |
| LPC(O-21:2) | 547.4002 | 1.73 | C29H58N1O6P1 | M+H | 1.14 | 0.01 | 18.96 |
| DG(2:0_18:2) | 396.2876 | 1.89 | C23H40O5 | M+NH4 | 1.71 | 0.00 | 4.73 |
| PC(16:1_16:1) | 729.5309 | 6.26 | C40H76N1O8P1 | M+H | 1.07 | 0.03 | 0.56 |
| PC(34:2) | 757.5622 | 6.97 | C42H80N1O8P1 | M+H | 3.56 | 0.05 | 0.54 |
| PC(O-16:2_20:4) | 763.5516 | 7.65 | C44H78N1O7P1 | M-CH3 | 1.70 | 0.03 | 0.49 |
| PC(O-15:2_20:4) | 749.5359 | 7.52 | C43H76N1O7P1 | M+H | 1.33 | 0.03 | 0.47 |
| PC(18:0_20:4) | 809.5935 | 8.59 | C46H84N1O8P1 | M+H | 1.10 | 0.03 | 0.44 |
| PC(18:1_18:1) | 785.5935 | 8.60 | C44H84N1O8P1 | M+Na | 1.07 | 0.04 | 0.44 |
| PC(16:0_22:6) | 805.5622 | 6.41 | C46H80N1O8P1 | M+H | 1.85 | 0.03 | 0.42 |
| FA(18:0) | 284.2715 | 3.89 | C18H36O2 | M-H | 6.85 | 0.01 | 0.40 |
| PC(17:0_18:2) | 771.5778 | 7.10 | C43H82N1O8P1 | M+HCOO | 1.18 | 0.02 | 0.40 |
| PC(16:0_18:1) | 759.5778 | 7.96 | C42H82N1O8P1 | M+H | 8.60 | 0.02 | 0.39 |
| SM(d18:1_24:1) | 812.6771 | 11.75 | C47H93N2O6P1 | M+H | 1.25 | 0.03 | 0.39 |
| PC(31:1) | 717.5309 | 6.49 | C39H76N1O8P1 | M+Na | 1.12 | 0.02 | 0.39 |
| PE(O-16:1_20:4) | 723.5203 | 7.56 | C41H74N1O7P1 | M-H | 2.52 | 0.01 | 0.39 |
| PC(38:5) | 807.5778 | 6.76 | C46H82N1O8P1 | M+H | 3.15 | 0.02 | 0.38 |
| PC(36:4) | 781.5622 | 7.95 | C44H80N1O8P1 | M+H | 1.25 | 0.02 | 0.37 |
| **Lipid** | **CalcMass** | **RT(min)** | **Formula** | **Ion** | **VIP** | **p** | **FC(L+A/K)** |
| PC(O-13:1_20:4) | 723.5203 | 7.52 | C41H74N1O7P1 | M+H | 1.72 | 0.02 | 0.37 |
| PE(20:4_18:0) | 767.5465 | 8.49 | C43H78N1O8P1 | M-H | 1.61 | 0.02 | 0.36 |
| FA(18:2) | 280.2402 | 2.22 | C18H32O2 | M-H | 1.47 | 0.03 | 0.36 |
| PE(O-18:2_20:4) | 749.5359 | 7.58 | C43H76N1O7P1 | M-H | 2.01 | 0.01 | 0.36 |
| LPA(19:1) | 450.2746 | 0.93 | C22H43O7P1 | M+Na | 2.85 | 0.05 | 0.34 |
| PC(O-32:0) | 719.5829 | 8.80 | C40H82N1O7P1 | M+H | 1.29 | 0.04 | 0.34 |
| PC(O-34:1) | 745.5985 | 8.79 | C42H84N1O7P1 | M+H | 2.85 | 0.02 | 0.34 |
| PE(18:0_20:4) | 767.5465 | 8.66 | C43H78N1O8P1 | M+H | 1.99 | 0.01 | 0.34 |
| PC(14:0_16:0) | 705.5309 | 7.06 | C38H76N1O8P1 | M+H | 2.06 | 0.02 | 0.34 |
| PC(O-13:1_22:4) | 751.5516 | 8.85 | C43H78N1O7P1 | M+H | 1.00 | 0.01 | 0.34 |
| PE(20:4_18:0) | 767.5465 | 8.24 | C43H78N1O8P1 | M+H | 3.22 | 0.01 | 0.33 |
| CerPE(d39:4) | 724.5519 | 6.71 | C41H77N2O6P1 | M+H | 1.10 | 0.01 | 0.33 |
| Cer(t34:1) | 553.507 | 7.09 | C34H67N1O4 | M+HCOO | 1.75 | 0.01 | 0.33 |
| PC(38:7) | 803.5465 | 7.09 | C46H78N1O8P1 | M+H | 1.00 | 0.01 | 0.33 |
| Cer(t16:0_18:1) | 553.507 | 7.20 | C34H67N1O4 | M-H | 3.20 | 0.01 | 0.32 |
| PC(17:3_18:1) | 767.5465 | 8.24 | C43H78N1O8P1 | M+H | 3.21 | 0.00 | 0.32 |
| LPG(O-27:0) | 624.473 | 12.32 | C33H69O8P1 | M+Na | 2.26 | 0.00 | 0.32 |
| PC(37:5) | 793.5622 | 8.22 | C45H80N1O8P1 | M+H | 1.09 | 0.01 | 0.31 |
| SPH(d20:0) | 329.3294 | 3.04 | C20H43N1O2 | M+H | 6.59 | 0.00 | 0.30 |
| PE(P-18:1_20:4) | 749.5359 | 7.79 | C43H76N1O7P1 | M+H | 3.00 | 0.01 | 0.30 |
| PC(35:2) | 771.5778 | 7.78 | C43H82N1O8P1 | M+H | 1.13 | 0.01 | 0.29 |
| **Lipid** | **CalcMass** | **RT(min)** | **Formula** | **Ion** | **VIP** | **p** | **FC(L+A/K)** |
| PC(O-36:3) | 769.5985 | 7.79 | C44H84N1O7P1 | M+H | 1.49 | 0.01 | 0.29 |
| LPE(18:0) | 481.3168 | 3.16 | C23H48N1O7P1 | M-H | 1.02 | 0.02 | 0.28 |
| PE(P-18:0_20:4) | 751.5516 | 8.89 | C43H78N1O7P1 | M+H | 1.59 | 0.01 | 0.28 |
| SM(d18:1_18:3) | 724.5519 | 7.09 | C41H77N2O6P1 | M+H | 1.33 | 0.01 | 0.28 |
| Sphinganine(d14:0) | 245.2355 | 1.03 | C14H31N1O2 | M+H | 2.81 | 0.00 | 0.27 |
| PC(40:5) | 835.6091 | 7.93 | C48H86N1O8P1 | M+H | 1.49 | 0.01 | 0.27 |
| Deoxysphinganine(d14:0) | 229.2406 | 1.10 | C14H31N1O1 | M+H | 2.59 | 0.00 | 0.27 |
| PC(P-13:1_20:4) | 721.5046 | 7.81 | C41H72N1O7P1 | M+H | 6.17 | 0.00 | 0.26 |
| PC(20:4_18:0) | 809.5935 | 7.94 | C46H84N1O8P1 | M+H | 3.24 | 0.01 | 0.25 |
| PC(O-38:5) | 793.5985 | 7.46 | C46H84N1O7P1 | M+H | 1.57 | 0.00 | 0.24 |
| SM(d36:1) | 730.5989 | 8.01 | C41H83N2O6P1 | M+H | 1.28 | 0.01 | 0.24 |
| Cer(t20:1_15:0) | 567.5227 | 7.18 | C35H69N1O4 | M+HCOO | 3.58 | 0.01 | 0.23 |
| PC(18:1_20:4) | 807.5778 | 7.05 | C46H82N1O8P1 | M+Na | 1.02 | 0.00 | 0.23 |
| PC(40:8) | 829.5622 | 7.06 | C48H80N1O8P1 | M+H | 1.03 | 0.00 | 0.23 |
| PC(16:0_16:0) | 733.5622 | 7.90 | C40H80N1O8P1 | M+H | 6.54 | 0.01 | 0.20 |
| MG(O-17:1) | 328.2977 | 2.08 | C20H40O3 | M+NH4 | 1.79 | 0.02 | 0.20 |
| MG(O-13:1) | 272.2351 | 1.11 | C16H32O3 | M+NH4 | 4.60 | 0.00 | 0.19 |
| Cer(m18:1_15:0) | 507.5015 | 7.20 | C33H65N1O2 | M+HCOO | 3.74 | 0.02 | 0.17 |
| SPH(d16:0) | 273.2668 | 1.53 | C16H35N1O2 | M+H | 2.32 | 0.03 | 0.17 |
| Sphinganine(d18:0) | 301.2981 | 2.13 | C18H39N1O2 | M+H | 2.48 | 0.00 | 0.14 |
| Deoxysphinganine(d18:0) | 285.3032 | 2.21 | C18H39N1O1 | M+H | 2.47 | 0.00 | 0.13 |
| **Lipid** | **CalcMass** | **RT(min)** | **Formula** | **Ion** | **VIP** | **p** | **FC(L+A/K)** |
| LPG(18:3) | 506.2645 | 2.06 | C24H43O9P1 | M+H | 1.16 | 0.00 | 0.05 |
| AEA(16:1) | 297.2668 | 1.60 | C18H35N1O2 | M+H | 1.08 | 0.00 | 0.02 |
| SPH(d18:2) | 281.2719 | 3.10 | C18H35N1O1 | M+H | 5.95 | 0.00 | 0.01 |

FC (L+A/K): Fold change of lipid abundance in LPS+ATP+K-SAC group (K) versus LPS+ATP group (L+A). Statistical significance was calculated via unpaired t test, two-tailed.
